# National, regional, and global cardiomyopathy burden from 1990 to 2019

**DOI:** 10.3389/fcvm.2022.1042448

**Published:** 2022-11-30

**Authors:** Siyuan Cheng, Yuchen Han, Lie Jiang, Ziyin Lan, Jun Guo

**Affiliations:** Department of Cardiology, The First Affiliated Hospital of Jinan University, Guangzhou, China

**Keywords:** cardiomyopathy, alcoholic cardiomyopathy, other cardiomyopathy, global burden of disease (GBD), global public health

## Abstract

**Objective:**

To examine the incidence of cardiomyopathy including both alcoholic cardiomyopathy (AC) and other cardiomyopathy (OC) in 204 nations and regions over the 1990–2019 period.

**Methods:**

The present study was conducted using data derived from the GBD 2019 study coordinated by the Institute for Health Metrics and Evaluation (IHME). The GBD 2019 study included epidemiological data pertaining to 369 diseases/injuries, 286 causes of death, and 87 risk factors in 204 nations and regions. For this study, we adopt published estimates pertaining to the prevalence rates, mortality rates, and disability-adjusted life years (DALYs) associated with cardiomyopathy. The Bayesian mixed-effects DisMod-MR 2.1 meta-regression tool, which was designed to analyze GBD data, was used to estimate the prevalence of OC and AC. The GBD data are subdivided into 21 global regions based on characteristics such as geographical proximity and epidemiological similarity. The overall burden of cardiomyopathy was assessed by combining AC- and OC-related data, 95% confidence intervals were calculated based on standardized error values determined based upon the width of the 95% UI divided by 1.96 × 2.

**Results:**

Globally, there were an estimated 0.71 million (95% UI: 0.55–0.92) AC cases and 3.73 million (95% UI: 2.92–4.72) OC cases in 2019. The age-standardized cardiomyopathy, AC, and OC prevalence rates (per 100,000 persons) in 2019 were 56.0 (95% CI: 43.82–71.17), 8.51 (95% UI: 6.6–11.01), and 47.49 (95% UI: 37.22–60.16), respectively. In total, the respective numbers of global deaths attributed to AC and OC were 0.07 million (95% UI: 0.06–0.08) and 0.24 million (95% UI: 0.19–0.26). The age-standardized mortality rate for cardiomyopathy in 2019 was 3.97 (95% CI: 3.29–4.39), with respective mortality rates of 0.86 (95% UI: 0.72–0.99) and 3.11 (95% UI: 2.57–3.4) for AC and OC. At the global level in 2019, 2.44 million (95% UI: 2.04–2.78) DALYs were attributed to AC, while 5.72 million (95% UI: 4.89–6.33) DALYs were attributed to OC. From 1990 to 2019, cardiomyopathy age-standardized prevalence rates declined by −0.49% (95% CI: −0.57 to −0.41), with those for AC and OC having respectively declined by −0.32% (95% UI: −0.36 to −0.28) and −0.17% (95% UI: −0.21 to −0.13). The age-standardized AC and OC mortality rates declined by −0.36% (95% UI: −0.5 to −0.26) and −0.39% (95% UI: −0.44 to −0.29), despite 24.8 and 30.2% increases, respectively, in the numbers of AC- and OC-related deaths during the same period.

**Conclusion:**

Previous studies have estimated the risk factors that influence the burden of multiple cardiovascular diseases (CVD). Among them, some studies related to the GBD database on cardiomyopathy data suggest that alcohol intake, gender are factors in the development of AC, and the burden of AC and OC is not limited to developed or less developed countries. Otherwise, this study mainly focused on cardiomyopathy, and analyzed multiple indicators from national, regional, and age-standard dimensions to identify potential risk factors including prevalence, deaths, years lived with Disability-adjusted life years (DALYs) that influence the development of AC and OC. To our knowledge, this study is the first to have systematically assessed the burden of AC and OC as of 2019 at the national, regional, and global levels and calculated DALYs to achieve a better evaluation of disease risk and quality of life of the population. The number of cases, deaths and DALYs of cardiomyopathy showed an overall increasing trend and obvious geographical differences in the past three decades. The burden of cardiomyopathy remains a persistent threat to global public health. These results provide an epidemiological foundation that can guide public health efforts and policymakers.

## Introduction

Cardiovascular disease (CVD) is the most prominent driver of global morbidity and mortality, resulting in high rates of disability or lost productivity that impose a marked burden on affected patients and society as a whole (1). Cardiomyopathy is an uncommon and thus often-overlooked yet severe form of CVD that is characterized by clinical findings including abnormal cardiac structural characteristics, arrhythmia, and/or heart failure (2). Cardiomyopathy can result in the sudden death of affected individuals during childhood or adolescence and may require patients to undergo cardiac transplantation in some cases (3). Among the all types of cardiomyopathy, alcoholic cardiomyopathy (AC) is a special one. It is caused by heavy alcohol use over a prolonged period. Alcohol and its metabolites are cardiotoxic. Myocardial inhibition secondary to alcohol is initially reversible, but prolonged alcohol consumption can lead to irreversible dysfunction (4). However, there is neither a certain amount of alcohol known to be toxic to myocardial cells nor is there a special period of exposure time to cause it to happen (5). Furthermore, not all chronic alcohol abusers develop AC finally and prevalence of AC still cannot be accurately assessed due to incomplete data and its uncertainty (6).

A comprehensive overview of the epidemiological characteristics of cardiomyopathy in particular populations and countries would be invaluable as a tool for guiding the development of public health policy and informing decision-making aimed at preventing, diagnosing, and treating this debilitating disease. However, robust datasets pertaining to cardiomyopathy morbidity and mortality are primarily available for highly developed nations that had standardized the diagnosis and evaluation of affected patients (7). The Global Burden of Disease (GBD) study, which is an ongoing analysis of the incidence of over 350 diseases in around 195 nations and regions throughout the world, offers a unique opportunity to gain a more robust overview of the true global burden of cardiomyopathy. Since 2016, the GBD study has subdivided cardiomyopathy into the alcoholic cardiomyopathy (AC) and other cardiomyopathy (OC) subtypes (8). There is thus a clear need to more fully explore the national, regional, and global AC and OC burden and to explore trends in cardiomyopathy-related disease findings as a function of time.

## Materials and methods

### Data sources

The present study was conducted using data derived from the GBD study coordinated by the Institute for Health Metrics and Evaluation (IHME). Each annual GBD study update entails the re-estimation of the entire data time series based on new advances in medical knowledge, modeling, estimation approaches, and data in an effort to ensure the continuous improvement of study quality. The GBD 2019 study was published in October 2020 and included epidemiological data pertaining to 369 diseases/injuries, 286 causes of death, and 87 risk factors in 204 nations and regions, with sub-national estimates additionally being reported for a subset of countries. For the present study, we obtained published estimates pertaining to the prevalence rates, mortality rates, and disability-adjusted life years (DALYs) associated with cardiomyopathy (9). As the GBD 2019 study consists of aggregated, de-identified data, the University of Washington Institutional Review Board approved a waiver of informed consent with respect to the use of these data for research purposes.

### Global burden of disease estimation framework

AC and OC-related epidemiological data were established based on the 9th and 10th edition of the International Classification of Diseases (ICD-9 and ICD-10), with AC corresponding to diseases coded as 425.5 (ICD-9) or I42.6 (ICD-10), whereas OC corresponded to diseases coded as 425.0–425.3, 425.7–425.8, and 429.0 (ICD-9) or I42.1–I42.5, I42.7–I42.8, and I43–I43.9 (ICD-10).

Estimates of mortality rates were obtained from vital registration data sources for both OC and AC and from verbal autopsy data sources for AC using the ICD codes detailed above. The standard Cause of Death Ensemble model (CODEm), which incorporates a range of covariates and individual models (including linear mixed-effects regression and spatiotemporal Gaussian process regression models) to generate cause of death (COD) predictions, was used to estimate rates of AC- and OC-related mortality. Out-of-sample predictive validity tests were employed to evaluate utilized individual and ensemble models, with experts in appropriate disease fields having vetted these models in addition to their having been validated by the IHME and international collaborators. Estimated mortality rates were scaled based on other COD-related estimates to yield all-cause mortality estimates summing to 100% within individual year, age, sex, and location groups (10). As mortality-related data availability was limited for some nations, it was converted from incidence data through mortality-to-incidence ratio modeling.

The Bayseian mixed-effects DisMod-MR 2.1 meta-regression tool, which was designed to analyze GBD data, was used to estimate the prevalence of OC and AC (11). Consistent prevalence estimates for all locations were generated by integrating the incidence, remission, and mortality rates for particular causes. Prevalence estimates did not incorporate non-literature data sources other than hospital or claim data. Outpatient data were excluded from these analyses as they were available at substantially lower levels than corresponding inpatient and claim data from matched locations. For high-income nations, inpatient hospital data found to be either 2-fold above or 0.5-fold below the median absolute deviation value for the corresponding age-sex group were additionally excluded (11).

DALYs were measured by summing both years lost in life (YLLs) and years lost due to disability (YLDs), providing a metric for health losses associated with a particular disease state. YLLs were quantified by multiplying estimated numbers of deaths by age with the corresponding standard life expectancy for individuals in that age group. YLDs were quantified by multiplying the number of cases of a given disease by the disability weight (ranging from 0 to 1, with 0 and 1 respectively corresponding to normal health and death), thus providing a metric proportional to the degree of health lost as a consequence of a given disease state (12). Total DALYs within a given population offer substantial value as a means of measuring the overall disease burden experienced by that population.

Data from the GBD are subdivided into 21 global regions based on characteristics including geographical proximity and epidemiological similarity. The GBD study additionally reports the socio-demographic index (SDI) values for included regions at the state level, corresponding to a metric for average per-capita income, total fertility rates, and educational attainment (11, 13–15). SDI values range from 0 to 1, with larger values corresponding to higher levels of sociodemographic development such that nations are grouped into SDI-based development quintiles (high, high-middle, middle, low-middle, and low) (11).

Age-standardized rate (ASR) values were established based upon the world standard population developed for the GBD study. And the fold differences in rates calculated by subtracting the maximum value of the corresponding index from the minimum value. GBD disease burden estimates were reported with 95% uncertainty intervals (UIs) that take into account differences and uncertainties in parameter estimation, model selection, data collection, and other factors.

### Data analysis

The overall burden of cardiomyopathy was assessed by combining AC- and OC-related data. As no published UI estimates corresponding to cardiomyopathy were available, 95% confidence intervals were calculated based on standardized error values determined based upon the width of the 95% UI divided by 1.96 × 2.

Trends in ASRs correspond to shifts in disease-related patterns within particular populations, providing insight into potential underlying shifts in risk factor prevalence (16). Trends in cardiomyopathy incidence, prevalence, and DALYs were evaluated based on percentage changes calculated based upon available estimates for the selected timepoints. An ASR was considered to be increased or decreased if the entirety of the 95% UI was above or below 0, respectively. If the 95% UI included 0, then the ASR was deemed stable. Estimated annual percentage changes served as a measure of ASR trends over particular time intervals, and were calculated as previously reported by Hankey et al. (17).

R (v 3.4.4, R core team) was used for all statistical analyses, with P < 0.05 as the threshold of significance.

## Results

### Cardiomyopathy prevalence in 2019

Globally, there were an estimated 0.71 million (95% UI: 0.55–0.92) AC cases and 3.73 million (95% UI: 2.92–4.72) OC cases in 2019. Respective age-standardized cardiomyopathy, AC, and OC prevalence rates (per 100,000 persons) in 2019 were 56.0 (95% CI: 43.82–71.17), 8.51 (95% UI: 6.6–11.01), and 47.49 (95% UI: 37.22–60.16), respectively (Tables 1–3). When ASRs were compared among different SDI groups, the highest cardiomyopathy ASR was observed in the Low SDI group (126.1 per 100,000 persons; 95% CI: 87.35–173.38), with the highest OC ASR similarly being observed in the Low SDI group (123.96 per 100,000 persons; 95% UI: 85.91–170.34). In contrast, the highest AC ASR was observed in the High SDI group (23.7 per 100,000 persons; 95% UI: 18.87–29.97) in 2019 (Tables 1–3).

**TABLE 1:** Age-standardized prevalence, death, DALYs rates of the cardiomyopathy, and their percentage changes with 95% CIs from 1990 to 2019, both sexes.

Region	Prevalence			Deaths			DALYs		
	1990 age-standardized rate per 100,000 people	2019 age-standardized rate per 100,000 people	Percentage change in age-standardized rates, 1990–2019	1990 age-standardized rate per 100,000 people	2019 age-standardized rate per 100,000 people	Percentage change in age-standardized rates, 1990–2019	1990 age-standardized rate per 100,000 people	2019 age-standardized rate per 100,000 people	Percentage change in age-standardized rates, 1990–2019
Global	69.8 (53.79 to 89.65)	56 (43.82 to 71.17)	−0.49 (−0.57 to −0.41)	6.4 (5.05 to 7.22)	3.97 (3.29 to 4.39)	−0.75 (−0.94 to −0.56)	142.86 (127.03 to 164.97)	101.95 (86.71 to 113.79)	−0.56 (−0.83 to −0.38)
**SDI**									
High SDI	131.5 (103.46 to 166.12)	111.37 (90.83 to 136)	−0.33 (−0.46 to −0.18)	7.07 (5.34 to 7.87)	4.04 (3.48 to 4.66)	−0.9 (−1.1 to −0.63)	172.09 (136.7 to 189.9)	100.14 (89.61 to 115.07)	−0.87 (−1.04 to −0.63)
High-middle SDI	67.42 (51.04 to 87.86)	57.04 (44.05 to 73.36)	−0.41 (−0.49 to −0.33)	11.26 (7.74 to 13.3)	7.36 (5.42 to 8.39)	−0.57 (−0.89 to −0.27)	235.39 (199.95 to 266.55)	207.91 (157.94 to 262.92)	−0.27 (−0.77 to 0.11)
Middle SDI	29.76 (22.67 to 38.07)	26.5 (20.54 to 33.89)	−0.14 (−0.2 to −0.06)	2.69 (2.24 to 3.35)	2.11 (1.74 to 2.49)	−0.49 (−0.84 to −0.15)	69.87 (58.93 to 95.34)	53.39 (45.15 to 66.13)	−0.48 (−0.87 to −0.11)
Low-middle SDI	27.95 (20.54 to 37)	25.65 (18.6 to 34.25)	−0.01 (−0.11 to 0.1)	2.39 (1.89 to 2.95)	1.88 (1.58 to 2.25)	−0.49 (−0.82 to −0.12)	58.91 (46.71 to 75.09)	49.35 (41.35 to 58.63)	−0.39 (−0.73 to 0.01)
Low SDI	127.21 (86.32 to 174.38)	126.1 (87.35 to 173.38)	−0.01 (−0.06 to 0.05)	4.37 (2.74 to 6.05)	3.27 (2.43 to 4.11)	−0.7 (−1 to −0.2)	132.67 (84.43 to 176.1)	101.22 (77.99 to 125.71)	−0.68 (−1 to −0.2)
**Region**									
High-income Asia Pacific	77.48 (58.43 to 100.95)	68.16 (53.9 to 85.34)	−0.32 (−0.5 to −0.1)	4.67 (2.6 to 5.26)	1.96 (1.39 to 2.22)	−1.18 (−1.35 to −0.89)	98.83 (75.8 to 107.28)	53.63 (45.38 to 60.56)	−1.1 (−1.26 to −0.92)
High-income North America	198.53 (156.46 to 253.35)	198.35 (158.03 to 250.13)	−0.19 (−0.38 to 0.04)	7.88 (6.03 to 8.72)	5.64 (4.94 to 6.46)	−0.69 (−0.89 to −0.33)	197.06 (157.12 to 218.2)	181.01 (155.27 to 207.31)	−0.74 (−0.91 to −0.39)
Western Europe	104.96 (82.12 to 131.7)	85.62 (67.2 to 106.69)	−0.38 (−0.49 to −0.27)	10.38 (6.02 to 11.92)	4.17 (3.55 to 5.08)	−1.16 (−1.35 to −0.69)	185.4 (130.04 to 208.51)	82.25 (72.11 to 100.31)	−1.1 (−1.28 to −0.74)
Australasia	140.35 (106.9 to 183.36)	121.2 (92 to 157.68)	−0.56 (−0.69 to −0.42)	7.03 (4.64 to 8.13)	3.25 (2.69 to 4.16)	−1 (−1.27 to −0.51)	177.72 (131.64 to 202.86)	86.18 (72.52 to 108.23)	−0.98 (−1.22 to −0.6)
Andean Latin America	15.32 (11.02 to 21.18)	18.43 (13.69 to 24.28)	0.52 (0.26 to 0.84)	1.89 (1.4 to 2.36)	1.43 (1.1 to 1.86)	−0.79 (−1.14 to −0.22)	58.62 (40.76 to 76.29)	40.63 (30.69 to 51.99)	−0.83 (−1.19 to −0.22)
Tropical Latin America	82.96 (59.24 to 113.56)	80.18 (57.35 to 108.33)	0.02 (−0.09 to 0.16)	15.34 (11.8 to 16.91)	8.99 (7.83 to 10.55)	−1.07 (−1.2 to −0.82)	341.78 (268.13 to 369.21)	260.47 (226.77 to 300.64)	−1.05 (−1.17 to −0.83)
Central Latin America	19.96 (14.78 to 26.55)	19.24 (14.49 to 25.22)	−0.05 (−0.14 to 0.06)	2.26 (1.96 to 2.53)	1.83 (1.49 to 2.21)	−0.74 (−0.98 to −0.46)	54.63 (48.82 to 61.52)	54.42 (42.8 to 65.49)	−0.68 (−0.95 to −0.37)
Southern Latin America	76.56 (53.28 to 105.77)	81.86 (58.83 to 112.74)	0.14 (−0.1 to 0.42)	12.57 (9.19 to 14.84)	8.55 (7.15 to 9.53)	−1.07 (−1.25 to −0.81)	279.4 (221.29 to 321.58)	176.58 (157.66 to 202.53)	−1.1 (−1.25 to −0.86)
Caribbean	36.43 (27.57 to 47.65)	38.69 (29.31 to 51)	0.16 (−0.01 to 0.35)	4.74 (3.86 to 6.18)	5.05 (4.03 to 6.31)	0.41 (−0.15 to 1.02)	147.09 (113.62 to 203.11)	154.76 (116.57 to 206.84)	0.31 (−0.23 to 0.95)
Central Europe	104.96 (77.69 to 139.43)	105.71 (80.36 to 138.28)	0.1 (−0.1 to 0.33)	16.76 (12.39 to 18.93)	12.95 (9.19 to 15.11)	−0.37 (−0.67 to −0.12)	312.7 (244.25 to 347.08)	252.04 (193.3 to 293.86)	−0.27 (−0.63 to 0.03)
Eastern Europe	76.32 (56.8 to 100.11)	72.82 (53.49 to 96.79)	−0.09 (−0.18 to −0.01)	13.73 (10.78 to 20.84)	23.53 (16.33 to 28.42)	1.77 (−0.23 to 3.3)	462.59 (350.72 to 730.04)	845.89 (620.74 to 1019.77)	2.12 (−0.11 to 4.11)
Central Asia	34.1 (25.48 to 44.95)	30.74 (23 to 40.44)	−0.25 (−0.36 to −0.12)	4.77 (3.98 to 6.78)	10.23 (5.87 to 12.37)	1.57 (−0.19 to 2.76)	148.52 (124.54 to 223.63)	303.63 (179.96 to 370.54)	1.53 (−0.27 to 2.73)
North Africa and Middle East	26.55 (20.2 to 34.67)	27.05 (20.91 to 34.77)	0.02 (−0.08 to 0.12)	3.25 (2.34 to 4.87)	2.22 (1.8 to 3.15)	−0.86 (−1.22 to −0.36)	104.16 (71.26 to 163.23)	64.25 (50.95 to 90.74)	−0.89 (−1.27 to −0.35)
South Asia	1.84 (1.28 to 2.57)	1.91 (1.33 to 2.66)	0.07 (0.02 to 0.11)	0.49 (0.26 to 0.83)	0.4 (0.29 to 0.55)	−0.4 (−0.87 to 0.4)	9.75 (6.3 to 14.9)	11.48 (6.78 to 17.32)	−0.36 (−0.82 to 0.46)
Southeast Asia	39.78 (30.34 to 51.27)	42.76 (32.86 to 54.9)	0.17 (0.08 to 0.27)	3.75 (2.73 to 4.64)	3.5 (2.57 to 4.24)	−0.13 (−0.64 to 0.51)	86.06 (69 to 125.95)	79.23 (63.72 to 104.05)	−0.09 (−0.64 to 0.59)
East Asia	11.5 (8.73 to 14.82)	12.17 (9.18 to 15.66)	0.14 (0.05 to 0.22)	0.92 (0.58 to 1.76)	0.86 (0.62 to 1.11)	0.07 (−1.07 to 1.9)	32.64 (21.49 to 59.3)	23.6 (17.6 to 32.1)	−0.01 (−1.07 to 1.56)
Oceania	21.51 (15.95 to 28.67)	22.06 (16.3 to 29.53)	0.07 (−0.09 to 0.23)	3.26 (2.17 to 4.91)	3.33 (2.15 to 4.75)	−0.33 (−0.71 to 0.21)	97.18 (60.3 to 152.73)	101.43 (60.9 to 158.87)	−0.28 (−0.7 to 0.3)
Western Sub-Saharan Africa	209.02 (144.96 to 284.52)	226.43 (151.61 to 315.46)	0.09 (−0.01 to 0.17)	8.56 (4.92 to 11.79)	5.74 (4.17 to 7.07)	−0.94 (−1.23 to −0.4)	220.77 (132.86 to 296.02)	153.61 (115.41 to 190.35)	−0.89 (−1.18 to −0.36)
Eastern Sub-Saharan Africa	247.19 (169.4 to 336.77)	266.89 (186.17 to 360.49)	0.15 (0.07 to 0.23)	4.52 (3.12 to 5.81)	3.78 (2.61 to 4.72)	−0.55 (−0.99 to 0.12)	171.32 (117 to 219.56)	139.66 (98.36 to 181.44)	−0.53 (−0.99 to 0.17)
Central Sub-Saharan Africa	212.69 (138.42 to 302.94)	209.24 (135.52 to 296.96)	−0.03 (−0.17 to 0.13)	9.79 (5.28 to 14.71)	8.62 (5 to 13.98)	−0.46 (−0.96 to 0.35)	273.15 (149.89 to 385.83)	232.96 (143.1 to 351.42)	−0.47 (−0.96 to 0.34)
Southern Sub-Saharan Africa	263.43 (178.98 to 363.03)	240.51 (160.28 to 335.45)	−0.17 (−0.24 to −0.09)	12.46 (9.74 to 14.99)	11.3 (9.31 to 14.15)	−0.32 (−0.58 to −0.01)	295.5 (237.46 to 346.93)	253.69 (210.2 to 311.86)	−0.38 (−0.64 to −0.07)

DALY, disability-adjusted life year; CI, confidence interval.

**TABLE 2:** Age-standardized prevalence, death, DALYs rates of the alcoholic cardiomyopathy, and their percentage changes with 95% UIs from 1990 to 2019, both sexes.

Region	Prevalence			Deaths			DALYs		
	1990 age-standardized rate per 100,000 people	2019 age-standardized rate per 100,000 people	Percentage change in age-standardized rates, 1990–2019	1990 age-standardized rate per 100,000 people	2019 age-standardized rate per 100,000 people	Percentage change in age-standardized rates, 1990–2019	1990 age-standardized rate per 100,000 people	2019 age-standardized rate per 100,000 people	Percentage change in age-standardized rates, 1990–2019
Global	12.6 (9.6 to 16.45)	8.51 (6.6 to 11.01)	−0.32 (−0.36 to −0.28)	1.34 (1.23 to 1.69)	0.86 (0.72 to 0.99)	−0.36 (−0.5 to −0.26)	40.22 (36.62 to 52.06)	29.2 (24.51 to 33.31)	−0.27 (−0.46 to −0.16)
**SDI**									
High SDI	29.12 (22.37 to 38.1)	23.7 (18.87 to 29.97)	−0.19 (−0.25 to −0.11)	1.6 (1.2 to 1.96)	0.81 (0.65 to 0.98)	−0.49 (−0.63 to −0.37)	47.59 (37.16 to 56.34)	25.18 (20.67 to 29.68)	−0.47 (−0.59 to −0.36)
High-middle SDI	17.88 (13.16 to 24.11)	12.14 (9.04 to 16.22)	−0.32 (−0.35 to −0.29)	3.03 (2.64 to 4.19)	2.58 (2.12 to 3.03)	−0.15 (−0.41 to 0.04)	98.64 (81.98 to 139.36)	94.53 (78.17 to 111.01)	−0.04 (−0.36 to 0.23)
Middle SDI	1.8 (1.34 to 2.4)	1.76 (1.31 to 2.34)	−0.02 (−0.05 to 0.01)	0.22 (0.17 to 0.3)	0.16 (0.12 to 0.19)	−0.28 (−0.5 to −0.06)	6.67 (5.27 to 8.91)	5 (3.84 to 6.18)	−0.25 (−0.47 to −0.03)
Low-middle SDI	1.45 (1.05 to 1.97)	1.57 (1.13 to 2.14)	0.08 (0.04 to 0.13)	0.31 (0.2 to 0.45)	0.22 (0.17 to 0.27)	−0.29 (−0.49 to −0.02)	8.99 (6.14 to 12.87)	6.78 (5.27 to 8.13)	−0.25 (−0.45 to 0.02)
Low SDI	2.15 (1.44 to 3.06)	2.15 (1.44 to 3.04)	0 (−0.03 to 0.03)	0.3 (0.19 to 0.47)	0.16 (0.11 to 0.23)	−0.46 (−0.6 to −0.24)	8.85 (5.69 to 13.81)	4.76 (3.5 to 6.79)	−0.46 (−0.6 to −0.25)
**Region**									
High-income Asia Pacific	6.74 (4.86 to 9.42)	5.58 (4.27 to 7.27)	−0.17 (−0.26 to −0.06)	0.24 (0.18 to 0.28)	0.1 (0.07 to 0.12)	−0.6 (−0.72 to −0.5)	8.49 (6.93 to 10.06)	3.64 (2.72 to 4.5)	−0.57 (−0.68 to −0.48)
High-income North America	47.11 (35.51 to 64.2)	40.38 (31.68 to 51.78)	−0.14 (−0.24 to −0.03)	2.01 (1.45 to 2.43)	1.08 (0.88 to 1.34)	−0.46 (−0.6 to −0.27)	63.89 (48.3 to 74.86)	34.49 (29.52 to 42.17)	−0.46 (−0.56 to −0.3)
Western Europe	22.16 (17.08 to 28.46)	17.75 (13.71 to 22.48)	−0.2 (−0.26 to −0.14)	1.66 (1.26 to 2.12)	0.74 (0.59 to 0.9)	−0.56 (−0.69 to −0.44)	46.68 (36.52 to 56.73)	21.44 (17.39 to 25.55)	−0.54 (−0.66 to −0.42)
Australasia	49.51 (36.7 to 65.58)	37.04 (27.51 to 49.63)	−0.25 (−0.32 to −0.18)	1.84 (1.43 to 2.3)	1.07 (0.8 to 1.36)	−0.42 (−0.62 to −0.24)	58.57 (46.93 to 71.18)	34.39 (26.8 to 42.02)	−0.41 (−0.59 to −0.27)
Andean Latin America	0.58 (0.38 to 0.86)	0.75 (0.52 to 1.05)	0.29 (0.15 to 0.49)	0.11 (0.07 to 0.17)	0.05 (0.04 to 0.07)	−0.57 (−0.73 to −0.28)	2.9 (1.82 to 4.09)	1.34 (0.96 to 1.83)	−0.54 (−0.7 to −0.24)
Tropical Latin America	4.28 (2.77 to 6.23)	4.51 (3.05 to 6.5)	0.05 (−0.01 to 0.14)	1.57 (1.22 to 2.01)	0.49 (0.37 to 0.61)	−0.69 (−0.76 to −0.61)	54.82 (42.37 to 68.97)	17.53 (13.57 to 21.57)	−0.68 (−0.74 to −0.6)
Central Latin America	1.35 (0.92 to 1.92)	1.33 (0.92 to 1.87)	−0.01 (−0.06 to 0.05)	0.31 (0.23 to 0.38)	0.12 (0.09 to 0.15)	−0.62 (−0.71 to −0.51)	8.86 (6.64 to 10.45)	3.78 (2.9 to 4.78)	−0.57 (−0.68 to −0.45)
Southern Latin America	4.41 (2.87 to 6.41)	4.7 (3.11 to 6.67)	0.07 (−0.05 to 0.2)	1.18 (0.89 to 1.53)	0.23 (0.17 to 0.31)	−0.8 (−0.86 to −0.74)	35.41 (26.95 to 45.4)	7.41 (5.68 to 9.47)	−0.79 (−0.85 to −0.73)
Caribbean	10.39 (7.5 to 14)	11.68 (8.35 to 15.94)	0.12 (0.01 to 0.25)	1.04 (0.81 to 1.44)	1.52 (1.2 to 1.85)	0.45 (0.08 to 0.89)	33.76 (25.61 to 46.57)	45.48 (36.37 to 55.73)	0.35 (0.02 to 0.75)
Central Europe	20.18 (14.89 to 27.08)	22.47 (17.19 to 29.4)	0.11 (0.01 to 0.24)	2.7 (2.25 to 3.3)	2.37 (1.71 to 2.86)	−0.12 (−0.33 to 0.05)	73.69 (62.14 to 87.51)	72.56 (51.08 to 87.16)	−0.02 (−0.27 to 0.19)
Eastern Europe	51.92 (37.71 to 69.31)	49.54 (35.45 to 66.98)	−0.05 (−0.09 to −0.01)	9.97 (7.85 to 14.4)	15.02 (12.29 to 17.94)	0.51 (0 to 1.02)	356.14 (272.01 to 518.48)	580.58 (476.55 to 688.97)	0.63 (0.08 to 1.26)
Central Asia	3.86 (2.8 to 5.24)	3.26 (2.39 to 4.46)	−0.16 (−0.21 to −0.1)	0.76 (0.64 to 0.92)	0.95 (0.56 to 1.34)	0.25 (−0.24 to 0.75)	24.12 (20.86 to 31.08)	32.59 (19.19 to 45.74)	0.35 (−0.25 to 0.84)
North Africa and Middle East	1.77 (1.28 to 2.45)	1.77 (1.3 to 2.41)	0 (−0.05 to 0.05)	0.27 (0.14 to 0.47)	0.12 (0.08 to 0.16)	−0.56 (−0.76 to −0.26)	6.99 (3.82 to 11.01)	3.38 (2.3 to 4.35)	−0.52 (−0.72 to −0.22)
South Asia	0.71 (0.49 to 0.99)	0.72 (0.5 to 1.02)	0.02 (0 to 0.04)	0.22 (0.1 to 0.37)	0.14 (0.1 to 0.2)	−0.35 (−0.54 to 0.06)	5.81 (2.68 to 9.68)	3.91 (2.76 to 5.47)	−0.33 (−0.51 to 0.09)
Southeast Asia	1.71 (1.31 to 2.22)	1.88 (1.45 to 2.43)	0.1 (0.05 to 0.15)	0.16 (0.11 to 0.24)	0.15 (0.09 to 0.22)	−0.06 (−0.39 to 0.36)	4.76 (3.21 to 7.46)	4.75 (3.12 to 7.46)	0 (−0.35 to 0.45)
East Asia	1.74 (1.29 to 2.32)	1.88 (1.38 to 2.5)	0.08 (0.04 to 0.12)	0.11 (0.06 to 0.24)	0.13 (0.09 to 0.18)	0.16 (−0.52 to 1.38)	3.64 (2.1 to 7.14)	4.65 (3.15 to 6.09)	0.28 (−0.44 to 1.36)
Oceania	1.32 (0.93 to 1.82)	1.37 (0.98 to 1.89)	0.04 (−0.04 to 0.13)	0.29 (0.15 to 0.54)	0.18 (0.1 to 0.3)	−0.39 (−0.56 to −0.14)	8 (4.25 to 14.14)	5.12 (2.73 to 8.67)	−0.36 (−0.53 to −0.1)
Western Sub-Saharan Africa	5.03 (3.22 to 7.4)	5.04 (3.12 to 7.59)	0 (−0.05 to 0.05)	0.55 (0.34 to 0.83)	0.21 (0.14 to 0.29)	−0.63 (−0.74 to −0.44)	18.28 (11.36 to 27.11)	6.98 (4.8 to 9.59)	−0.62 (−0.74 to −0.42)
Eastern Sub-Saharan Africa	2.09 (1.4 to 2.97)	2.23 (1.5 to 3.11)	0.06 (0.03 to 0.1)	0.06 (0.03 to 0.12)	0.04 (0.02 to 0.07)	−0.39 (−0.63 to 0.01)	2.15 (1.32 to 4.08)	1.41 (0.69 to 2.35)	−0.35 (−0.59 to 0.06)
Central Sub-Saharan Africa	1.64 (1.06 to 2.44)	1.62 (1.03 to 2.41)	−0.01 (−0.08 to 0.07)	0.12 (0.07 to 0.2)	0.08 (0.04 to 0.15)	−0.34 (−0.62 to 0.12)	3.85 (2.12 to 6.49)	2.6 (1.43 to 4.82)	−0.32 (−0.59 to 0.13)
Southern Sub-Saharan Africa	0.54 (0.39 to 0.71)	0.49 (0.35 to 0.67)	−0.08 (−0.12 to −0.04)	0.04 (0.03 to 0.05)	0.03 (0.02 to 0.04)	−0.22 (−0.37 to −0.06)	1.27 (1 to 1.67)	0.96 (0.73 to 1.28)	−0.24 (−0.39 to −0.06)

DALY, disability-adjusted life year; UI, uncertainty interval.

**TABLE 3:** Age-standardized prevalence, death, DALYs rates of other cardiomyopathy, and their percentage changes with 95% UIs from 1990 to 2019, both sexes.

Region	Prevalence			Deaths			DALYs		
	1990 age-standardized rate per 100,000 people	2019 age-standardized rate per 100,000 people	Percentage change in age-standardized rates, 1990–2019	1990 age-standardized rate per 100,000 people	2019 age-standardized rate per 100,000 people	Percentage change in age-standardized rates, 1990–2019	1990 age-standardized rate per 100,000 people	2019 age-standardized rate per 100,000 people	Percentage change in age-standardized rates, 1990–2019
Global	57.2 (44.19 to 73.2)	47.49 (37.22 to 60.16)	−0.17 (−0.21 to −0.13)	5.07 (3.82 to 5.53)	3.11 (2.57 to 3.4)	−0.39 (−0.44 to −0.29)	102.64 (90.41 to 112.91)	72.75 (62.2 to 80.48)	−0.29 (−0.37 to −0.22)
**SDI**									
High SDI	102.38 (81.09 to 128.02)	87.67 (71.96 to 106.02)	−0.14 (−0.21 to −0.07)	5.47 (4.14 to 5.91)	3.24 (2.82 to 3.68)	−0.41 (−0.46 to −0.26)	124.5 (99.54 to 133.57)	74.97 (68.94 to 85.39)	−0.4 (−0.45 to −0.27)
High-middle SDI	49.55 (37.87 to 63.76)	44.9 (35.01 to 57.14)	−0.09 (−0.14 to −0.04)	8.23 (5.1 to 9.11)	4.78 (3.3 to 5.36)	−0.42 (−0.47 to −0.3)	140.86 (121.78 to 155.54)	109.27 (75.96 to 123.56)	−0.22 (−0.41 to −0.12)
Middle SDI	27.96 (21.32 to 35.67)	24.74 (19.23 to 31.55)	−0.12 (−0.15 to −0.07)	2.47 (2.07 to 3.05)	1.95 (1.62 to 2.29)	−0.21 (−0.34 to −0.09)	63.2 (53.65 to 86.42)	48.39 (41.31 to 59.96)	−0.23 (−0.4 to −0.08)
Low-middle SDI	26.5 (19.49 to 35.04)	24.08 (17.47 to 32.11)	−0.09 (−0.14 to −0.03)	2.09 (1.69 to 2.49)	1.67 (1.41 to 1.98)	−0.2 (−0.33 to −0.1)	49.93 (40.56 to 62.22)	42.57 (36.08 to 50.5)	−0.15 (−0.29 to −0.01)
Low SDI	125.06 (84.88 to 171.33)	123.96 (85.91 to 170.34)	−0.01 (−0.04 to 0.02)	4.07 (2.56 to 5.58)	3.11 (2.31 to 3.89)	−0.24 (−0.4 to 0.03)	123.82 (78.74 to 162.3)	96.45 (74.48 to 118.92)	−0.22 (−0.4 to 0.06)
**Region**									
High-income Asia Pacific	71.9 (54.16 to 93.68)	61.43 (49.04 to 75.91)	−0.15 (−0.24 to −0.04)	4.43 (2.42 to 4.97)	1.86 (1.32 to 2.1)	−0.58 (−0.63 to −0.39)	95.19 (73.08 to 102.78)	45.14 (38.45 to 50.5)	−0.53 (−0.58 to −0.44)
High-income North America	158.15 (124.77 to 201.57)	151.24 (122.52 to 185.93)	−0.04 (−0.14 to 0.07)	5.87 (4.58 to 6.29)	4.57 (4.06 to 5.12)	−0.22 (−0.29 to −0.06)	162.57 (127.6 to 176.04)	117.12 (106.97 to 132.44)	−0.28 (−0.35 to −0.09)
Western Europe	82.81 (65.04 to 103.24)	67.87 (53.48 to 84.21)	−0.18 (−0.23 to −0.13)	8.72 (4.76 to 9.8)	3.43 (2.96 to 4.18)	−0.61 (−0.66 to −0.25)	138.72 (93.52 to 151.78)	60.81 (54.72 to 74.76)	−0.56 (−0.62 to −0.32)
Australasia	103.31 (79.39 to 133.72)	71.69 (55.3 to 92.1)	−0.31 (−0.37 to −0.24)	5.19 (3.21 to 5.84)	2.19 (1.89 to 2.8)	−0.58 (−0.65 to −0.27)	119.15 (84.71 to 131.68)	51.78 (45.72 to 66.21)	−0.57 (−0.63 to −0.33)
Andean Latin America	14.57 (10.49 to 20.13)	17.85 (13.3 to 23.43)	0.22 (0.11 to 0.36)	1.77 (1.34 to 2.19)	1.38 (1.06 to 1.79)	−0.22 (−0.4 to 0.06)	55.73 (38.94 to 72.2)	39.29 (29.73 to 50.16)	−0.29 (−0.49 to 0.02)
Tropical Latin America	78.45 (56.18 to 107.06)	75.9 (54.58 to 102.1)	−0.03 (−0.07 to 0.02)	13.77 (10.58 to 14.9)	8.5 (7.46 to 9.94)	−0.38 (−0.45 to −0.2)	324.25 (254.56 to 347.64)	205.66 (184.4 to 231.67)	−0.37 (−0.43 to −0.22)
Central Latin America	18.61 (13.86 to 24.63)	17.91 (13.57 to 23.35)	−0.04 (−0.08 to 0.01)	1.95 (1.73 to 2.15)	1.71 (1.4 to 2.05)	−0.12 (−0.26 to 0.05)	50.85 (45.92 to 56.74)	45.57 (36.16 to 55.03)	−0.1 (−0.28 to 0.09)
Southern Latin America	71.86 (50.17 to 99.1)	77.45 (55.96 to 106.32)	0.08 (−0.05 to 0.22)	11.39 (8.3 to 13.31)	8.32 (6.98 to 9.22)	−0.27 (−0.39 to −0.06)	243.98 (194.34 to 276.18)	169.16 (151.98 to 193.06)	−0.31 (−0.4 to −0.13)
Caribbean	26.04 (20.07 to 33.65)	27.01 (20.95 to 35.06)	0.04 (−0.02 to 0.1)	3.69 (3.05 to 4.74)	3.53 (2.83 to 4.46)	−0.04 (−0.22 to 0.13)	113.32 (88.01 to 156.54)	109.27 (80.2 to 151.1)	−0.04 (−0.25 to 0.2)
Central Europe	84.78 (62.8 to 112.35)	83.24 (63.17 to 108.88)	−0.02 (−0.11 to 0.09)	14.07 (10.14 to 15.63)	10.58 (7.47 to 12.25)	−0.25 (−0.34 to −0.16)	240.14 (193.17 to 259.92)	178.35 (131.17 to 206.35)	−0.26 (−0.36 to −0.15)
Eastern Europe	24.4 (19.09 to 30.8)	23.28 (18.05 to 29.81)	−0.05 (−0.09 to −0.01)	3.76 (2.93 to 6.44)	8.51 (4.04 to 10.48)	1.27 (−0.23 to 2.28)	106.45 (78.71 to 211.56)	265.31 (144.19 to 330.8)	1.49 (−0.19 to 2.84)
Central Asia	30.23 (22.68 to 39.71)	27.48 (20.61 to 35.97)	−0.09 (−0.15 to −0.02)	4.01 (3.34 to 5.86)	9.28 (5.31 to 11.02)	1.31 (0.05 to 2)	124.39 (103.68 to 192.55)	271.04 (160.77 to 324.8)	1.18 (−0.02 to 1.89)
North Africa and Middle East	24.77 (18.92 to 32.22)	25.28 (19.61 to 32.35)	0.02 (−0.03 to 0.07)	2.98 (2.2 to 4.4)	2.1 (1.73 to 3)	−0.29 (−0.46 to −0.1)	97.18 (67.44 to 152.22)	60.87 (48.65 to 86.39)	−0.37 (−0.55 to −0.13)
South Asia	1.14 (0.79 to 1.57)	1.19 (0.83 to 1.64)	0.05 (0.02 to 0.07)	0.27 (0.16 to 0.45)	0.26 (0.18 to 0.35)	−0.05 (−0.33 to 0.34)	5.85 (3.54 to 9.43)	5.67 (4.1 to 7.64)	−0.03 (−0.31 to 0.37)
Southeast Asia	38.07 (29.03 to 49.04)	40.88 (31.41 to 52.47)	0.07 (0.03 to 0.12)	3.6 (2.63 to 4.41)	3.36 (2.48 to 4.02)	−0.07 (−0.24 to 0.15)	81.3 (65.79 to 118.49)	74.48 (60.61 to 96.59)	−0.08 (−0.29 to 0.14)
East Asia	9.76 (7.45 to 12.5)	10.29 (7.8 to 13.16)	0.05 (0.01 to 0.1)	0.8 (0.52 to 1.52)	0.72 (0.53 to 0.94)	−0.1 (−0.54 to 0.51)	27.98 (18.33 to 53.21)	19.97 (15.5 to 24.96)	−0.29 (−0.64 to 0.2)
Oceania	20.2 (15.02 to 26.85)	20.69 (15.32 to 27.64)	0.02 (−0.05 to 0.1)	2.97 (2.02 to 4.37)	3.15 (2.05 to 4.45)	0.06 (−0.15 to 0.35)	89.18 (56.05 to 138.59)	96.31 (58.17 to 150.21)	0.08 (−0.17 to 0.4)
Western Sub-Saharan Africa	203.98 (141.74 to 277.12)	221.39 (148.49 to 307.88)	0.09 (0.05 to 0.12)	8.01 (4.58 to 10.96)	5.54 (4.03 to 6.78)	−0.31 (−0.48 to 0.04)	202.5 (121.51 to 268.91)	146.64 (110.61 to 180.76)	−0.28 (−0.44 to 0.06)
Eastern Sub-Saharan Africa	244.96 (167.9 to 333.66)	264.79 (184.77 to 357.53)	0.08 (0.04 to 0.12)	4.46 (3.08 to 5.68)	3.75 (2.6 to 4.66)	−0.16 (−0.37 to 0.11)	169.17 (115.67 to 215.48)	138.25 (97.66 to 179.08)	−0.18 (−0.4 to 0.11)
Central Sub-Saharan Africa	211.04 (137.35 to 300.5)	207.62 (134.5 to 294.55)	−0.02 (−0.08 to 0.06)	9.67 (5.21 to 14.51)	8.54 (4.96 to 13.82)	−0.12 (−0.34 to 0.23)	269.3 (147.77 to 379.34)	230.35 (141.67 to 346.6)	−0.14 (−0.37 to 0.2)
Southern Sub-Saharan Africa	262.9 (178.59 to 362.32)	240.02 (159.93 to 334.78)	−0.09 (−0.12 to −0.05)	12.42 (9.71 to 14.93)	11.27 (9.28 to 14.11)	−0.09 (−0.21 to 0.04)	294.23 (236.45 to 345.26)	252.73 (209.47 to 310.58)	−0.14 (−0.25 to −0.01)

DALY, disability-adjusted life year; UI, uncertainty interval.

**FIGURE 1:**
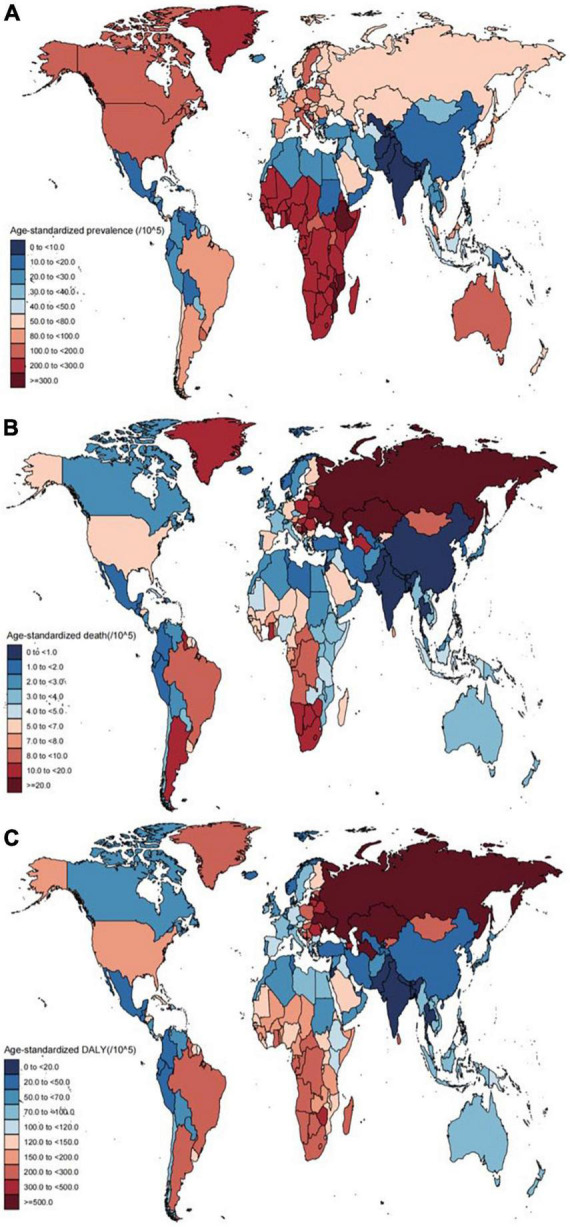
Age-standardized prevalence rates **(A)**, death **(B)**, and DALY **(C)** of cardiomyopathy for 204 countries and territories, both sexes, 2019. DALY, disability-adjusted life year.

In 2019, the highest cardiomyopathy ASRs were observed in Eastern Sub-Saharan Africa [266.89 (95% CI: 186.17–360.49) per 100,000 persons] with the highest AC and OC ASRs respectively being observed in Eastern Europe [49.54 (95% UI: 35.45–66.98) per 100,000 persons] and Eastern Sub-Saharan Africa [264.79 (95% UI: 184.77 to 357.53) per 100,000 persons] (Tables 1–3). At the national level, cardiomyopathy ASR rates varied by 233.0-fold in 2019, from a low of 1.32 (95% CI: 0.91–1.85) per 100,000 persons in Nepal to 307.15 (95% CI: 201.57–432.11) per 100,000 persons in Djibouti (Supplementary Table 1 and Figure 1).

Additionally, AC and OC ASRs varied by 907.0-fold and377.0-fold among nations, respectively, respectively ranging from0.09 (95% UI: 0.06–0.12) per 100,000 persons in Uzbekistan to 81.6 (95% UI: 59.62–109.61) per 100,000 persons in Montenegro (Supplementary Table 2 and Supplementary Figure 1), and from 0.81 (95% UI: 0.55–1.12) per 100,000 persons in Nepal to 305.28 (95% UI: 200.4–429.37) per 100,000 persons in Djibouti (Supplementary Table 3 and Supplementary Figure 2).

**FIGURE 2:**
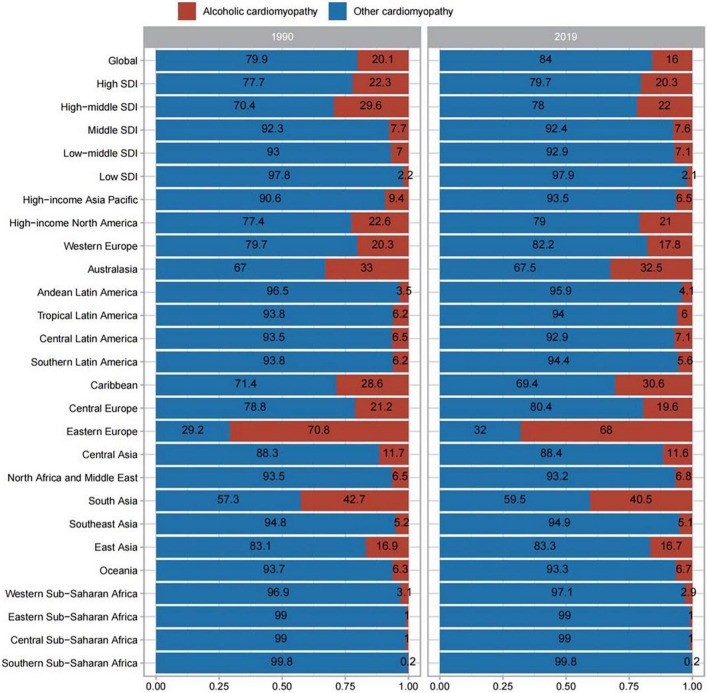
Contribution of alcoholic cardiomyopathy, and other causes to cardiomyopathy prevalence, both sexes, globally and by region, in 1990 and 2019.

The proportion of cardiomyopathy cases attributed to OC and AC at the global and regional levels in 1990 and 2019 are summarized in Figure 2. Over half of cardiomyopathy cases at the global level were caused by OC, with these proportions having largely remained stable over time with the exception of in Eastern Europe, where a significant change in this proportion was observed when comparing these time points. The proportion of AC in Eastern Europe was outnumbered that of OC in both 1990 and 2019 (70.8, 68% respectively). The gap between these two etiological forms of cardiomyopathy was also less in South Asia than in other surveyed regions.

### Cardiomyopathy-associated mortality rates in 2019

In total, the respective numbers of global deaths attributed to AC and OC were 0.07 million (95% UI: 0.06–0.08) and 0.24 million (95% UI: 0.19–0.26). The age-standardized mortality rate for cardiomyopathy in 2019 was 3.97 (95% CI: 3.29–4.39), with respective mortality rates of 0.86 (95% UI: 0.72–0.99) and 3.11 (95% UI: 2.57–3.4) for AC and OC. When comparing different SDI groups, the highest mortality rates were observed in the high-middle SDI group in all categories, followed by the high SDI group, with only slight differences among the three other groups (Tables 1–3). Of the 21 surveyed regions the age-standardized mortality rates for cardiomyopathy were highest in Eastern Europe [23.53 (95% UI: 16.33–28.42) per 100,000 persons], while for AC and OC the highest respective age-standardized mortality rates were observed in Eastern Europe [15.02 (95% UI: 12.29–17.94) per 100,000 persons] and Southern Sub-Saharan Africa [11.27 (95% UI: 9.28–14.11) per 100,000 persons] (Tables 2, 3). At the national level, age-standardized cardiomyopathy-related mortality rates varied by 96.0-fold among countries, from a low of 0.38 (95% CI: 0.27–0.52) per 100,000 persons in India to 29.18 (95% CI: 15.11–44.02) per 100,000 persons in Montenegro (Supplementary Table 1 and Figure 1). The highest age-standardized AC-related mortality rate was observed in Latvia at 16.31 (95% UI: 10.95–21.08) per 100,000 persons, while the lowest age-standardized AC-related mortality was observed in El Salvador, Ethiopia and South Sudan at 0.02[(95% UI: 0.02–0.03), (95% UI: 0.01–0.04), (95% UI: 0.01–0.05)] per 100,000 persons. The variation among them was 816.0-fold (Supplementary Table 2 and Supplementary Figure 1). Age-standardized OC-related mortality rates varied 100.0-fold among countries, from a low of 0.24 (95% UI: 0.18–0.33) per 100,000 persons in India to 24.02 (95% UI: 7.73–30.99) per 100,000 persons in Kazakhstan (Supplementary Table 3 and Supplementary Figure 2).

Proportions of cardiomyopathy-related deaths attributed to AC and OC at the global and regional levels in 1990 and 2019 are summarized in Supplementary Figure 3. At the global level, over 50% of cardiomyopathy-related deaths were attributed to OC and these proportions were stable over time, but this trend was also totally adverse in Eastern Europe. The proportion of death caused by AC both exceeded that of OC in 1990 and 2019 (75.5, 63.1% respectively). As above, the gap between these two etiologies was again found to be reduced in South Asia relative to other surveyed regions.

### Cardiomyopathy-related disability-adjusted life year rates in 2019

At the global level in 2019, 2.44 million (95% UI: 2.04–2.78) DALYs were attributed to AC, while 5.72 million (95% UI: 4.89–6.33) DALYs attributed to OC. As shown in Table 1, the age-standardized DALY rate for cardiomyopathy per 100,000 persons in 2019 was 101.95 (95% CI: 86.71–113.79), with respective rates for AC and OC of 29.2 (95% UI: 24.51–33.31) per 100,000 persons and 72.75 (95% UI: 62.2–80.48) per 100,000 persons (Tables 2, 3). When comparing different SDI groups, the largest proportion of DALYs attributed to these two forms of cardiomyopathy were observed in the high-middle SDI group, with rates of 94.53 (95% UI: 78.17–111.01) per 100,000 persons for AC and 109.27 (95% UI: 75.96–123.56) per 100,000 persons for OC (Tables 2, 3). At the regional level, the highest cardiomyopathy age-standardized DALY rates were observed in Eastern Europe [845.89 (95% CI 620.74 to 1019.77) per 100,000 persons] (Table 1). Similarly, the highest age-standardized DALY rates for AC were observed in Eastern Europe [580.58 (95% UI: 476.55–688.97) per 100,000 persons] (Table 2), whereas for OC these rates were highest in Central Asia [271.04 (95% UI: 160.77–324.8) per 100,000 persons] (Table 3). At the national level, age-standardized DALY rates varied by 44.0-fold across countries, ranging from a low of 10.34 (95% CI: 6.3–17.09) per 100,000 persons in Bhutan to a high of 858.23 (95% UI: 599.83–1107.14) per 100,000 persons in Latvia (Supplementary Table 1 and Figure 1). For AC, these rates varied 860.0-fold among surveyed nations (Supplementary Table 2 and Supplementary Figure 1), while for OC they varied 141.0-fold (Supplementary Table 3 and Supplementary Figure 2).

The proportions of cardiomyopathy-related DALYs caused by AC and OC at the global and regional levels in 1990 and 2016 are summarized in Supplementary Figure 4, revealing trends similar to those observed for prevalence and deaths.

### Time-dependent trends in cardiomyopathy burden from 1990 to 2019

From 1990 to 2019, cardiomyopathy age-standardized prevalence rates declined by −0.49% (95% CI: −0.57 to −0.41), with those for AC and OC having respectively declined by −0.32% (95% UI: −0.36 to −0.28) and −0.17% (95% UI: −0.21 to −0.13). Despite these reductions, global cardiomyopathy case numbers have risen by 41.5% over this same interval, with similar respective increases in AC and OC case numbers of 26.1 and 44.4%, respectively.

As a function of SDI quintile, the greatest drop in cardiomyopathy age-standardized prevalence rates was observed in the high-middle SDI group, followed by the high SDI group while just slightly decreases were observed in the middle, low-middle, or low SDI groups. For AC, during the time period studied. The decrease occurred in the high, high-middle and middle SDI groups, where the largest decrease was still seen in the high-middle SDI group. However, the opposite trend of increase was seen in the low-middle SDI group. In addition, no significant changes were seen in the low SDI group. Similar decreases were observed in all SDI quintiles other than the low SDI group when assessing OC age-standardized prevalence rates (Tables 1–3).

**FIGURE 3:**
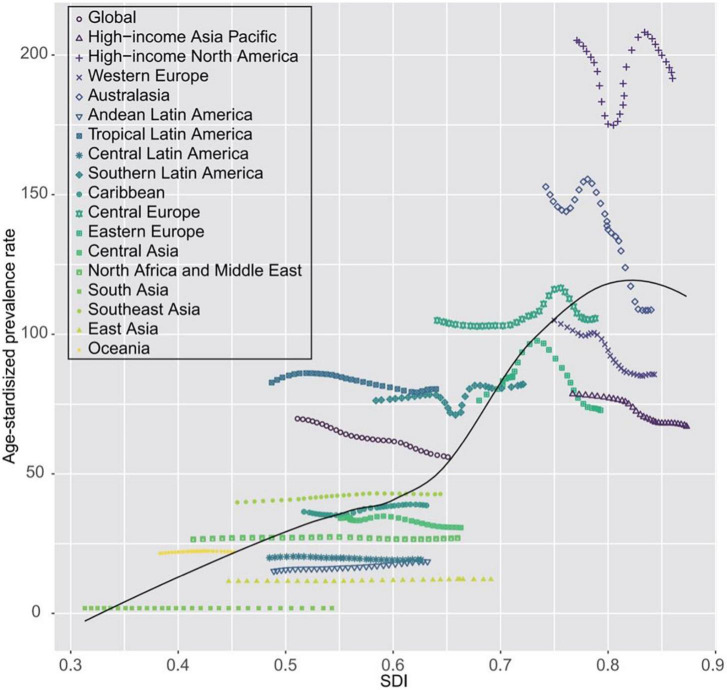
Temporal trends in age-standardized death rates of cardiomyopathy for 21 world regions by SDI, both sexes, 1990–2019. For each region, points from left to right depict estimates from each year from 1990 to 2019. SDI, socio-demographic index.

In line with the above data, age-standardized AC and OC mortality rates declined by −0.36% (95% UI: −0.5 to −0.26) and −0.39% (95% UI: −0.44 to −0.29), despite 24.8 and 30.2% increases, respectively, in the numbers of AC- and OC-related deaths during the same period. Patterns of changing age-standardized death rates across SDI quintiles were distinct from those observed for age-standardized prevalence rates (Tables 2, 3). In addition, no clear patterns in age-standardized cardiomyopathy-related mortality rates were observed across the 21 surveyed regions of the world or as a function of SDI over the 1990–2019 period (Figure 3 and Supplementary Figures 5, 6). For further details regarding time-dependent changes in AC and OC burden over the analyzed period, see Tables 1–3 and Supplementary Tables 1–3.

## Discussion

Herein, we leveraged the GBD 2019 modeling framework to estimate the global burden of AC and OC, with data being stratified by year, SDI, and geographic region. While one recent report discussed the prevalence rates, mortality rates, YLDs, and YLLs associated with both AC and OC in 2017 and national, regional, and global levels, it did not analyze associated DALY-related data. The present study is the first to our knowledge to have systematically assessed the burden of AC and OC as of 2019 at the national, regional, and global levels. Through these analyses, we determined that there were approximately 0.71 million and 3.73 million worldwide AC and OC cases, respectively, in 2019. We further determined that there were 2.44 million and 5.72 million DALYs attributable to AC and OC, respectively, in 2019 at the global level. Together these results provide an epidemiological foundation that can guide public health efforts and policymakers as they seek to better understand, prevent, detect, and treat cardiomyopathy in a manner that better optimizes health system resource allocation.

We additionally evaluated differences in global AC and OC prevalence as a function of SDI and region. At all time points, AC prevalence were higher in regions with high SDI values irrespective of sex, while OC prevalence rates were higher in areas with low SDI values. The greatest decreases in overall cardiomyopathy prevalence were observed in high-middle SDI regions, while the largest increase in AC prevalence was observed in low-middle SDI regions, and no increasing trends were observed for OC. The primary components of SDI include gross fertility rates, per capita income levels, and years of education. As such, social factors can influence cardiomyopathy prevalence rates and associated changes. One possible explanation for this may be that healthcare surveillance systems in high SDI regions are more comprehensive, while there is significant room for improving healthcare systems in low, low-middle, and middle SDI regions in order to improve the prevention and diagnosis of both AC and OC, particularly among individuals with asymptomatic disease.

We found that cardiomyopathy burden in 2019 varied substantially among geographic regions, with the highest age-standardized rates of overall cardiomyopathy and OC being evident in Eastern Sub-Saharan Africa, likely owing to the more haphazard medical conditions in many African nations and a consequent lack of surveillance and detection systems. In addition, rates of communicable diseases and peripartum cardiomyopathy are particularly high in Africa (18, 19). The highest age-standardized AC prevalence rates were observed in Eastern Europe, potentially due to high levels of alcohol intake and types of alcoholic beverage consumed in this region (20). Alcoholism and associated issues have been reported to impose a major burden on public health in Eastern Europe (21). While many nations in this region have enacted policies aimed at curbing alcohol intake resulting in lower rates of alcohol-associated mortality, rates of associated morbidity nonetheless remain relatively high (22).

The analyses conducted herein revealed that Eastern Europe exhibited the highest age-standardized prevalence of AC as well as the highest age-standardized cardiomyopathy-related mortality rate. This emphasizes the importance of alcohol intake as a major risk factor associated with global disease burden that results in substantial health-related losses (23). Higher frequencies of alcohol use likely coincide with higher AC prevalence. Given the importance of alcohol intake as a driver of AC development, further studies of national trends pertaining to alcohol consumption and associated AC surveillance are warranted to guide the design of targeted prevention strategies.

Age-standardized DALYs were used to quantify the overall burden of cardiomyopathy, which decreased over the study period. However, owing to the overall growth and aging of the global population, a larger number of DALYs were lost due to cardiomyopathy over time during the analyzed period. Moreover, higher age-standardized DALYs were observed in SDI regions exhibiting higher prevalence and mortality rates. At the regional level, DALYs associated with cardiomyopathy declined in most areas over the study period. In contrast, however, these DALYs rose Eastern Europe and Central Asia over this duration. This may be attributable to alcohol intake in these regions, to the effective treatment of complications such as heart failure, or to changes in rates of successful heart transplantation, all of which vary unequally as a function of socioeconomic status (24).

With respect to time-dependent trends in disease burden observed from 1990 to 2019, the overall global prevalence of AC and OC have significantly risen, as have overall mortality rates associated with these two forms of cardiomyopathy. Conversely, the age-standardized prevalence and mortality rates for these conditions have declined over this same interval. While the precise basis for these trends remains unclear, it may be in part due to the growth and aging of the global population (25). We did not observe any clear patterns in age-standardized cardiomyopathy-related mortality rates across the 21 surveyed regions of the world over this 30-year period, suggesting that any such relationships that may exist are complex and non-linear. Consistent with this observation, the burden of AC and OC was not restricted to nations with particular levels of socioeconomic development, instead being highly prevalent in nations with a range of SDI values.

### Limitations

While these GBD-based epidemiological estimates provide insight regarding the global burden of AC and OC that would otherwise be difficult or impossible to quantify, there are nonetheless certain limitations to this analysis. For one, data availability for certain countries was limited. While appropriate statistical efforts were employed to account for such data scarcity and associated uncertainty, the resultant data are primarily based upon trends in neighboring countries and/or covariates related to these diseases. Secondly, while the GBD study seeks to achieve maximal reliability and comparability with respect to the included data, there are inevitable instances of delayed/inaccurate reporting, variable data collection/source quality, misclassification, and coding deviations among countries. Lastly, the diagnostic criteria for specific conditions can change over time and be reflective of regional coding trends, with varied nomenclature and classifications for these diseases that can be vague or contradictory having been reported in the literature (26).

## Conclusion

Our results suggest that the cardiomyopathy burden across the world increased substantially from 1990 to 2019, and also remains an important global public health problem with the increasing numbers of prevalent cases, deaths, DALYs over the past decades. There are significant geographic variations in the burden of cardiomyopathy, suggesting that efforts to implement systematic surveillance systems should be implemented, particularly in middle- and low-income regions. Therefore, efforts to better manage this disease, as well as associated risk factors and complications. Public health policymakers and other officials should seek to develop regionally-appropriate efforts with the goal of counteracting and mitigating the future burden of cardiomyopathy. Further research is warranted to expand our knowledge of potential risk factors and to improve the prevention, early detection, and treatment of cardiomyopathy.

## Data availability statement

The original contributions presented in this study are included in the article/Supplementary material, further inquiries can be directed to the corresponding author.

## Ethics statement

The authors are accountable for all aspects of the work in ensuring that questions related to the accuracy or integrity of any part of the work are appropriately investigated and resolved. This study was conducted in accordance with the Declaration of Helsinki (as revised in 2013). All information from the GBD program is available and free for public, so the agreement of the medical ethics committee board was not necessary.

## Author contributions

SC analyzed the data, drafted, and revised the manuscript. LJ, YH, and ZL prepared the data. JG conceived and designed the study, interpreted the results, drafted, and revised the manuscript. All authors provided critical comments on the manuscript, read, and approved the final manuscript.
