# Supplementary material for: National, regional, and global cardiomyopathy burden from 1990 to 2019
Source: Front Cardiovasc Med. 2022 Nov 30;9:1042448. doi: 10.3389/fcvm.2022.1042448 (PMC9748073; doi:10.3389/fcvm.2022.1042448)
Supplement: Supplementary file 1 [file Data_Sheet_1.pdf]

Supplementary Materials

Supplementary Figure captions

Supplementary Figure 1: Age-standardized prevalence rates (A), death (B), and DALY (C) of alcoholic cardiomyopathy for 204 countries and territories, both sexes, 2019. DALY: Disability-Adjusted Life Year.

Supplementary Figure 2: Age-standardized prevalence rates (A), death (B), and DALY (C) of other cardiomyopathy for 204 countries and territories, both sexes, 2019. DALY: Disability-Adjusted Life Year.

Supplementary Figure 3: Contribution of alcoholic cardiomyopathy, and other causes to cardiomyopathy deaths, both sexes, globally and by region, in 1990 and 2019.

Supplementary Figure 4: Contribution of alcoholic cardiomyopathy, and other causes to cardiomyopathy DALYs, both sexes, globally and by region, in 1990 and 2019. DALY: Disability-Adjusted Life Year.

Supplementary Figure 5: Temporal trends in age-standardized death rates of alcoholic cardiomyopathy for 21 world regions by SDI, both sexes, 1990–2019. For each region, points from left to right depict estimates from each year from 1990 to 2019. SDI, Socio-demographic Index.

Supplementary Figure 6: Temporal trends in age-standardized death rates of other cardiomyopathy for 21 world regions by SDI, both sexes, 1990–2019. For each region, points from left to right depict estimates from each year from 1990 to 2019. SDI, Socio-demographic Index.

Table S1. Age-standardized prevalence, death, DALYs rates of the cardiomyopathy, and their percentage changes with 95% CIs from 1990 to 2019 at national level, both sexs.

Countries	Prevalence			Deaths			DALYs		
	1990 age-standardized rate per 100,000 people	2019 age-standardized rate per 100,000 people	Percentage change in age-standardized rates, 1990–2019	1990 age-standardized rate per 100,000 people	2019 age-standardized rate per 100,000 people	Percentage change in age-standardized rates, 1990–2019	1990 age-standardized rate per 100,000 people	2019 age-standardized rate per 100,000 people	Percentage change in age-standardized rates, 1990–2019
Afghanistan	8.92 (6.69 to 11.59)	9.46 (7.17 to 12.06)	0.15 (-0.06 to 0.39)	3.18 (1.34 to 6.49)	2.5 (1.2 to 4.6)	-0.63 (-1.04 to 0.2)	80.75 (32.65 to 183.24)	59.54 (28.67 to 116.53)	-0.7 (-1.11 to 0.16)
Albania	77.15 (55.66 to 105.38)	64.77 (46.8 to 89.68)	-0.32 (-0.51 to -0.07)	12.1 (7.79 to 14.58)	6.53 (4.42 to 10.57)	-0.93 (-1.31 to -0.07)	211.16 (151.01 to 252.3)	129.56 (87.71 to 215.07)	-0.78 (-1.18 to 0)
Algeria	20.95 (15.37 to 27.85)	21.68 (16.08 to 28.63)	0.07 (-0.12 to 0.29)	3.27 (2.28 to 5.15)	2.24 (1.59 to 3.38)	-0.84 (-1.29 to -0.09)	85.18 (57.4 to 152.77)	53.41 (37.5 to 82.5)	-0.86 (-1.31 to -0.08)
American Samoa	88.86 (66.04 to 117.51)	89.58 (66.02 to 118.09)	0.01 (-0.2 to 0.28)	8.2 (6.5 to 11.41)	7.19 (5.55 to 9.5)	-0.82 (-1.12 to -0.19)	233.35 (186.11 to 319.9)	212.13 (161.2 to 283.4)	-0.77 (-1.1 to -0.11)
Andorra	116.34 (87.36 to 150)	84.37 (63.29 to 109.64)	-0.55 (-0.71 to -0.37)	6.74 (3.97 to 9.92)	5.57 (3.71 to 7.42)	-0.5 (-1.03 to 0.31)	132.05 (88.36 to 190.77)	101.38 (72.87 to 134.61)	-0.53 (-1.04 to 0.24)
Angola	200.7 (130.12 to 288.52)	220.99 (143.68 to 312.09)	0.18 (-0.06 to 0.45)	8.6 (5.03 to 12.43)	9.66 (4.91 to 14.7)	-0.47 (-1.03 to 0.61)	278.64 (141.32 to 404.3)	236.05 (147.78 to 323.9)	-0.48 (-1.05 to 0.64)
Antigua and Barbuda	32.58 (25.32 to 41.45)	34.07 (25.5 to 44.85)	0.08 (-0.29 to 0.47)	4.19 (3.64 to 5.24)	5.11 (3.54 to 6.26)	1.06 (-0.3 to 2.57)	109.13 (95.21 to 137.69)	121.37 (85.58 to 148.93)	0.88 (-0.39 to 2.29)
Argentina	80.14 (56.4 to 110.91)	63.92 (43.09 to 89.47)	1.33 (0.6 to 2.32)	14.85 (10.34 to 17.75)	10.99 (8.67 to 12.29)	-1.07 (-1.24 to -0.8)	326.71 (256.74 to 380.1)	217.41 (190.35 to 244.65)	-1.12 (-1.27 to -0.88)
Armenia	41.98 (31.49 to 54.74)	21.1 (15.77 to 27.79)	-1 (-1.11 to -0.86)	6.77 (4.16 to 9.72)	3.91 (2.24 to 5.6)	-0.01 (-1.14 to 1.82)	150.68 (116.37 to 199.94)	78.75 (51.8 to 114.67)	-0.14 (-1.13 to 1.15)

Australia	128.9 (96.66 to 168.5)	150.82 (114.06 to 199.02)	-0.58 (-0.73 to -0.42)	7.18 (4.69 to 8.31)	3.22 (2.59 to 4.2)	-1.01 (-1.31 to -0.5)	175.48 (133.67 to 198.25)	83.7 (68.09 to 110.12)	-0.98 (-1.25 to -0.59)
Austria	194.4 (145.48 to 248.93)	133.37 (101.34 to 169.2)	-0.59 (-0.78 to -0.35)	35.94 (7.82 to 43.54)	7.83 (5.36 to 9.34)	0.7 (-1.21 to 2.57)	457.19 (157.37 to 540.08)	112.45 (93.2 to 144.31)	0.37 (-1.14 to 1.77)
Azerbaijan	57.13 (43.05 to 73.89)	52.9 (39.58 to 68.47)	-0.11 (-0.31 to 0.1)	14.58 (11.27 to 20.73)	16.06 (12.14 to 23.37)	0.02 (-0.55 to 0.84)	417.48 (306.58 to 640.44)	408.95 (293.05 to 679.02)	-0.17 (-0.7 to 0.58)
Bahamas	40.67 (30.81 to 53.35)	41.3 (31.32 to 54.31)	-0.04 (-0.26 to 0.22)	9.07 (7.6 to 11.07)	9.42 (7.43 to 11.74)	0.21 (-0.4 to 1.04)	264.55 (221.58 to 325.51)	270.58 (207.37 to 343.63)	0.19 (-0.41 to 1.01)
Bahrain	79.6 (59.76 to 104.53)	82.02 (62.14 to 108.35)	0.05 (-0.17 to 0.3)	5.69 (4.34 to 8.27)	4.4 (3.16 to 5.9)	-0.63 (-1.18 to 0.17)	148.05 (118.98 to 206.71)	98.27 (72.68 to 133.37)	-0.77 (-1.25 to -0.09)
Bangladesh	1.95 (1.36 to 2.7)	2.07 (1.46 to 2.82)	0.11 (-0.12 to 0.37)	0.49 (0.24 to 0.89)	0.47 (0.26 to 0.75)	-0.15 (-1 to 1.52)	12.12 (5.86 to 20.93)	11.08 (6 to 17.63)	-0.2 (-1.09 to 1.52)
Barbados	68.26 (52.19 to 88.04)	72.57 (54.78 to 95.36)	0.15 (-0.09 to 0.42)	6.39 (5.42 to 7.75)	6.84 (5.34 to 8.43)	0.28 (-0.35 to 1.05)	174.72 (149.68 to 209.14)	173.58 (134.41 to 216.75)	0.12 (-0.47 to 0.83)
Belarus	44.7 (32.75 to 60.33)	56.9 (42.07 to 76.67)	0.54 (0.28 to 0.83)	9.5 (6.21 to 13.01)	10.24 (6.76 to 14.85)	-0.08 (-0.73 to 0.89)	333.2 (209.15 to 463.79)	395.02 (259.32 to 554.24)	0.11 (-0.59 to 1.09)
Belgium	88.45 (69.38 to 109.85)	100.49 (75.49 to 128.99)	-0.22 (-0.56 to 0.2)	7.4 (4.25 to 8.71)	4.03 (3.07 to 4.8)	-0.77 (-1.12 to -0.28)	128.53 (89.85 to 145.41)	68.4 (57.11 to 85.6)	-0.75 (-1.08 to -0.27)
Belize	52.53 (39.63 to 68.59)	49.01 (37.34 to 65.46)	0.18 (-0.07 to 0.45)	5.38 (3.88 to 7.04)	6.09 (4.71 to 7.17)	0.85 (-0.19 to 2.73)	155.4 (116.57 to 195.97)	166.28 (129.64 to 196.19)	0.77 (-0.23 to 2.62)
Benin	210.41 (136.31 to 301.21)	218.12 (142.35 to 311.08)	0.1 (-0.12 to 0.35)	8.51 (4.54 to 12.37)	5.53 (3.91 to 7.36)	-0.97 (-1.32 to -0.27)	225.98 (127.6 to 316.3)	154.58 (111.9 to 209.61)	-0.91 (-1.28 to -0.22)
Bermuda	62.75 (48.01 to 82.15)	66.1 (50.58 to 85.69)	-0.1 (-0.29 to 0.11)	6.13 (4.79 to 8.39)	5.17 (3.62 to 6.45)	-0.05 (-0.99 to 1.31)	147.87 (116.26 to 204.62)	116.74 (85.24 to 145.56)	-0.12 (-1.03 to 1.15)
Bhutan	1.52 (1.03 to 2.1)	1.72 (1.18 to 2.42)	0.26 (0.01 to 0.55)	0.54 (0.28 to 0.96)	0.38 (0.19 to 0.71)	0.11 (-0.74 to 2.13)	10.26 (4.23 to 19.81)	10.34 (6.3 to 17.09)	0.01 (-0.86 to 2.01)

Bolivia (Plurinational State of)	5.52 (3.24 to 9.19)	10.43 (6.95 to 15.25)	2.48 (1.25 to 5.16)	2.68 (1.65 to 4.04)	2.43 (1.61 to 3.27)	-0.59 (-1.1 to 0.33)	82.67 (46.1 to 129.74)	62.74 (40.97 to 84.53)	-0.76 (-1.24 to 0.13)
Bosnia and Herzegovina	132.39 (95.55 to 181.54)	104.02 (73.63 to 142.29)	-0.4 (-0.6 to -0.2)	13.71 (7.69 to 20.57)	15.22 (7.91 to 21.96)	-0.07 (-0.73 to 0.82)	246.4 (158.64 to 349.23)	262.61 (139.84 to 386.74)	-0.04 (-0.68 to 0.82)
Botswana	236.98 (151.96 to 336.47)	239.89 (156.06 to 339.67)	0.01 (-0.19 to 0.21)	11.84 (8.47 to 17.54)	15.72 (12.09 to 21.24)	-0.59 (-1.07 to 0.15)	349.86 (271.29 to 474.44)	274.99 (197.69 to 402.56)	-0.51 (-1.03 to 0.22)
Brazil	84.16 (60 to 115.25)	81.4 (58.43 to 110.09)	0.01 (-0.09 to 0.15)	15.66 (12.06 to 17.26)	9.13 (7.96 to 10.72)	-1.08 (-1.21 to -0.82)	386.76 (303.57 to 425.52)	227.33 (201.42 to 257.86)	-1.05 (-1.17 to -0.83)
Brunei Darussalam	58.46 (42.79 to 78.18)	67.9 (50.34 to 89.84)	-0.37 (-0.54 to -0.19)	10.01 (7.45 to 15.09)	8.99 (6.98 to 10.96)	-0.58 (-1.04 to -0.02)	276.86 (210.78 to 415.1)	221.76 (179.17 to 273.35)	-0.69 (-1.11 to -0.2)
Bulgaria	18.7 (13.24 to 25.85)	15.93 (11.18 to 22.12)	-0.28 (-0.5 to -0.01)	2.34 (1.17 to 7.6)	5.19 (4.01 to 6.86)	0.88 (-0.85 to 3.7)	57.26 (35.67 to 145.5)	114.77 (87.51 to 146.61)	0.72 (-0.86 to 2.81)
Burkina Faso	203.97 (134.53 to 288.95)	200.95 (129.49 to 285.46)	-0.04 (-0.25 to 0.21)	9.26 (4.79 to 14.92)	7.41 (5.23 to 10.17)	-0.75 (-1.19 to 0.04)	236.48 (125.32 to 367.9)	192.74 (138.01 to 262.48)	-0.73 (-1.18 to 0.06)
Burundi	266.94 (177.54 to 373.18)	273.44 (177.16 to 389.82)	0.01 (-0.19 to 0.24)	3.86 (2.59 to 5.4)	5.23 (3.28 to 7.77)	-0.93 (-1.41 to 0.03)	201.64 (122.2 to 298.95)	140.77 (99.53 to 192.33)	-0.91 (-1.39 to 0.03)
Cabo Verde	267.25 (176.32 to 377.47)	248.54 (159.37 to 352.56)	0.18 (-0.05 to 0.42)	2.28 (1.71 to 2.94)	3.07 (2.34 to 3.99)	0.54 (-0.26 to 1.8)	81.53 (63.27 to 103.62)	92.29 (73.33 to 117.36)	0.26 (-0.37 to 1.14)
Cambodia	26 (19.04 to 34.8)	27.72 (20.27 to 36.64)	0.16 (-0.09 to 0.44)	3.39 (2.52 to 4.67)	3.18 (2.11 to 4.78)	-0.15 (-0.78 to 1.06)	79.86 (49.76 to 147.62)	73.72 (55.27 to 110.44)	-0.29 (-0.95 to 0.96)
Cameroon	204.53 (129.52 to 296.4)	174.52 (121.88 to 243.22)	0.45 (-0.12 to 1.09)	8.51 (4.43 to 12.07)	5.86 (4.06 to 8.17)	-0.84 (-1.29 to 0.03)	212.96 (114.1 to 298.85)	160.25 (112.15 to 224.14)	-0.75 (-1.23 to 0.18)
Canada	285.36 (214.63 to 372.88)	143.24 (114.89 to 177.73)	-1 (-1.14 to -0.84)	3.52 (3 to 4.18)	2.31 (1.85 to 3.18)	-0.64 (-0.96 to -0.24)	115.9 (98.88 to 132.58)	68.67 (57.9 to 88.43)	-0.77 (-0.99 to -0.47)
Central African Republic	176.3 (114.82 to 251.31)	191.68 (123.96 to 275.72)	-0.16 (-0.37 to 0.05)	11.7 (5.71 to 19.42)	9.97 (5.09 to 16.04)	-0.44 (-0.95 to 0.37)	322.48 (155.38 to 506.84)	273.33 (139.13 to 412.69)	-0.44 (-0.93 to 0.3)

Chad	193.79 (125.77 to 278.18)	205.56 (130.36 to 292.93)	0.19 (-0.04 to 0.47)	8.18 (4.18 to 13.23)	6.02 (4.24 to 8.17)	-0.79 (-1.24 to 0.07)	211.66 (113.58 to 320.39)	163.23 (115.98 to 224.58)	-0.73 (-1.19 to 0.11)
Chile	94.13 (65.86 to 131.6)	76.95 (55.25 to 103.7)	-0.27 (-0.53 to 0.04)	5.16 (4.37 to 7.28)	3.64 (3.12 to 4.79)	-0.94 (-1.13 to -0.69)	135.87 (116.68 to 175.35)	93.27 (81.99 to 112.89)	-0.92 (-1.14 to -0.68)
China	10.67 (8.07 to 13.83)	11.47 (8.62 to 14.8)	0.17 (0.09 to 0.25)	0.86 (0.52 to 1.73)	0.83 (0.59 to 1.1)	0.17 (-1.08 to 2.42)	30.4 (19.13 to 59.35)	23.96 (17.8 to 30.47)	0.06 (-1.08 to 1.87)
Colombia	29.12 (21.7 to 39.37)	25.87 (19.4 to 34.59)	-0.2 (-0.39 to 0.02)	2.28 (1.69 to 2.93)	2.63 (2.31 to 2.99)	-1.19 (-1.39 to -0.9)	80.7 (70.47 to 92.24)	49.61 (37.27 to 67.76)	-1.2 (-1.41 to -0.89)
Comoros	288.48 (192.29 to 403.35)	279.02 (185.97 to 396.04)	0.06 (-0.17 to 0.3)	4.42 (2.5 to 6.33)	3.38 (2.12 to 4.7)	-0.65 (-1.22 to 0.78)	170.7 (95.83 to 246.91)	127.77 (82.28 to 172.19)	-0.61 (-1.16 to 0.86)
Congo	236.27 (154.6 to 335.87)	237.15 (152.89 to 335.85)	0.03 (-0.19 to 0.27)	11.03 (5.9 to 15.6)	8.33 (4.82 to 11.97)	-0.75 (-1.24 to 0.07)	299.99 (156.77 to 410.2)	223.97 (137.13 to 313.52)	-0.76 (-1.26 to 0.15)
Cook Islands	9.25 (6.85 to 12.22)	9.2 (6.82 to 12.24)	-0.01 (-0.22 to 0.2)	1.31 (0.94 to 1.75)	0.78 (0.58 to 1.03)	-0.97 (-1.26 to -0.52)	39.12 (27.75 to 54.01)	21.8 (15.72 to 28.72)	-0.97 (-1.29 to -0.45)
Costa Rica	49.19 (37.16 to 64.58)	50.52 (37.48 to 66.8)	0 (-0.25 to 0.31)	5.3 (4.47 to 6.16)	3.91 (2.88 to 5.56)	-0.68 (-1.08 to -0.14)	136.12 (119.08 to 155.22)	99.84 (74.79 to 134.14)	-0.66 (-1.04 to -0.14)
Croatia	54.78 (38.9 to 75.08)	37.73 (27.86 to 50.15)	-0.61 (-0.92 to -0.18)	7.18 (5.65 to 10.14)	6.81 (4.88 to 9.35)	0.42 (-0.32 to 1.26)	121.58 (100.3 to 186.27)	113.95 (80.04 to 165.98)	0.5 (-0.38 to 1.44)
Cuba	40.47 (30.16 to 53.89)	49.65 (36.68 to 66.78)	0.47 (0.16 to 0.81)	2.7 (2.16 to 4.47)	4.92 (3.69 to 6.17)	2.24 (0.62 to 3.78)	86.54 (70.6 to 127.32)	133.29 (101.76 to 166.69)	1.57 (0.37 to 2.84)
Cyprus	53.95 (42 to 68.25)	62.98 (47.75 to 82.01)	-0.25 (-0.52 to 0.11)	6.88 (4.26 to 10.52)	4.35 (3.4 to 6.16)	-0.93 (-1.25 to -0.37)	141.67 (92.73 to 182.99)	82.58 (64 to 102.3)	-0.97 (-1.25 to -0.48)
Czechia	48.24 (35.4 to 65.06)	53.31 (38.53 to 74.12)	0.24 (-0.13 to 0.78)	2.79 (2.19 to 3.95)	4.19 (3.47 to 7.27)	4.19 (0.56 to 7.62)	114.34 (94.97 to 139.83)	87.44 (62.2 to 111.21)	3.71 (0.66 to 6.61)
Cote d'Ivoire	237.33 (155.47 to 338.27)	238.49 (153.24 to 341.65)	0.01 (-0.21 to 0.25)	8.21 (4.25 to 11.63)	5.17 (3.61 to 6.97)	-1.01 (-1.35 to -0.34)	213.68 (114.77 to 295.06)	143.79 (101.33 to 189.47)	-0.94 (-1.3 to -0.25)

Democratic People's Republic of Korea	13.69 (10.34 to 18.07)	13.26 (9.93 to 17.35)	-0.04 (-0.24 to 0.16)	1.76 (1.12 to 2.9)	1.58 (1.06 to 2.32)	-0.11 (-0.74 to 0.89)	54.96 (33.89 to 95.96)	44.41 (30.36 to 64.94)	-0.18 (-0.84 to 0.86)
Democratic Republic of the Congo	204.56 (130 to 293.41)	214.68 (138.99 to 310.33)	-0.08 (-0.27 to 0.13)	9.58 (5.27 to 14.92)	8.62 (4.9 to 15.04)	-0.41 (-0.99 to 0.55)	265.72 (147.83 to 389.38)	231.02 (139.45 to 380.07)	-0.42 (-0.98 to 0.52)
Denmark	26.53 (20.02 to 34.11)	31.11 (23.86 to 39.58)	-0.61 (-0.74 to -0.46)	2.38 (1.84 to 4.34)	1.9 (1.47 to 3.28)	-0.52 (-0.8 to -0.18)	67.86 (55.86 to 93.28)	46.34 (38.29 to 60.93)	-0.69 (-0.94 to -0.37)
Djibouti	275.39 (178.85 to 389.54)	307.15 (201.57 to 432.11)	0.25 (0.03 to 0.51)	4.71 (2.77 to 6.71)	4.02 (2.19 to 6.09)	-0.43 (-1.04 to 0.64)	177.76 (109.65 to 255.11)	151.84 (90.93 to 234.95)	-0.38 (-0.95 to 0.68)
Dominica	85.3 (64.43 to 110.92)	83.88 (64.2 to 108.88)	0.09 (-0.16 to 0.36)	21.62 (16.01 to 27.49)	19.74 (15.87 to 27.04)	0.56 (-0.32 to 1.98)	490.23 (395.27 to 666.59)	538.99 (392.59 to 697.21)	0.5 (-0.35 to 1.9)
Dominican Republic	17.44 (13.21 to 23.12)	19.82 (14.92 to 26.64)	0.27 (0.02 to 0.56)	1.97 (1.54 to 2.79)	2.64 (1.62 to 3.58)	1.23 (-0.12 to 3.21)	62.44 (49.53 to 86.73)	73.63 (45.98 to 100.92)	1.01 (-0.27 to 2.87)
Ecuador	12.89 (9.04 to 17.86)	15.35 (11.31 to 20.48)	0.46 (0.16 to 0.88)	1.78 (1.32 to 2.33)	1.52 (1.19 to 2.3)	-0.33 (-0.91 to 0.52)	43.08 (33.89 to 64.77)	45.41 (34.21 to 59.03)	-0.4 (-0.93 to 0.4)
Egypt	28.71 (19.36 to 41.35)	24.92 (17 to 36.45)	0.34 (-0.06 to 0.77)	3.86 (2.32 to 6.44)	2.71 (1.79 to 4.26)	-0.7 (-1.22 to 0.11)	166.95 (89.43 to 316.43)	93.87 (60.71 to 148.26)	-0.78 (-1.32 to 0.16)
El Salvador	7.22 (5.09 to 9.95)	8.44 (6.07 to 11.35)	0.37 (0.12 to 0.7)	0.89 (0.64 to 1.08)	0.7 (0.49 to 0.96)	-0.76 (-1.15 to -0.15)	26.9 (19.11 to 33.56)	19.39 (13.31 to 26.29)	-0.8 (-1.2 to -0.21)
Equatorial Guinea	198.85 (127.01 to 281.06)	230.68 (151.06 to 327.52)	0.3 (0.03 to 0.6)	10.37 (4.89 to 17.26)	7.02 (3.36 to 11.26)	-1.01 (-1.53 to 0.14)	289.53 (132.69 to 456.87)	184.22 (93.5 to 295.65)	-1.03 (-1.53 to 0.13)
Eritrea	201.36 (131.5 to 289.3)	223.77 (146.95 to 323.01)	0.22 (0 to 0.49)	4.46 (2.66 to 6.58)	3.88 (2.24 to 5.44)	-0.56 (-1.08 to 0.32)	164.23 (95.53 to 241.62)	138.32 (87.21 to 195.62)	-0.54 (-1.06 to 0.34)
Estonia	63.49 (45.54 to 87.58)	74.67 (52.89 to 103.77)	0.37 (0.09 to 0.69)	12.37 (7.49 to 15.92)	9.28 (6.3 to 12.25)	-0.31 (-0.87 to 0.53)	443.38 (268.49 to 570.16)	298.94 (207.57 to 393.42)	-0.48 (-0.98 to 0.34)
Eswatini	223.26 (143.18 to 319.88)	214.75 (136.19 to 309.77)	-0.08 (-0.28 to 0.13)	14.87 (11.19 to 20.06)	11.9 (8.21 to 18.25)	-0.48 (-0.98 to 0.27)	332.93 (254.31 to 438.2)	271.36 (190.66 to 401.76)	-0.41 (-0.94 to 0.41)

Ethiopia	263.61 (193.12 to 340.73)	306.75 (226.82 to 394.23)	0.31 (0.19 to 0.44)	4.32 (2.55 to 6.69)	3.1 (2.07 to 4.02)	-0.84 (-1.4 to 0.46)	168.71 (101.37 to 255.7)	116.73 (84.19 to 148.08)	-0.81 (-1.37 to 0.36)
Fiji	23.98 (17.67 to 31.72)	23.82 (17.79 to 31.69)	0 (-0.21 to 0.24)	3.42 (2.7 to 4.65)	3.36 (2.41 to 4.33)	-0.36 (-0.86 to 0.38)	102.02 (79.96 to 137.15)	105.82 (75.6 to 139.01)	-0.28 (-0.8 to 0.54)
Finland	83.11 (63.65 to 105.46)	74.19 (57.37 to 93.64)	-0.22 (-0.47 to 0.07)	6.19 (4.22 to 11.16)	5.37 (4.17 to 7.45)	-0.17 (-0.81 to 0.61)	189.73 (130.17 to 275.52)	143.87 (110.34 to 164.92)	-0.35 (-0.9 to 0.27)
France	173.79 (134.66 to 219.87)	92.48 (71.58 to 116.53)	-0.93 (-1.05 to -0.8)	7.31 (5.39 to 8.31)	3.78 (3.01 to 4.69)	-1.08 (-1.32 to -0.76)	148.14 (121.46 to 163.84)	72.15 (59.67 to 94.97)	-1.08 (-1.31 to -0.77)
Gabon	235.2 (154.49 to 337.23)	236.04 (152.52 to 334.43)	0.01 (-0.18 to 0.22)	10.02 (5 to 14.78)	7.9 (4.22 to 11.08)	-0.71 (-1.22 to 0.16)	276.71 (141.7 to 395.49)	213.67 (119.1 to 299.18)	-0.71 (-1.2 to 0.15)
Gambia	261.9 (171.3 to 366.67)	249.55 (161.88 to 356.49)	0.03 (-0.19 to 0.27)	7.56 (4.11 to 11.56)	5.7 (4.07 to 7.75)	-0.81 (-1.27 to 0.06)	196.98 (112.66 to 290.01)	155.9 (114.64 to 208.71)	-0.75 (-1.22 to 0.12)
Georgia	15.98 (11.2 to 21.91)	17.29 (12.96 to 22.69)	-0.08 (-0.33 to 0.16)	3.34 (2.45 to 4.16)	3.97 (2.54 to 5.68)	0.61 (-0.3 to 3.23)	87.52 (67.67 to 110.59)	112.53 (77.18 to 163.66)	1.05 (-0.11 to 3.76)
Germany	89.66 (69.29 to 115.59)	95.92 (73.18 to 122.81)	-0.3 (-0.51 to -0.05)	9.7 (7.31 to 12.03)	5.44 (4.51 to 6.53)	-0.9 (-1.15 to -0.56)	212.05 (162.03 to 259.56)	110.23 (93.65 to 129.54)	-0.95 (-1.18 to -0.64)
Ghana	201.77 (129.04 to 293.69)	181.08 (125.06 to 257.86)	0.23 (-0.31 to 0.92)	8.54 (6.46 to 12.23)	10.12 (6.56 to 13.27)	0.16 (-0.73 to 1.5)	212.22 (159.64 to 294.55)	248.68 (169 to 330.82)	0.17 (-0.68 to 1.47)
Greece	58.17 (44.85 to 73.83)	43.89 (33.39 to 56.24)	-0.47 (-0.61 to -0.32)	6.07 (3.42 to 7.02)	3.47 (2.66 to 3.98)	-0.95 (-1.17 to -0.49)	100.15 (66.61 to 112.49)	71.46 (49.36 to 82.37)	-0.77 (-1.04 to -0.41)
Greenland	277.12 (215.43 to 350.97)	209.54 (162.79 to 268.62)	-0.48 (-0.64 to -0.3)	13.55 (10.05 to 16.89)	11 (7.74 to 14.16)	-0.33 (-0.88 to 0.45)	418.72 (319.43 to 517.05)	287.9 (204.7 to 378.83)	-0.53 (-0.99 to 0.18)
Grenada	56.8 (42.75 to 74.15)	57.15 (42.56 to 75.21)	0.13 (-0.11 to 0.37)	10.3 (8.77 to 12.4)	10.57 (7.93 to 12.38)	0.3 (-0.6 to 1.23)	275.58 (233.47 to 335.16)	263.32 (196.31 to 311.29)	0.17 (-0.67 to 1.06)
Guam	37.17 (27.48 to 49.56)	38.93 (28.9 to 51.99)	0.1 (-0.14 to 0.36)	4.42 (3.4 to 5.96)	2.48 (1.87 to 3.78)	-1.19 (-1.43 to -0.78)	120.82 (95.67 to 156.54)	85.57 (65.09 to 125.29)	-0.99 (-1.26 to -0.55)

Guatemala	8.93 (5.89 to 12.73)	11.57 (8.34 to 16.05)	0.65 (0.29 to 1.14)	1.21 (0.9 to 1.61)	1.21 (0.82 to 1.54)	-0.5 (-1.16 to 0.35)	33.54 (24.01 to 48.4)	31.84 (22.07 to 41.42)	-0.56 (-1.22 to 0.38)
Guinea	207.59 (149.52 to 279.59)	229.5 (153.29 to 320.43)	0.22 (-0.27 to 0.69)	8.58 (4.37 to 12.53)	6.19 (4.29 to 8.25)	-0.8 (-1.25 to 0.05)	226 (119.66 to 322.34)	173.03 (122.27 to 234.59)	-0.73 (-1.18 to 0.13)
Guinea-Bissau	203.11 (133.1 to 292.64)	218.19 (142.18 to 313.04)	0.14 (-0.11 to 0.41)	9.36 (4.2 to 15.65)	6.3 (4.27 to 8.9)	-0.96 (-1.37 to -0.02)	250.82 (113.42 to 417.2)	176.31 (120.52 to 250.38)	-0.91 (-1.33 to 0.11)
Guyana	32.85 (24.59 to 44.68)	28.72 (21.44 to 38.45)	-0.26 (-0.48 to -0.02)	10.89 (8.39 to 13.8)	9.64 (7.58 to 12.27)	-0.2 (-0.78 to 0.57)	304.72 (247.15 to 370.73)	281.59 (201.26 to 372.84)	-0.25 (-0.8 to 0.53)
Haiti	29.38 (22.01 to 39.39)	27.35 (20.07 to 36.76)	0.18 (-0.07 to 0.47)	11.61 (6.3 to 18.75)	9.47 (5.41 to 14.82)	-0.51 (-1 to 0.3)	340.85 (176.71 to 559.06)	279.57 (152.41 to 465.84)	-0.51 (-1.02 to 0.31)
Honduras	27.21 (18.67 to 38.87)	32.6 (23.42 to 45.34)	0.49 (0.14 to 1.02)	5.09 (3.38 to 7.07)	5.87 (3.78 to 8.08)	-0.05 (-0.58 to 0.75)	127.25 (87.24 to 171.89)	130.01 (80.3 to 181.17)	-0.23 (-0.74 to 0.56)
Hungary	68.86 (46.48 to 96.79)	73.89 (49.26 to 104.25)	-0.02 (-0.34 to 0.33)	21.12 (14.03 to 24.9)	11.35 (8.04 to 17.66)	-0.58 (-1.12 to 0.36)	436.45 (307.22 to 516.15)	290.17 (203.52 to 416.52)	-0.54 (-1.08 to 0.34)
Iceland	36.52 (27.67 to 47.24)	29.57 (23.33 to 37.44)	-0.42 (-0.64 to -0.12)	2.37 (1.74 to 2.8)	1.3 (1.05 to 1.59)	-1 (-1.21 to -0.68)	49.26 (37.49 to 57.22)	25.31 (21.29 to 30.76)	-1.02 (-1.21 to -0.74)
India	1.85 (1.28 to 2.59)	1.92 (1.33 to 2.67)	0.07 (0.02 to 0.11)	0.49 (0.26 to 0.82)	0.38 (0.27 to 0.52)	-0.46 (-0.91 to 0.33)	11.58 (6.19 to 18.92)	9.15 (6.54 to 12.52)	-0.42 (-0.87 to 0.39)
Indonesia	37.92 (28.93 to 49.29)	42.16 (32.22 to 54.9)	0.24 (0.14 to 0.34)	3.45 (2.67 to 4.77)	4.15 (2.83 to 5.53)	0.12 (-0.61 to 1.04)	81.02 (61.67 to 128.38)	83.58 (58.65 to 118.43)	-0.08 (-0.78 to 0.8)
Iran (Islamic Republic of)	22.37 (16.92 to 28.94)	22.17 (16.75 to 28.83)	0 (-0.08 to 0.07)	2.35 (1.7 to 3.17)	1.66 (1.14 to 2.09)	-0.72 (-1.19 to -0.17)	63.95 (49.17 to 85.71)	41.52 (30 to 51.4)	-0.71 (-1.16 to -0.19)
Iraq	48 (37.33 to 62.24)	47.28 (36.48 to 60.47)	-0.04 (-0.23 to 0.16)	5.22 (3.73 to 7.89)	4.51 (3.29 to 6.75)	-0.8 (-1.23 to -0.08)	160.71 (118.35 to 225.59)	118.68 (89.57 to 165.59)	-0.88 (-1.29 to -0.19)
Ireland	87.41 (67.15 to 112.9)	63.93 (48.87 to 81.82)	-0.52 (-0.67 to -0.36)	13.05 (6.92 to 15.29)	4.02 (3.13 to 5.51)	-1.13 (-1.47 to -0.61)	213.45 (137.18 to 245.97)	73.98 (58.78 to 102.76)	-1.12 (-1.42 to -0.68)

Israel	32.9 (25.5 to 41.81)	41.79 (32.32 to 53.31)	-0.47 (-0.62 to -0.28)	3.1 (2.68 to 4.01)	1.47 (1.13 to 2.63)	-1.28 (-1.5 to -0.95)	79.97 (64.25 to 89.79)	33.41 (28.02 to 48.75)	-1.32 (-1.52 to -0.99)
Italy	142.18 (103.42 to 186.58)	116.08 (81.92 to 158.47)	0.36 (0.1 to 0.72)	26.47 (6.95 to 32.07)	3.96 (3.21 to 5.21)	-1.75 (-1.85 to -1.15)	346.56 (140.87 to 408.72)	71.49 (59.52 to 101.47)	-1.63 (-1.77 to -1.11)
Jamaica	32.17 (25.1 to 40.98)	37.21 (28.62 to 47.01)	0.36 (0.1 to 0.63)	3.82 (3.03 to 4.61)	3.76 (2.92 to 4.89)	1.14 (0.04 to 2.84)	111.17 (87.03 to 132.84)	109.05 (83.07 to 141.79)	1.13 (0.06 to 2.81)
Japan	80.54 (63.71 to 100.6)	87.89 (66.27 to 115.21)	-0.15 (-0.37 to 0.11)	5.01 (2.62 to 5.63)	2.12 (1.51 to 2.41)	-1.11 (-1.31 to -0.73)	112.39 (81.44 to 122.71)	54.49 (46.01 to 63.16)	-1.01 (-1.2 to -0.74)
Jordan	11.82 (9 to 14.88)	11.13 (8.5 to 14.1)	0.13 (-0.07 to 0.33)	1.2 (0.77 to 1.58)	0.59 (0.45 to 0.85)	-1.15 (-1.45 to -0.5)	31.32 (19.41 to 41.75)	15.04 (11.83 to 20.43)	-1.08 (-1.42 to -0.4)
Kazakhstan	71.04 (52.67 to 95.26)	67.27 (49.16 to 90.09)	-0.05 (-0.26 to 0.2)	24.24 (7.82 to 31.78)	4.08 (2.13 to 10.5)	10.91 (-0.14 to 26.95)	112.99 (64.62 to 319.01)	790.08 (280.84 to 1057.8)	9.83 (-0.14 to 23.66)
Kenya	224.58 (148.7 to 318.84)	217.15 (144.4 to 307.19)	-0.06 (-0.12 to -0.01)	3.18 (2.19 to 4.23)	3.29 (2.13 to 4.55)	-0.1 (-0.47 to 0.42)	115.61 (83.09 to 145.84)	110.58 (73.65 to 151.01)	-0.14 (-0.49 to 0.35)
Kiribati	13.24 (9.35 to 18.36)	13.86 (9.8 to 19)	0.1 (-0.13 to 0.35)	2.93 (1.39 to 3.96)	2.6 (1.1 to 3.76)	-0.32 (-0.81 to 0.36)	94.53 (41.98 to 136.06)	84.98 (32.75 to 131.35)	-0.31 (-0.87 to 0.47)
Kuwait	21.72 (16.22 to 28.2)	22.2 (16.74 to 29.05)	0.03 (-0.14 to 0.23)	1.1 (0.87 to 1.41)	1.59 (1.32 to 1.88)	-0.81 (-1.19 to -0.35)	48.83 (41.33 to 57.53)	27.43 (21.68 to 34.59)	-0.9 (-1.24 to -0.48)
Kyrgyzstan	20.48 (14.03 to 29.25)	22.97 (15.97 to 32.53)	0.24 (-0.02 to 0.55)	4.55 (3.25 to 5.5)	6.98 (3.67 to 8.7)	0.69 (-0.35 to 1.5)	170.03 (119.03 to 202.94)	257.68 (127.34 to 321.44)	0.69 (-0.46 to 1.57)
Lao People's Democratic Republic	23.84 (17.67 to 31.71)	26.3 (19.44 to 35.02)	0.21 (-0.01 to 0.48)	3.51 (2.14 to 5.85)	3.87 (2.85 to 5.12)	-0.28 (-0.93 to 1.05)	79.81 (46.33 to 166.56)	85.18 (60.73 to 125.23)	-0.28 (-1 to 1.21)
Latvia	124.65 (89.32 to 170.4)	115.43 (81.74 to 159.55)	1.26 (0.72 to 1.84)	16.14 (12.46 to 19.71)	23.99 (16.52 to 30.74)	4.05 (1.23 to 6.7)	618.96 (448.18 to 756.88)	858.23 (599.83 to 1107.14)	3.76 (1.16 to 6.25)
Lebanon	31.4 (23.62 to 41.56)	33.36 (25.27 to 42.81)	0.1 (-0.09 to 0.34)	3.13 (2.23 to 4.65)	1.98 (1.37 to 2.95)	-0.89 (-1.32 to -0.26)	85.02 (59.79 to 135.97)	52.58 (36.5 to 83.97)	-0.83 (-1.27 to -0.17)

Lesotho	194.1 (123.16 to 277.51)	194.58 (125.42 to 281.98)	0.01 (-0.18 to 0.23)	15.02 (11.08 to 21.36)	15.03 (10.74 to 22.75)	-0.08 (-0.75 to 1.03)	327.13 (246.98 to 450.57)	328.74 (235.58 to 491.61)	-0.05 (-0.73 to 1.27)
Liberia	257.67 (169.78 to 365.77)	232.64 (150.75 to 332.45)	0.21 (-0.02 to 0.46)	8.22 (4.37 to 12.12)	5.26 (3.53 to 7.75)	-1.02 (-1.4 to -0.19)	217.43 (121.96 to 312.44)	142.48 (99.67 to 206.63)	-0.98 (-1.37 to -0.13)
Libya	23.43 (17.91 to 31.17)	26.81 (20.35 to 35.94)	0.27 (0.04 to 0.54)	3.08 (2.07 to 4.5)	1.82 (1.29 to 2.63)	-0.78 (-1.27 to 0.09)	157.39 (95.28 to 259.33)	73.68 (50.16 to 106.18)	-0.84 (-1.33 to 0.05)
Lithuania	40.3 (27.95 to 56.94)	44.91 (30.86 to 63.63)	0.23 (-0.03 to 0.49)	7.6 (6.24 to 9.28)	9.44 (6.26 to 12.36)	0.56 (-0.26 to 1.41)	278.52 (229.82 to 338.36)	332.18 (226.25 to 432.38)	0.45 (-0.31 to 1.26)
Luxembourg	103.82 (77.88 to 137.34)	99.54 (77.66 to 124.26)	-0.09 (-0.41 to 0.33)	7.57 (5.39 to 8.72)	3.58 (2.77 to 4.63)	-1.12 (-1.39 to -0.67)	146.98 (111.63 to 165.98)	65.53 (51.42 to 87.21)	-1.14 (-1.4 to -0.71)
Madagascar	273.5 (179.5 to 383.46)	257.04 (167.5 to 362.36)	0.14 (-0.08 to 0.38)	6.78 (4.52 to 8.6)	5.69 (3.7 to 8)	-0.43 (-0.98 to 0.39)	270.13 (179.83 to 344.5)	215.19 (143.67 to 319.7)	-0.46 (-0.99 to 0.36)
Malawi	272.53 (176.43 to 391.65)	287.11 (189.67 to 405.14)	0.13 (-0.11 to 0.4)	4.16 (2.76 to 5.36)	3.77 (2.51 to 5.02)	-0.32 (-0.93 to 0.57)	166.44 (110.88 to 223.68)	140.15 (96.1 to 186.45)	-0.33 (-0.92 to 0.54)
Malaysia	71.68 (54.17 to 94.63)	82.09 (62.13 to 108.85)	0.3 (0 to 0.62)	2.97 (2.2 to 4.08)	3.32 (2.67 to 4.5)	-0.7 (-1.05 to -0.21)	98.61 (79.99 to 126.92)	85.31 (62.68 to 109.54)	-0.62 (-0.97 to -0.12)
Maldives	24.67 (18.47 to 32.6)	25.96 (19.38 to 34.63)	0.1 (-0.13 to 0.34)	1.55 (0.87 to 2.86)	1.37 (0.98 to 1.76)	-0.78 (-1.4 to 0.63)	48.18 (25.29 to 84.75)	34.13 (24.56 to 43.98)	-0.93 (-1.49 to 0.38)
Mali	194.16 (121.78 to 275.94)	206.19 (131.45 to 295.24)	0.11 (-0.13 to 0.36)	8.73 (4.53 to 14.03)	6.32 (4.55 to 8.56)	-0.87 (-1.3 to 0.02)	221.87 (116.5 to 334.23)	162.91 (119.4 to 222.13)	-0.84 (-1.26 to 0.03)
Malta	81.76 (61.95 to 105.44)	71.97 (54.76 to 91.3)	-0.18 (-0.43 to 0.17)	10.74 (6.88 to 12.47)	3.09 (2.4 to 4.78)	-1.27 (-1.51 to -0.78)	179.47 (129.88 to 201.4)	62.51 (50.16 to 87.67)	-1.18 (-1.41 to -0.76)
Marshall Islands	20.96 (15.41 to 28.59)	21.93 (15.94 to 29.98)	0.06 (-0.17 to 0.3)	4.67 (2.9 to 7.14)	4.28 (2.48 to 6.36)	-0.47 (-0.93 to 0.14)	125.29 (76.29 to 193.32)	120.69 (69.5 to 185.68)	-0.39 (-0.88 to 0.27)
Mauritania	242.8 (159.04 to 343.04)	259.24 (169.28 to 364.97)	0.12 (-0.13 to 0.38)	8.37 (4.42 to 11.34)	4.81 (3.2 to 6.57)	-1.16 (-1.51 to -0.37)	219.54 (122.18 to 293.2)	129.18 (86.09 to 179.04)	-1.12 (-1.48 to -0.36)

Mauritius	38.94 (29.54 to 50.43)	36.49 (27.88 to 47.08)	-0.13 (-0.32 to 0.07)	1.92 (1.49 to 3.41)	3.28 (2.11 to 4.18)	0.54 (-0.88 to 1.96)	60.32 (46.82 to 105.05)	99.11 (62.93 to 128.41)	0.58 (-0.83 to 2.09)
Mexico	14.71 (10.93 to 19.28)	15.46 (11.34 to 20.4)	-0.12 (-0.19 to -0.04)	1.17 (1 to 1.8)	1.23 (1.02 to 1.5)	-0.19 (-0.77 to 0.39)	36.44 (32.67 to 49.04)	40.93 (30.98 to 49.16)	-0.05 (-0.74 to 0.59)
Micronesia (Federated States of)	20.82 (15.03 to 28.29)	20.66 (15.17 to 27.81)	-0.01 (-0.21 to 0.22)	4.53 (2.67 to 6.89)	4.23 (2.21 to 6.34)	-0.47 (-1.02 to 0.26)	125.1 (71.62 to 197.85)	116.51 (54.77 to 187.07)	-0.46 (-1.05 to 0.25)
Monaco	165.06 (125.81 to 213.16)	116.39 (88.31 to 151.19)	-0.59 (-0.73 to -0.44)	12.13 (7.87 to 15.65)	8.95 (6.51 to 11.2)	-0.74 (-1.13 to -0.17)	241.88 (165.47 to 307.76)	163.94 (120.87 to 209.79)	-0.8 (-1.15 to -0.26)
Mongolia	29.7 (21.53 to 40.05)	31.06 (22.2 to 41.81)	0.1 (-0.18 to 0.41)	9.34 (6.8 to 12.91)	8.5 (6.07 to 11.92)	0.31 (-0.53 to 1.85)	257.48 (174.65 to 398.01)	234.69 (165.68 to 369.17)	0.33 (-0.54 to 1.98)
Montenegro	206.32 (149.78 to 275.59)	174.29 (126.37 to 235.45)	-0.31 (-0.5 to -0.1)	26.02 (12.27 to 48.5)	29.18 (15.11 to 44.02)	0.27 (-0.54 to 1.59)	494.57 (269.98 to 847.7)	503.33 (284.62 to 761.22)	0.14 (-0.54 to 1.13)
Morocco	19.36 (14.47 to 25.58)	20.3 (15.17 to 26.82)	0.09 (-0.1 to 0.31)	2.71 (1.8 to 4.34)	2.65 (1.64 to 4.2)	-0.63 (-1.11 to 0.16)	78.44 (45.96 to 143.69)	58.75 (39.64 to 94.85)	-0.75 (-1.23 to 0.02)
Mozambique	258.28 (169.01 to 366.26)	303.6 (204.52 to 423.85)	0.38 (0.11 to 0.73)	3.54 (2.68 to 4.68)	3.93 (2.87 to 5.32)	0.03 (-0.6 to 1.16)	137.63 (103.77 to 179.87)	147.26 (110.79 to 191.22)	0.07 (-0.55 to 1.08)
Myanmar	29.67 (21.97 to 39.24)	30.55 (22.28 to 40.46)	0.06 (-0.17 to 0.31)	3.73 (2.31 to 6.17)	3.54 (2.65 to 4.68)	-0.49 (-1.02 to 0.52)	96.62 (55.76 to 199.53)	84.32 (61.48 to 128.87)	-0.56 (-1.13 to 0.59)
Namibia	222.7 (141.51 to 314.97)	229.33 (145.8 to 322.71)	-0.07 (-0.26 to 0.14)	15.07 (11.32 to 19.77)	12.22 (9.21 to 17.35)	-0.44 (-0.97 to 0.37)	330.33 (243.81 to 426.04)	264.06 (197.16 to 377.29)	-0.41 (-0.96 to 0.51)
Nauru	23.39 (17.21 to 31.89)	23.47 (17.03 to 32.06)	-0.01 (-0.22 to 0.21)	4.38 (2.6 to 6.43)	4.23 (2.48 to 6.37)	-0.35 (-0.8 to 0.37)	130.68 (76.03 to 201.8)	128.48 (76.18 to 196.12)	-0.32 (-0.78 to 0.4)
Nepal	1.33 (0.91 to 1.85)	1.32 (0.91 to 1.85)	-0.01 (-0.24 to 0.22)	0.44 (0.22 to 0.8)	0.45 (0.27 to 0.73)	0.06 (-0.7 to 1.49)	10.47 (5.17 to 18.34)	9.9 (5.77 to 16.04)	-0.07 (-0.79 to 1.19)
Netherlands	79.31 (62.73 to 98.23)	58.17 (45.04 to 74.34)	-0.53 (-0.75 to -0.27)	9.23 (5.77 to 11.02)	3.38 (2.58 to 5.09)	-1.36 (-1.57 to -0.75)	161.86 (118.51 to 185.11)	60.39 (46.16 to 92.49)	-1.3 (-1.52 to -0.8)

New Zealand	80.79 (60.55 to 107.75)	88.35 (66.15 to 117.32)	-0.44 (-0.67 to -0.17)	4.38 (3.45 to 5.56)	5.42 (3.72 to 6.17)	-0.9 (-1.14 to -0.55)	190.04 (123.38 to 233.83)	99.57 (75.58 to 112.16)	-0.94 (-1.15 to -0.64)
Nicaragua	15.54 (11.04 to 21.42)	19.22 (14.22 to 25.6)	0.6 (0.3 to 1.04)	2.02 (1.58 to 2.47)	2.33 (1.69 to 2.91)	-0.18 (-0.65 to 0.4)	57.15 (43.49 to 72.26)	53.17 (38.89 to 66.48)	-0.53 (-0.92 to -0.02)
Niger	188.81 (122.33 to 272.86)	208.86 (136.51 to 298.6)	0.12 (-0.13 to 0.41)	8.57 (4.27 to 14.92)	6.05 (4.14 to 8.64)	-0.92 (-1.31 to -0.06)	224.82 (115.26 to 364.68)	159.74 (109.49 to 229.42)	-0.9 (-1.29 to -0.03)
Nigeria	232.17 (161.36 to 312.52)	209.16 (145.95 to 281.5)	0.05 (-0.02 to 0.13)	8.76 (4.87 to 12.92)	4.84 (3.39 to 6.33)	-1.18 (-1.48 to -0.55)	224.85 (127.73 to 328.55)	130.42 (95.24 to 170.05)	-1.13 (-1.43 to -0.5)
Niue	30.31 (22.13 to 40.49)	29.17 (21.55 to 39.52)	0.1 (-0.1 to 0.35)	3.46 (2.31 to 4.6)	3.01 (1.82 to 4.01)	-0.54 (-0.96 to 0.06)	102.83 (68.87 to 138.1)	91.52 (56.57 to 126.69)	-0.5 (-0.98 to 0.22)
North Macedonia	84.86 (61.59 to 114.67)	106.79 (77.46 to 142.95)	-0.48 (-0.66 to -0.29)	26.66 (14.57 to 38.58)	21.48 (11.99 to 29.09)	-0.84 (-1.22 to -0.12)	405.91 (278.81 to 512.95)	351.59 (200.57 to 477.04)	-0.76 (-1.13 to -0.26)
Northern Mariana Islands	73.47 (53.76 to 98.09)	73.3 (54 to 97.44)	-0.05 (-0.3 to 0.22)	5.2 (3.85 to 8.09)	4.2 (3.16 to 6.41)	-0.84 (-1.17 to -0.38)	149.32 (109.09 to 226.03)	122.41 (91.24 to 182.16)	-0.83 (-1.16 to -0.34)
Norway	65.09 (49.46 to 84.87)	64.94 (50.75 to 81.73)	0.02 (-0.16 to 0.24)	2.19 (1.67 to 4.45)	1.94 (1.6 to 2.93)	-0.57 (-1.06 to -0.17)	66.7 (56.48 to 97.53)	46.7 (40.36 to 55.71)	-0.78 (-1.13 to -0.51)
Oman	15.87 (12.36 to 20.46)	16.68 (12.42 to 22.55)	0.06 (-0.24 to 0.41)	1.89 (1.13 to 3.31)	2.28 (1.17 to 3.15)	-0.36 (-1.34 to 1.57)	48.8 (29.3 to 83.42)	56.49 (28.62 to 80.41)	-0.38 (-1.4 to 1.69)
Pakistan	1.77 (1.21 to 2.49)	1.76 (1.21 to 2.49)	0.03 (-0.11 to 0.19)	0.51 (0.3 to 0.78)	0.47 (0.27 to 0.76)	-0.15 (-0.66 to 0.63)	11.89 (6 to 19.54)	11.61 (7.34 to 16.61)	-0.06 (-0.6 to 0.76)
Palau	12.19 (8.61 to 16.78)	12.2 (8.76 to 16.65)	0.01 (-0.18 to 0.24)	1.05 (0.75 to 1.44)	0.86 (0.62 to 1.13)	-0.43 (-0.93 to 0.37)	31.06 (21.32 to 44.07)	25.7 (18.15 to 34.2)	-0.37 (-0.91 to 0.52)
Palestine	39.51 (30.44 to 50.62)	41.5 (31.94 to 53.05)	0.11 (-0.09 to 0.31)	5.66 (2.95 to 8.52)	3.07 (2.17 to 3.81)	-1.21 (-1.52 to -0.52)	139.56 (77.77 to 204.84)	73.67 (56.3 to 91.99)	-1.17 (-1.49 to -0.46)
Panama	50.6 (36.88 to 69.23)	53.28 (38.98 to 71.17)	0.27 (-0.03 to 0.66)	3.08 (2.7 to 3.88)	4.4 (2.74 to 5.91)	0.19 (-0.76 to 1.21)	87.22 (77.94 to 109.76)	118.06 (73.16 to 160.61)	0.18 (-0.74 to 1.22)

Papua New Guinea	18.92 (13.78 to 25.6)	19.85 (14.38 to 27.02)	0.09 (-0.14 to 0.35)	3.45 (2.02 to 5.41)	2.92 (1.65 to 4.87)	-0.19 (-0.7 to 0.66)	90.99 (46.29 to 160.6)	100.5 (55.16 to 165.71)	-0.18 (-0.72 to 0.7)
Paraguay	38.12 (27.79 to 52.54)	35.35 (25.17 to 48.18)	0.36 (0.04 to 0.74)	4.41 (2.59 to 5.71)	3.1 (2.28 to 4.33)	-0.67 (-1.16 to 0.29)	97.94 (64.59 to 121.75)	69.21 (50.93 to 99.49)	-0.65 (-1.13 to 0.25)
Peru	18.64 (13.29 to 25.73)	22.37 (16.6 to 29.45)	0.45 (0.15 to 0.85)	1.82 (1.28 to 2.29)	1.06 (0.74 to 1.61)	-1.02 (-1.4 to -0.25)	58.45 (37.9 to 75.05)	31.36 (22.26 to 44.82)	-1.01 (-1.4 to -0.19)
Philippines	36.12 (27.31 to 46.57)	36.36 (27.49 to 46.48)	0.03 (-0.02 to 0.09)	4.93 (1.89 to 6.76)	4.55 (1.54 to 6.18)	0.8 (-0.01 to 1.89)	63.79 (34.72 to 79.01)	93.85 (53.33 to 120.06)	1.73 (0.18 to 3.08)
Poland	163.22 (117.84 to 219.97)	180.67 (134.69 to 237.58)	0.29 (0.03 to 0.62)	15.7 (12.44 to 17.15)	14.5 (8.97 to 17.83)	-0.04 (-0.62 to 0.43)	310.41 (265.67 to 343.92)	268.43 (185.81 to 330.87)	0.08 (-0.52 to 0.65)
Portugal	38.68 (29.87 to 50.35)	50.01 (37.66 to 65.61)	-0.63 (-0.84 to -0.39)	4.17 (3.33 to 4.65)	2.54 (1.94 to 2.98)	-0.61 (-0.94 to -0.29)	86.38 (71.9 to 95.84)	52.6 (37.28 to 61.1)	-0.6 (-1 to -0.3)
Puerto Rico	50.87 (38.39 to 66.97)	45.31 (34.21 to 60.03)	-0.24 (-0.45 to -0.01)	5.48 (3.36 to 6.75)	2.22 (1.64 to 3.15)	-1.25 (-1.55 to -0.61)	139.68 (97.96 to 167.44)	58.84 (43.1 to 88.55)	-1.2 (-1.51 to -0.6)
Qatar	67.94 (53.36 to 85.71)	65.88 (49.08 to 86.15)	-0.11 (-0.46 to 0.28)	4.54 (2.7 to 6.71)	3.62 (2.28 to 5.12)	-0.73 (-1.3 to 0.39)	118.41 (82.36 to 161.8)	68.76 (48.16 to 93.55)	-0.99 (-1.42 to -0.23)
Republic of Korea	35.43 (26.7 to 46.33)	34.13 (26.26 to 44.01)	-0.06 (-0.26 to 0.2)	1.99 (1.41 to 2.79)	1.31 (0.89 to 1.56)	-1.02 (-1.42 to -0.48)	56.89 (42.02 to 84.97)	32.95 (24.5 to 38.78)	-1.06 (-1.45 to -0.59)
Republic of Moldova	29.12 (21.11 to 39.79)	53.92 (38.64 to 74.57)	1.7 (1.26 to 2.16)	3.15 (2.64 to 5.09)	6.18 (4.36 to 7.97)	11.82 (1.89 to 22.09)	117.55 (99.93 to 180.53)	213.46 (152.94 to 277.06)	10.7 (1.84 to 19.52)
Romania	89.67 (61.41 to 124.65)	103.42 (74.22 to 140.18)	-0.28 (-0.56 to 0.02)	29.07 (17.7 to 35.64)	19.63 (13.09 to 24.8)	-0.71 (-1.07 to -0.26)	500.62 (352.63 to 595.63)	391.07 (248.11 to 513.36)	-0.59 (-0.96 to -0.13)
Russian Federation	80.36 (59.38 to 107.44)	74.16 (54.24 to 100.93)	-0.16 (-0.25 to -0.08)	19.62 (11.24 to 28.15)	18.83 (14.05 to 25.81)	2.58 (-0.51 to 5.11)	448.6 (325.38 to 796.71)	905.93 (577.63 to 1132.95)	3.08 (-0.43 to 6.67)
Rwanda	236.68 (155.96 to 332.09)	231.38 (152.42 to 329.8)	0.06 (-0.16 to 0.31)	5.75 (3.63 to 8.23)	4 (2.69 to 5.42)	-1 (-1.44 to -0.2)	204.85 (128.66 to 287.29)	139.28 (96.31 to 188.36)	-0.99 (-1.44 to -0.17)

Saint Kitts and Nevis	71.34 (54.04 to 94.3)	69.73 (52.76 to 92.73)	0.11 (-0.12 to 0.37)	14.22 (12.16 to 16.52)	12.13 (9.35 to 14.84)	-0.03 (-0.67 to 0.81)	377.41 (323.29 to 442.17)	292.67 (218.58 to 375.52)	-0.2 (-0.82 to 0.64)
Saint Lucia	68.1 (50.69 to 89.53)	71.94 (53.28 to 93.58)	0.14 (-0.13 to 0.41)	11.15 (9.18 to 14.07)	10.21 (7.92 to 12.51)	0.01 (-0.65 to 0.8)	281.09 (233.65 to 356.28)	259.04 (200.65 to 318.56)	0.01 (-0.66 to 0.81)
Saint Vincent and the Grenadines	21.25 (15.61 to 28.49)	24.06 (17.61 to 32.13)	0.33 (0.03 to 0.63)	3.28 (2.79 to 4.46)	4.28 (3.28 to 5.15)	1.42 (0.2 to 2.64)	89.8 (77.35 to 117.79)	110.71 (84.59 to 135.02)	1.24 (0.11 to 2.34)
Samoa	28.49 (20.93 to 38.03)	28.2 (20.81 to 37.71)	0.03 (-0.2 to 0.27)	3.68 (2.46 to 4.91)	3.33 (2.01 to 4.55)	-0.46 (-0.9 to 0.18)	102.49 (68.79 to 142.36)	92.31 (55.95 to 129.64)	-0.43 (-0.93 to 0.31)
San Marino	133.79 (101.1 to 174.61)	97.52 (72.92 to 126.37)	-0.54 (-0.7 to -0.36)	7.15 (4.58 to 9.31)	5.65 (3.61 to 8.03)	-0.49 (-1.08 to 0.52)	133.96 (94.24 to 167.68)	100.44 (65.71 to 147.93)	-0.53 (-1.09 to 0.43)
Sao Tome and Principe	236.35 (153.77 to 333.8)	267.98 (173.63 to 377.64)	0.26 (-0.01 to 0.55)	6.45 (3.52 to 9.18)	5.26 (3.7 to 6.85)	-0.62 (-1.14 to 0.34)	175.19 (103.53 to 244.65)	147.63 (107.47 to 192.48)	-0.56 (-1.08 to 0.41)
Saudi Arabia	77.72 (57.91 to 102.6)	64.77 (48.13 to 85.94)	0.34 (0.07 to 0.66)	7.76 (5.17 to 11.83)	5.11 (3.23 to 6.47)	-0.97 (-1.47 to -0.06)	188.16 (133.86 to 276.67)	120.96 (79.98 to 154.32)	-0.94 (-1.42 to -0.11)
Senegal	270.19 (177.98 to 370.94)	271.6 (179.87 to 378.07)	-0.03 (-0.25 to 0.22)	7.25 (3.83 to 10.53)	5.24 (3.57 to 6.97)	-0.91 (-1.29 to -0.15)	195.66 (111.58 to 272.21)	146.81 (104.26 to 191.16)	-0.85 (-1.25 to -0.11)
Serbia	54.94 (37.85 to 74.82)	81.56 (57.44 to 110.54)	-0.68 (-0.99 to -0.29)	20.41 (12.7 to 26.7)	20.14 (10.69 to 28.06)	0.46 (-0.41 to 1.88)	333.74 (236.3 to 414.94)	298.27 (168.6 to 414.94)	0.25 (-0.55 to 1.51)
Seychelles	74.59 (55.19 to 100.27)	69.88 (52.51 to 93.17)	-0.1 (-0.35 to 0.16)	9.77 (7.64 to 12.44)	8.22 (6.49 to 10.48)	-0.49 (-0.89 to 0.04)	259.63 (203.27 to 333.48)	217.76 (171.8 to 277.37)	-0.49 (-0.87 to 0.04)
Sierra Leone	245.61 (159.8 to 350.36)	256.59 (167.6 to 364.32)	0.1 (-0.15 to 0.38)	7.75 (4.1 to 11.48)	5.55 (3.74 to 8.03)	-0.88 (-1.3 to -0.04)	210.27 (118.28 to 304.1)	161.65 (112.07 to 231.4)	-0.79 (-1.23 to 0.08)
Singapore	38.73 (29.69 to 50.23)	29.98 (23.07 to 39.02)	-0.44 (-0.59 to -0.28)	3.59 (3 to 5)	1.47 (1.09 to 2.13)	-1.38 (-1.61 to -1.16)	118.82 (97.34 to 162.79)	42.36 (32.25 to 54.5)	-1.44 (-1.68 to -1.21)
Slovakia	66.09 (48.67 to 87.2)	62.38 (44.82 to 83.75)	0.84 (0.19 to 1.7)	4.85 (3.5 to 9.02)	5.29 (3.36 to 8.49)	1.12 (-0.3 to 3.08)	138.66 (114.65 to 200.83)	142.16 (91.81 to 195.86)	0.84 (-0.43 to 2.48)

Slovenia	246.63 (171.75 to 338.51)	171.13 (127.24 to 221.54)	-0.65 (-0.89 to -0.33)	68.92 (14.86 to 100.61)	14.56 (8.43 to 18.75)	-1.73 (-1.81 to -0.99)	1010.07 (306.39 to 1468.61)	200.18 (144.7 to 255)	-1.73 (-1.82 to -1.07)
Solomon Islands	16.58 (11.81 to 22.85)	16.24 (11.59 to 22.12)	-0.03 (-0.27 to 0.2)	4.07 (2.24 to 6.66)	3.94 (2.19 to 6.23)	-0.37 (-0.85 to 0.34)	122.37 (61.88 to 215.12)	116.27 (60.52 to 199.8)	-0.4 (-0.88 to 0.3)
Somalia	234.01 (154.85 to 330.47)	225.14 (146.83 to 327.12)	0.13 (-0.11 to 0.4)	5.21 (3.23 to 7.61)	4.2 (2.73 to 6.33)	-0.41 (-1.06 to 0.73)	193.47 (117.68 to 284.12)	155.66 (104.35 to 222.94)	-0.41 (-0.99 to 0.59)
South Africa	244.4 (162.65 to 337.71)	274.11 (183.27 to 384.31)	-0.21 (-0.28 to -0.13)	11.73 (9 to 14.29)	10.55 (8.76 to 13.32)	-0.37 (-0.66 to -0.05)	284.8 (230.1 to 332.04)	230.99 (192.82 to 288.3)	-0.51 (-0.78 to -0.15)
South Sudan	248.51 (164.31 to 355.64)	258.47 (173.64 to 364.33)	0.06 (-0.15 to 0.31)	4.6 (2.43 to 6.66)	3.16 (1.91 to 4.76)	-0.74 (-1.19 to 0.1)	182.67 (100.9 to 261.94)	127.01 (80.12 to 187.27)	-0.7 (-1.16 to 0.09)
Spain	130.26 (97.04 to 169.48)	87.53 (65.48 to 113.39)	-0.63 (-0.83 to -0.45)	10.68 (6.58 to 12.23)	5.31 (4.32 to 6.17)	-1.14 (-1.3 to -0.76)	180.05 (143.38 to 199.14)	101.12 (81.8 to 117.2)	-0.98 (-1.13 to -0.78)
Sri Lanka	123.02 (91.06 to 163.93)	131.6 (96.72 to 173.83)	0.11 (-0.15 to 0.4)	15.06 (9.52 to 18.41)	7.81 (5.21 to 12.18)	-0.16 (-0.96 to 1.2)	363.38 (242.37 to 435.92)	206.63 (141.39 to 304.15)	-0.15 (-0.92 to 1.11)
Sudan	14.8 (10.84 to 20.08)	16.41 (12.03 to 21.73)	0.2 (-0.03 to 0.45)	3.36 (1.65 to 6.25)	2.4 (1.54 to 3.9)	-0.86 (-1.28 to -0.08)	100.98 (44.83 to 224.83)	68.1 (41.21 to 116.88)	-0.91 (-1.37 to 0.05)
Suriname	41.73 (31.73 to 54.8)	38.7 (29.39 to 51.82)	0.16 (-0.13 to 0.44)	5.24 (4.21 to 7.36)	5.21 (3.92 to 6.62)	0.13 (-0.54 to 1.05)	156.23 (125.18 to 208.36)	147.32 (109.92 to 187.75)	0.09 (-0.57 to 0.98)
Sweden	127.86 (97.69 to 167.39)	129.86 (100.02 to 166.25)	-0.07 (-0.29 to 0.19)	4.61 (3.66 to 7)	2.91 (2.21 to 5.32)	-0.56 (-0.98 to -0.05)	135.57 (106.09 to 157.2)	74.59 (61.55 to 102.64)	-0.72 (-1.05 to -0.28)
Switzerland	68.61 (51.26 to 88.88)	45.82 (37.26 to 56.03)	-0.67 (-0.9 to -0.37)	4.81 (3.79 to 6.04)	2.56 (1.99 to 3.83)	-1.04 (-1.29 to -0.63)	107.56 (84.95 to 128.89)	49.76 (40.61 to 68.36)	-1.11 (-1.33 to -0.77)
Syrian Arab Republic	25.68 (18.96 to 34.42)	23.1 (17.02 to 30.87)	0.26 (0.01 to 0.53)	3.51 (2.37 to 5.35)	2.54 (1.77 to 3.99)	-0.76 (-1.24 to -0.07)	107.08 (71.19 to 168.68)	66.56 (45.24 to 105.05)	-0.85 (-1.3 to -0.15)
Taiwan (Province of China)	51.31 (38.22 to 66.48)	45.56 (35.15 to 56.94)	-0.18 (-0.41 to 0.08)	2.78 (2.13 to 3.12)	1.37 (1.04 to 1.86)	-1.06 (-1.34 to -0.64)	67.82 (55.76 to 75.8)	40.3 (30.27 to 53.28)	-0.81 (-1.15 to -0.38)

Tajikistan	4.8 (3.55 to 6.34)	4.65 (3.48 to 6.17)	-0.03 (-0.23 to 0.18)	1.12 (0.7 to 1.48)	0.95 (0.64 to 1.21)	0.25 (-0.38 to 1.27)	30.23 (21.84 to 37.43)	32.44 (21.5 to 42.45)	0.06 (-0.53 to 1.02)
Thailand	18.75 (13.72 to 25.01)	20.48 (14.98 to 27.24)	0.19 (-0.01 to 0.43)	0.65 (0.39 to 1.18)	0.62 (0.44 to 0.94)	-0.48 (-1.08 to 0.67)	17.82 (11.54 to 32.21)	19.69 (14.03 to 26.44)	-0.22 (-0.97 to 1.01)
Timor-Leste	28.74 (21.58 to 38.12)	34.3 (25.47 to 45.41)	0.38 (0.11 to 0.69)	2.89 (1.83 to 4.28)	3.89 (2.66 to 5.4)	0.34 (-0.55 to 1.77)	62.69 (38.46 to 113.72)	80.64 (55.7 to 119.31)	0.33 (-0.62 to 1.81)
Togo	236.8 (154.79 to 334.64)	234.74 (153.46 to 331.83)	0 (-0.22 to 0.25)	8.17 (4.45 to 11.55)	5.37 (3.78 to 7.27)	-0.93 (-1.31 to -0.21)	215.27 (124.12 to 293.71)	152.07 (110.81 to 204.95)	-0.85 (-1.24 to -0.14)
Tokelau	28.01 (20.65 to 37.32)	30.34 (22.42 to 40.33)	0.18 (-0.04 to 0.44)	3.48 (2.24 to 4.82)	2.89 (1.74 to 4)	-0.71 (-1.12 to -0.02)	97.4 (62.12 to 138.57)	81.31 (47.85 to 115.4)	-0.68 (-1.13 to 0.12)
Tonga	28.24 (20.65 to 38.06)	28.05 (20.66 to 38.01)	-0.01 (-0.21 to 0.26)	1.74 (1.14 to 2.31)	1.79 (1.31 to 2.3)	-0.35 (-0.82 to 0.4)	52.88 (39.89 to 68.78)	50.33 (34.33 to 67.96)	-0.29 (-0.8 to 0.51)
Trinidad and Tobago	34.98 (26.5 to 45.75)	35.12 (26.43 to 47.04)	0 (-0.25 to 0.29)	5.4 (4.3 to 6.86)	4.34 (3.18 to 5.87)	-0.2 (-0.82 to 0.86)	149.68 (124.01 to 185.36)	124.47 (89.08 to 170.37)	-0.14 (-0.78 to 0.93)
Tunisia	23.09 (17.08 to 30.46)	25.15 (18.99 to 33.11)	0.18 (-0.04 to 0.43)	2.69 (1.97 to 3.96)	1.89 (1.27 to 2.93)	-0.75 (-1.23 to -0.08)	74.97 (54.02 to 113.06)	46.98 (31.69 to 74.28)	-0.75 (-1.22 to -0.09)
Turkey	34.93 (26.75 to 45.44)	28.71 (23.34 to 35.27)	-0.41 (-0.67 to -0.09)	2.69 (1.9 to 4.12)	1.36 (0.97 to 2.27)	-1.17 (-1.47 to -0.55)	90.34 (64.54 to 128.21)	42.54 (33.75 to 57.64)	-1.17 (-1.47 to -0.61)
Turkmenistan	39.45 (29.17 to 53.13)	41.49 (30.51 to 56.22)	0.12 (-0.11 to 0.37)	18.68 (13.37 to 23.49)	14.73 (10.64 to 22.77)	0.03 (-0.74 to 1.42)	651.94 (461.38 to 813)	541.83 (398.43 to 795.19)	0.15 (-0.63 to 1.53)
Tuvalu	22.4 (16.53 to 30.58)	24.43 (17.78 to 33.27)	0.17 (-0.06 to 0.42)	4.36 (2.58 to 6.36)	3.66 (2.19 to 5.15)	-0.67 (-1.1 to 0.06)	128.62 (72.75 to 200.33)	104.88 (63.41 to 150.56)	-0.68 (-1.16 to 0.15)
Uganda	174.61 (106.88 to 256.24)	178.4 (108.85 to 262.58)	-0.03 (-0.25 to 0.2)	3.62 (2.19 to 4.83)	4.11 (2.74 to 5.38)	-0.56 (-1.01 to 0.14)	146.3 (97.78 to 191.67)	126.58 (77.52 to 175.64)	-0.52 (-0.97 to 0.22)
Ukraine	77.87 (58.24 to 102.27)	72.52 (53.85 to 94.85)	-0.15 (-0.34 to 0.04)	17 (13.29 to 21.48)	23.88 (18.17 to 31.75)	0.92 (0.05 to 2.14)	555.12 (436.61 to 707.8)	848.29 (646.33 to 1167.48)	1.2 (0.25 to 2.5)

United Arab Emirates	20.99 (16.01 to 27.7)	21.02 (15.68 to 27.58)	0 (-0.2 to 0.25)	2.84 (1.41 to 5.74)	1.72 (0.81 to 3.54)	-1.01 (-1.36 to -0.39)	79.41 (43.06 to 152.63)	46.27 (24.56 to 90.61)	-1 (-1.35 to -0.35)
United Kingdom	50.43 (39.17 to 63.92)	41.52 (33.5 to 50.94)	-0.37 (-0.53 to -0.2)	3.21 (2.66 to 5.58)	2.94 (2.49 to 4.03)	-0.24 (-0.74 to 0.06)	93.61 (80.95 to 121.53)	68.99 (61.19 to 81.92)	-0.47 (-0.79 to -0.25)
United Republic of Tanzania	259.81 (170.29 to 358.39)	288.34 (187.59 to 400.67)	0.22 (-0.02 to 0.5)	5.18 (3.54 to 6.47)	4.21 (2.63 to 5.91)	-0.44 (-1.02 to 0.52)	193.34 (133.19 to 248.56)	161.29 (103.82 to 239.11)	-0.38 (-0.95 to 0.58)
United States of America	200.63 (158.89 to 253.28)	193.94 (151.45 to 249.3)	-0.05 (-0.28 to 0.22)	8.32 (6.31 to 9.22)	6.05 (5.28 to 6.84)	-0.68 (-0.88 to -0.31)	238.24 (183.41 to 264.35)	161.32 (145.2 to 184.76)	-0.73 (-0.9 to -0.37)
United States Virgin Islands	53.53 (40.56 to 70.22)	50.55 (37.7 to 67.01)	-0.11 (-0.32 to 0.14)	9.79 (7.11 to 12.85)	10.14 (7.61 to 13.31)	0.07 (-0.56 to 0.92)	268.01 (207.27 to 362.39)	249.69 (173.12 to 328.07)	-0.08 (-0.69 to 0.81)
Uruguay	115.09 (82.15 to 156.59)	135.23 (95.49 to 185.1)	-0.4 (-0.59 to -0.19)	11.81 (7.91 to 14.24)	5.66 (4.64 to 7.93)	-1.08 (-1.38 to -0.47)	277.33 (192.78 to 332.25)	129.95 (108.65 to 179.53)	-1.08 (-1.38 to -0.54)
Uzbekistan	2.19 (1.61 to 2.9)	2.06 (1.51 to 2.72)	0.14 (-0.09 to 0.37)	0.65 (0.3 to 1.32)	1.35 (1.07 to 1.76)	1.46 (-0.16 to 5.96)	20.26 (11.21 to 41.39)	44.34 (34.1 to 55.64)	1.52 (-0.16 to 4.72)
Vanuatu	23.43 (17.11 to 31.98)	22.14 (16.23 to 29.87)	-0.1 (-0.32 to 0.15)	3.57 (2.13 to 5.27)	3.75 (2.11 to 5.51)	-0.23 (-0.74 to 0.55)	98.87 (57.78 to 153.61)	110.09 (60.49 to 167.09)	-0.14 (-0.7 to 0.75)
Venezuela (Bolivarian Republic of)	19.2 (13.17 to 27.35)	18.02 (12.36 to 25.62)	-0.03 (-0.35 to 0.39)	5.16 (2.43 to 6.71)	2.93 (1.99 to 3.86)	-1.09 (-1.37 to -0.38)	113.74 (65.08 to 141.48)	62.46 (46.2 to 81.7)	-1.06 (-1.35 to -0.4)
Viet Nam	44.01 (32.85 to 56.87)	51.34 (40 to 66.01)	0.44 (0.11 to 0.84)	4.35 (3.12 to 6.44)	4.09 (3 to 5.48)	0.02 (-0.7 to 1.28)	97.84 (71.18 to 158.22)	87.67 (63.42 to 131.99)	0.1 (-0.65 to 1.35)
Yemen	13.33 (9.83 to 17.79)	14.58 (10.78 to 19.45)	0.15 (-0.05 to 0.37)	2.88 (1.6 to 5.3)	3 (1.59 to 5.55)	-0.77 (-1.25 to 0.12)	89.56 (43 to 199.12)	66 (39.27 to 121.04)	-0.79 (-1.28 to 0.21)
Zambia	243.6 (154.23 to 346.24)	259.13 (169.22 to 369.52)	0.13 (-0.1 to 0.39)	4.73 (2.81 to 6.53)	3.97 (2.35 to 5.34)	0.25 (-0.54 to 1.64)	153.53 (93.68 to 210.2)	161.51 (101.66 to 225.07)	0.14 (-0.62 to 1.44)
Zimbabwe	227.56 (158.97 to 310.98)	227.04 (148.2 to 328.16)	0.01 (-0.39 to 0.45)	14.84 (10.97 to 17.86)	15.16 (10.94 to 19.38)	0.15 (-0.47 to 1)	328.95 (240.4 to 393.68)	348.77 (248.95 to 445.19)	0.24 (-0.41 to 1.12)

DALY, Disability-Adjusted Life Year; CI, confidence interval.

Table S2. Age-standardized prevalence, death, DALYs rates of the alcoholic cardiomyopathy, and their percentage changes with 95% UIs from 1990 to 2019 at national level, both sexs.

Countries	Prevalence			Deaths			DALYs		
	1990 age-standardized rate per 100,000 people	2019 age-standardized rate per 100,000 people	Percentage change in age-standardized rates, 1990–2019	1990 age-standardized rate per 100,000 people	2019 age-standardized rate per 100,000 people	Percentage change in age-standardized rates, 1990–2019	1990 age-standardized rate per 100,000 people	2019 age-standardized rate per 100,000 people	Percentage change in age-standardized rates, 1990–2019
Afghanistan	1.46 (1.03 to 1.95)	1.6 (1.13 to 2.14)	0.1 (-0.01 to 0.22)	0.79 (0.17 to 1.78)	0.39 (0.1 to 0.8)	-0.51 (-0.7 to -0.06)	20.75 (4.79 to 41.59)	9.81 (2.87 to 17.68)	-0.53 (-0.71 to -0.07)
Albania	12.03 (8.69 to 16.38)	10.13 (7.24 to 13.83)	-0.16 (-0.26 to -0.03)	1.4 (0.98 to 1.8)	0.73 (0.44 to 1.29)	-0.48 (-0.67 to -0.08)	32.18 (23.67 to 44.19)	19.61 (11.11 to 36.02)	-0.39 (-0.6 to -0.02)
Algeria	1.41 (1.01 to 1.89)	1.47 (1.07 to 1.99)	0.04 (-0.06 to 0.15)	0.24 (0.13 to 0.39)	0.11 (0.05 to 0.18)	-0.54 (-0.77 to -0.09)	5.64 (3.32 to 8.66)	2.84 (1.44 to 4.54)	-0.5 (-0.73 to -0.05)
American Samoa	3.25 (2.38 to 4.4)	3.25 (2.32 to 4.37)	0 (-0.11 to 0.14)	0.58 (0.37 to 0.8)	0.15 (0.11 to 0.22)	-0.74 (-0.83 to -0.41)	16.16 (10.26 to 22.37)	4.42 (3.13 to 6.65)	-0.73 (-0.82 to -0.38)
Andorra	35.93 (26.44 to 46.79)	25.9 (18.88 to 34.05)	-0.28 (-0.36 to -0.18)	1.94 (1.19 to 3.02)	1.09 (0.76 to 1.49)	-0.44 (-0.66 to -0.06)	53.2 (34.52 to 80.71)	30.38 (21.53 to 42.1)	-0.43 (-0.66 to -0.07)
Angola	1.59 (1.01 to 2.36)	1.72 (1.11 to 2.55)	0.08 (-0.04 to 0.22)	0.13 (0.06 to 0.23)	0.08 (0.05 to 0.14)	-0.35 (-0.63 to 0.3)	4.17 (1.97 to 7.43)	2.79 (1.68 to 4.47)	-0.33 (-0.62 to 0.33)
Antigua and Barbuda	3.09 (2.25 to 4.09)	3.19 (2.26 to 4.36)	0.03 (-0.16 to 0.24)	0.26 (0.19 to 0.38)	0.49 (0.28 to 0.66)	0.88 (-0.05 to 2.07)	7.89 (5.85 to 11.67)	14.36 (8.32 to 19.29)	0.82 (-0.08 to 1.94)
Argentina	1.3 (0.73 to 2.12)	2.65 (1.58 to 4.02)	1.04 (0.54 to 1.76)	1.09 (0.78 to 1.46)	0.16 (0.12 to 0.21)	-0.85 (-0.9 to -0.8)	33.78 (24.63 to 45.03)	5.21 (3.92 to 6.83)	-0.85 (-0.89 to -0.79)

Armenia	27.01 (20.12 to 35.45)	13.61 (10.13 to 18.09)	-0.5 (-0.55 to -0.43)	5.31 (3.6 to 6.84)	1.37 (0.92 to 2.19)	-0.74 (-0.85 to -0.53)	121.06 (96.21 to 150.97)	31.23 (20.91 to 53.06)	-0.74 (-0.84 to -0.55)
Australia	52.61 (38.67 to 69.84)	38.95 (28.5 to 52.31)	-0.26 (-0.34 to -0.18)	1.78 (1.42 to 2.19)	1.04 (0.75 to 1.38)	-0.41 (-0.63 to -0.23)	56.61 (46.89 to 66.88)	33.8 (25.14 to 42.85)	-0.4 (-0.6 to -0.26)
Austria	21.38 (15.61 to 28.35)	15.5 (11.72 to 19.94)	-0.27 (-0.38 to -0.13)	0.3 (0.22 to 0.86)	0.75 (0.51 to 1.05)	1.5 (-0.37 to 2.89)	9.11 (6.98 to 18.9)	19.68 (12.95 to 28.01)	1.16 (-0.31 to 2.15)
Azerbaijan	1.9 (1.36 to 2.54)	1.83 (1.32 to 2.44)	-0.04 (-0.14 to 0.08)	0.52 (0.23 to 1.22)	0.47 (0.22 to 1.24)	-0.09 (-0.42 to 0.42)	18.15 (8.4 to 43.59)	15.29 (6.92 to 40.94)	-0.16 (-0.48 to 0.35)
Bahamas	7.18 (5.19 to 9.82)	7.08 (5.17 to 9.76)	-0.01 (-0.14 to 0.13)	1.71 (1.27 to 2.39)	2.05 (1.5 to 2.73)	0.2 (-0.18 to 0.77)	55.55 (41.85 to 78.14)	67.53 (48.47 to 88.62)	0.22 (-0.16 to 0.77)
Bahrain	4.84 (3.59 to 6.42)	4.94 (3.69 to 6.55)	0.02 (-0.09 to 0.15)	0.45 (0.29 to 0.84)	0.26 (0.16 to 0.39)	-0.42 (-0.73 to 0.09)	9.58 (6.41 to 16.39)	5.33 (3.41 to 7.85)	-0.44 (-0.72 to 0)
Bangladesh	0.67 (0.46 to 0.91)	0.7 (0.49 to 0.96)	0.05 (-0.06 to 0.18)	0.21 (0.09 to 0.36)	0.14 (0.07 to 0.23)	-0.32 (-0.67 to 0.54)	5.76 (2.28 to 9.87)	3.81 (1.89 to 6.28)	-0.34 (-0.69 to 0.55)
Barbados	16.29 (12.1 to 21.45)	17.79 (13.08 to 24.03)	0.09 (-0.04 to 0.25)	1.39 (1.07 to 1.83)	1.76 (1.26 to 2.25)	0.27 (-0.14 to 0.8)	44.87 (35.61 to 58.22)	53.76 (38.58 to 68.77)	0.2 (-0.18 to 0.67)
Belarus	32.99 (23.79 to 45.22)	42.14 (30.62 to 57.72)	0.28 (0.15 to 0.43)	6.12 (4.25 to 8.39)	8.36 (5.69 to 11.45)	0.36 (-0.06 to 0.92)	213.74 (147.11 to 299.81)	323.82 (220.53 to 437.92)	0.52 (0.05 to 1.12)
Belgium	18.82 (13.72 to 24.91)	17.62 (13.95 to 21.91)	-0.06 (-0.25 to 0.17)	0.8 (0.59 to 0.97)	0.56 (0.39 to 0.74)	-0.3 (-0.55 to -0.08)	21.37 (17.11 to 24.97)	16.46 (11.77 to 21.7)	-0.23 (-0.49 to 0)
Belize	3.56 (2.59 to 4.87)	3.89 (2.79 to 5.41)	0.09 (-0.04 to 0.24)	0.29 (0.17 to 0.46)	0.52 (0.34 to 0.66)	0.76 (0.01 to 2.14)	9.4 (5.38 to 15.08)	16.39 (10.87 to 20.7)	0.74 (0 to 2.17)
Benin	3.64 (2.22 to 5.74)	3.87 (2.35 to 6.12)	0.06 (-0.06 to 0.19)	0.39 (0.21 to 0.65)	0.15 (0.08 to 0.25)	-0.63 (-0.78 to -0.32)	12.55 (6.92 to 20.41)	4.85 (2.86 to 8.22)	-0.61 (-0.77 to -0.32)
Bermuda	4.29 (3.13 to 5.72)	4.1 (2.97 to 5.54)	-0.04 (-0.15 to 0.07)	0.33 (0.2 to 0.63)	0.37 (0.22 to 0.5)	0.12 (-0.51 to 1.14)	9.92 (6.18 to 18.07)	11.07 (6.71 to 14.82)	0.12 (-0.5 to 1.05)

Bhutan	0.55 (0.37 to 0.76)	0.61 (0.42 to 0.86)	0.11 (-0.01 to 0.25)	0.2 (0.07 to 0.41)	0.14 (0.08 to 0.22)	-0.3 (-0.63 to 0.53)	5.1 (1.92 to 9.94)	3.5 (2.13 to 5.65)	-0.31 (-0.64 to 0.51)
Bolivia (Plurinational State of)	0.16 (0.06 to 0.35)	0.43 (0.24 to 0.71)	1.61 (0.74 to 3.68)	0.22 (0.1 to 0.44)	0.1 (0.06 to 0.15)	-0.54 (-0.76 to -0.04)	5.25 (2.4 to 9.41)	2.41 (1.48 to 3.52)	-0.54 (-0.75 to -0.07)
Bosnia and Herzegovina	19.04 (13.58 to 25.93)	15.65 (10.83 to 21.54)	-0.18 (-0.28 to -0.07)	2.09 (1.05 to 3.44)	1.57 (0.72 to 2.65)	-0.25 (-0.54 to 0.16)	47.75 (25.07 to 74.29)	40.29 (17.7 to 70.15)	-0.16 (-0.47 to 0.29)
Botswana	0.54 (0.37 to 0.74)	0.54 (0.37 to 0.75)	0 (-0.1 to 0.1)	0.06 (0.04 to 0.08)	0.04 (0.02 to 0.05)	-0.35 (-0.59 to 0.12)	1.67 (1.11 to 2.58)	1.17 (0.71 to 1.8)	-0.3 (-0.58 to 0.16)
Brazil	4.35 (2.81 to 6.32)	4.57 (3.09 to 6.6)	0.05 (-0.01 to 0.14)	1.6 (1.24 to 2.05)	0.5 (0.38 to 0.62)	-0.69 (-0.76 to -0.61)	55.92 (43.19 to 70.37)	17.84 (13.73 to 21.97)	-0.68 (-0.75 to -0.6)
Brunei Darussalam	11.12 (8.36 to 14.82)	9.17 (6.66 to 12.49)	-0.18 (-0.26 to -0.08)	2.45 (1.64 to 3.58)	0.84 (0.61 to 1.18)	-0.66 (-0.78 to -0.47)	74.46 (51.05 to 108.08)	25.23 (18.84 to 35.18)	-0.66 (-0.77 to -0.48)
Bulgaria	0.82 (0.57 to 1.13)	0.71 (0.49 to 1.02)	-0.13 (-0.25 to 0)	0.21 (0.12 to 0.31)	0.1 (0.07 to 0.17)	-0.51 (-0.67 to -0.16)	6.6 (3.94 to 9.44)	3.42 (2.37 to 5.03)	-0.48 (-0.65 to -0.11)
Burkina Faso	4.44 (2.68 to 6.89)	4.34 (2.6 to 6.65)	-0.02 (-0.13 to 0.11)	0.56 (0.31 to 0.98)	0.24 (0.13 to 0.41)	-0.58 (-0.77 to -0.26)	17.58 (9.89 to 30.42)	7.49 (4.35 to 12.99)	-0.57 (-0.76 to -0.26)
Burundi	3.97 (2.52 to 5.78)	3.91 (2.47 to 5.76)	-0.01 (-0.11 to 0.1)	0.15 (0.06 to 0.36)	0.05 (0.02 to 0.11)	-0.65 (-0.87 to -0.1)	5.64 (2.4 to 12.72)	2.13 (1.08 to 3.97)	-0.62 (-0.85 to -0.08)
Cabo Verde	4.16 (2.59 to 6.37)	4.57 (2.9 to 6.76)	0.1 (-0.02 to 0.22)	0.06 (0.04 to 0.1)	0.08 (0.05 to 0.11)	0.19 (-0.27 to 1.03)	2.45 (1.7 to 3.57)	2.77 (1.91 to 3.93)	0.13 (-0.25 to 0.72)
Cambodia	0.98 (0.73 to 1.31)	1.07 (0.8 to 1.42)	0.09 (-0.04 to 0.23)	0.14 (0.07 to 0.31)	0.11 (0.07 to 0.22)	-0.21 (-0.54 to 0.57)	4.32 (1.89 to 9.37)	3.34 (1.96 to 6.73)	-0.23 (-0.56 to 0.59)
Cameroon	3.28 (2.25 to 4.56)	4.16 (2.48 to 6.5)	0.27 (-0.06 to 0.66)	0.44 (0.23 to 0.76)	0.2 (0.12 to 0.34)	-0.54 (-0.76 to -0.13)	14.48 (7.81 to 24.94)	6.9 (4.03 to 11.38)	-0.52 (-0.75 to -0.1)
Canada	81.5 (59.22 to 109.38)	40.89 (31.4 to 52.42)	-0.5 (-0.57 to -0.41)	0.89 (0.71 to 1.06)	0.64 (0.47 to 0.81)	-0.28 (-0.49 to -0.1)	32.28 (27.58 to 38.14)	21.4 (17 to 26.37)	-0.34 (-0.48 to -0.21)

Central African Republic	1.94 (1.24 to 2.89)	1.78 (1.13 to 2.69)	-0.08 (-0.18 to 0.03)	0.2 (0.09 to 0.37)	0.14 (0.07 to 0.28)	-0.29 (-0.58 to 0.2)	6.25 (2.7 to 11.78)	4.42 (2.02 to 8.69)	-0.29 (-0.56 to 0.15)
Chad	3.96 (2.35 to 6.06)	4.47 (2.62 to 6.85)	0.13 (0 to 0.29)	0.41 (0.21 to 0.77)	0.19 (0.1 to 0.34)	-0.54 (-0.75 to -0.17)	13.11 (6.67 to 23.99)	6.24 (3.28 to 11.14)	-0.52 (-0.74 to -0.17)
Chile	6.72 (4.16 to 9.89)	6.19 (4.21 to 8.52)	-0.08 (-0.23 to 0.12)	1.01 (0.76 to 1.24)	0.24 (0.17 to 0.31)	-0.76 (-0.83 to -0.68)	30.22 (22.75 to 36.82)	8.2 (6.11 to 10.48)	-0.73 (-0.81 to -0.64)
China	1.61 (1.18 to 2.16)	1.77 (1.29 to 2.38)	0.1 (0.06 to 0.14)	0.11 (0.05 to 0.23)	0.13 (0.09 to 0.17)	0.23 (-0.52 to 1.76)	3.41 (1.89 to 6.89)	4.56 (3.01 to 6.06)	0.34 (-0.44 to 1.63)
Colombia	1.05 (0.76 to 1.44)	0.96 (0.71 to 1.29)	-0.09 (-0.19 to 0.03)	0.39 (0.27 to 0.45)	0.03 (0.02 to 0.05)	-0.92 (-0.95 to -0.86)	10.96 (7.82 to 12.53)	1.09 (0.74 to 1.66)	-0.9 (-0.93 to -0.85)
Comoros	1.82 (1.19 to 2.67)	1.86 (1.2 to 2.69)	0.02 (-0.09 to 0.15)	0.04 (0.02 to 0.09)	0.03 (0.01 to 0.05)	-0.42 (-0.74 to 0.43)	1.54 (0.6 to 3.02)	0.99 (0.39 to 1.87)	-0.36 (-0.67 to 0.5)
Congo	1.6 (1.01 to 2.36)	1.63 (1.03 to 2.37)	0.02 (-0.09 to 0.15)	0.14 (0.07 to 0.25)	0.07 (0.04 to 0.12)	-0.51 (-0.76 to -0.02)	4.79 (2.11 to 8.48)	2.37 (1.36 to 3.85)	-0.51 (-0.77 to 0.04)
Cook Islands	0.49 (0.35 to 0.65)	0.48 (0.34 to 0.65)	-0.01 (-0.11 to 0.1)	0.1 (0.06 to 0.14)	0.04 (0.03 to 0.06)	-0.58 (-0.72 to -0.36)	3.02 (1.95 to 4.21)	1.42 (0.94 to 1.98)	-0.53 (-0.69 to -0.27)
Costa Rica	2.31 (1.57 to 3.27)	2.38 (1.66 to 3.23)	0.03 (-0.12 to 0.22)	0.36 (0.26 to 0.47)	0.2 (0.13 to 0.29)	-0.43 (-0.64 to -0.14)	11.81 (8.72 to 15.07)	7.06 (4.72 to 9.99)	-0.4 (-0.61 to -0.11)
Croatia	23.81 (16.89 to 32.63)	18.13 (13.49 to 23.97)	-0.24 (-0.42 to 0)	1.56 (1.26 to 2.07)	2.61 (1.87 to 3.49)	0.68 (0.11 to 1.29)	31.64 (26.26 to 46.53)	60.19 (40.83 to 82.72)	0.9 (0.16 to 1.66)
Cuba	17.87 (12.82 to 24.26)	22.91 (16.24 to 31.53)	0.28 (0.11 to 0.47)	0.89 (0.73 to 1.29)	2.67 (1.95 to 3.36)	1.99 (0.94 to 3.08)	30.25 (25.07 to 42.23)	78.85 (58.48 to 98.99)	1.61 (0.74 to 2.53)
Cyprus	5.74 (4.04 to 7.97)	5.22 (3.89 to 7.05)	-0.09 (-0.23 to 0.12)	0.52 (0.31 to 0.79)	0.22 (0.15 to 0.31)	-0.58 (-0.73 to -0.32)	14.8 (9.05 to 21.54)	6.41 (4.67 to 9.09)	-0.57 (-0.71 to -0.33)
Czechia	5.82 (4.02 to 8.32)	6.64 (4.6 to 9.19)	0.14 (-0.05 to 0.41)	0.1 (0.07 to 0.19)	0.54 (0.31 to 0.73)	4.45 (1.05 to 7.68)	3.79 (2.9 to 6.56)	19.32 (11.33 to 26.28)	4.09 (1.23 to 6.83)

Cote d'Ivoire	6.28 (3.75 to 9.73)	6.31 (3.79 to 10.07)	0 (-0.12 to 0.14)	0.56 (0.29 to 0.93)	0.19 (0.11 to 0.3)	-0.66 (-0.81 to -0.37)	17.95 (9.79 to 29.74)	6.41 (3.92 to 10)	-0.64 (-0.79 to -0.34)
Democratic People's Republic of Korea	2.09 (1.54 to 2.8)	2.08 (1.52 to 2.79)	-0.01 (-0.11 to 0.1)	0.22 (0.11 to 0.46)	0.22 (0.14 to 0.4)	0.01 (-0.36 to 0.63)	7.07 (3.47 to 14.35)	7.39 (4.6 to 13.07)	0.04 (-0.37 to 0.73)
Democratic Republic of the Congo	1.65 (1.05 to 2.44)	1.59 (0.99 to 2.36)	-0.04 (-0.13 to 0.07)	0.11 (0.06 to 0.2)	0.08 (0.03 to 0.16)	-0.31 (-0.64 to 0.25)	3.48 (1.91 to 6.04)	2.46 (1.1 to 4.97)	-0.29 (-0.61 to 0.27)
Denmark	9.33 (6.42 to 12.63)	6.49 (4.46 to 8.8)	-0.3 (-0.38 to -0.22)	0.76 (0.57 to 0.97)	0.45 (0.33 to 0.55)	-0.41 (-0.57 to -0.23)	24.03 (18.14 to 29.98)	13.58 (10.13 to 16.36)	-0.43 (-0.58 to -0.27)
Djibouti	1.64 (1.04 to 2.42)	1.87 (1.17 to 2.74)	0.14 (0.02 to 0.27)	0.04 (0.02 to 0.09)	0.03 (0.01 to 0.06)	-0.28 (-0.62 to 0.38)	1.58 (0.67 to 3.07)	1.21 (0.47 to 2.27)	-0.24 (-0.55 to 0.43)
Dominica	2.84 (2.08 to 3.79)	3.05 (2.21 to 4.11)	0.07 (-0.07 to 0.23)	0.58 (0.4 to 0.89)	0.84 (0.52 to 1.19)	0.44 (-0.11 to 1.45)	17.64 (12.23 to 25.83)	24.85 (15.58 to 34.98)	0.41 (-0.13 to 1.39)
Dominican Republic	3.04 (2.15 to 4.28)	3.46 (2.42 to 4.9)	0.14 (-0.01 to 0.3)	0.23 (0.15 to 0.35)	0.45 (0.25 to 0.67)	0.98 (0.1 to 2.38)	7.57 (5.19 to 11.41)	14.69 (8.35 to 21.45)	0.94 (0.06 to 2.32)
Ecuador	0.32 (0.2 to 0.46)	0.4 (0.28 to 0.56)	0.27 (0.1 to 0.51)	0.08 (0.05 to 0.12)	0.04 (0.03 to 0.06)	-0.47 (-0.68 to -0.12)	2.06 (1.41 to 3.02)	1.07 (0.77 to 1.49)	-0.48 (-0.66 to -0.15)
Egypt	2.43 (1.57 to 3.79)	2.79 (1.79 to 4.35)	0.15 (-0.04 to 0.35)	0.3 (0.14 to 0.53)	0.18 (0.08 to 0.32)	-0.41 (-0.7 to 0.03)	7.89 (4.08 to 12.83)	5.23 (2.71 to 8.89)	-0.34 (-0.63 to 0.1)
El Salvador	0.3 (0.2 to 0.41)	0.36 (0.25 to 0.49)	0.21 (0.07 to 0.39)	0.06 (0.04 to 0.08)	0.02 (0.02 to 0.03)	-0.57 (-0.73 to -0.31)	1.6 (1.03 to 2.18)	0.74 (0.49 to 1.06)	-0.53 (-0.71 to -0.28)
Equatorial Guinea	1.41 (0.9 to 2.09)	1.6 (1.03 to 2.34)	0.14 (0.01 to 0.29)	0.18 (0.08 to 0.33)	0.05 (0.03 to 0.1)	-0.7 (-0.88 to -0.2)	5.7 (2.34 to 10.88)	1.85 (0.94 to 3.39)	-0.68 (-0.86 to -0.14)
Eritrea	1.3 (0.85 to 1.88)	1.45 (0.92 to 2.12)	0.11 (0 to 0.24)	0.05 (0.02 to 0.13)	0.03 (0.01 to 0.06)	-0.43 (-0.68 to 0.06)	1.92 (0.85 to 4.27)	1.17 (0.61 to 2.15)	-0.39 (-0.64 to 0.11)
Estonia	23.57 (16.13 to 33.72)	29 (19.12 to 42.31)	0.23 (0.06 to 0.42)	3.95 (2.76 to 5.46)	4.37 (2.99 to 5.89)	0.11 (-0.28 to 0.61)	143.13 (99.8 to 193.92)	142.51 (99.48 to 190.51)	0 (-0.35 to 0.5)

Eswatini	0.54 (0.37 to 0.76)	0.52 (0.35 to 0.72)	-0.05 (-0.14 to 0.06)	0.05 (0.03 to 0.08)	0.04 (0.02 to 0.06)	-0.28 (-0.54 to 0.13)	1.48 (0.99 to 2.2)	1.15 (0.69 to 1.89)	-0.23 (-0.53 to 0.26)
Ethiopia	1.94 (1.41 to 2.52)	2.22 (1.63 to 2.88)	0.15 (0.09 to 0.21)	0.05 (0.02 to 0.13)	0.02 (0.01 to 0.04)	-0.56 (-0.84 to 0.2)	1.78 (0.77 to 4.34)	0.87 (0.41 to 1.53)	-0.51 (-0.78 to 0.18)
Fiji	0.28 (0.2 to 0.38)	0.28 (0.21 to 0.38)	0.01 (-0.09 to 0.12)	0.05 (0.04 to 0.08)	0.04 (0.02 to 0.05)	-0.35 (-0.56 to 0)	1.72 (1.22 to 2.47)	1.17 (0.8 to 1.65)	-0.32 (-0.54 to 0.04)
Finland	40.61 (29.62 to 52.27)	32.95 (24.8 to 42.06)	-0.19 (-0.32 to -0.03)	3.67 (2.58 to 4.65)	2.45 (1.78 to 2.82)	-0.33 (-0.49 to -0.13)	126.67 (83.85 to 162.91)	79.49 (55.76 to 91.26)	-0.37 (-0.51 to -0.18)
France	28.85 (21.64 to 37.05)	15.46 (11.59 to 19.74)	-0.46 (-0.53 to -0.39)	1.14 (0.89 to 1.36)	0.43 (0.28 to 0.6)	-0.62 (-0.78 to -0.5)	33.06 (26.96 to 38.07)	13.67 (9.21 to 18.15)	-0.59 (-0.74 to -0.46)
Gabon	1.61 (1.03 to 2.41)	1.64 (1.05 to 2.45)	0.02 (-0.08 to 0.12)	0.14 (0.06 to 0.28)	0.07 (0.04 to 0.11)	-0.5 (-0.73 to 0.04)	4.67 (2.08 to 9.19)	2.42 (1.43 to 3.86)	-0.48 (-0.72 to 0.04)
Gambia	5.69 (3.48 to 8.86)	5.59 (3.41 to 8.58)	-0.02 (-0.14 to 0.11)	0.43 (0.22 to 0.74)	0.18 (0.1 to 0.3)	-0.58 (-0.78 to -0.19)	13.8 (7.19 to 23.79)	5.98 (3.52 to 9.8)	-0.57 (-0.77 to -0.17)
Georgia	0.81 (0.6 to 1.06)	0.81 (0.58 to 1.09)	0 (-0.12 to 0.12)	0.16 (0.08 to 0.24)	0.24 (0.15 to 0.47)	0.43 (-0.16 to 2.69)	4.94 (2.64 to 7.13)	8.84 (5.42 to 16.84)	0.79 (0.04 to 3.07)
Germany	44.86 (33.85 to 59.01)	40.3 (29.86 to 52.33)	-0.1 (-0.22 to 0.03)	4.27 (3.14 to 5.41)	2.05 (1.64 to 2.41)	-0.52 (-0.65 to -0.38)	117.25 (85.28 to 144.19)	54.94 (44.4 to 63.48)	-0.53 (-0.65 to -0.4)
Ghana	5.86 (3.88 to 8.46)	6.51 (3.67 to 10.85)	0.11 (-0.21 to 0.55)	0.67 (0.41 to 1.05)	0.64 (0.36 to 1)	-0.05 (-0.48 to 0.73)	21.71 (13.29 to 33.28)	21.27 (12.37 to 32.48)	-0.02 (-0.45 to 0.72)
Greece	1.52 (1.16 to 1.95)	1.18 (0.89 to 1.5)	-0.22 (-0.3 to -0.14)	0.12 (0.08 to 0.18)	0.05 (0.04 to 0.1)	-0.53 (-0.66 to -0.3)	3.74 (2.72 to 5.59)	1.9 (1.45 to 3.51)	-0.49 (-0.62 to -0.23)
Greenland	26.94 (20.48 to 35.17)	20.46 (15.43 to 26.66)	-0.24 (-0.33 to -0.14)	1.39 (0.94 to 2)	1.2 (0.73 to 1.66)	-0.14 (-0.48 to 0.37)	48.74 (34.05 to 68.7)	39.1 (24.7 to 54.01)	-0.2 (-0.49 to 0.26)
Grenada	9.49 (6.84 to 12.96)	10.56 (7.44 to 14.26)	0.11 (-0.03 to 0.26)	1.5 (1.08 to 2.21)	1.98 (1.19 to 2.48)	0.32 (-0.32 to 1.07)	46.28 (33.55 to 69.98)	59.44 (36.71 to 75.26)	0.28 (-0.34 to 1)

Guam	2.95 (2.1 to 4.12)	3.09 (2.2 to 4.32)	0.05 (-0.08 to 0.18)	0.62 (0.42 to 0.85)	0.11 (0.08 to 0.19)	-0.82 (-0.88 to -0.7)	15.97 (11.27 to 21.55)	3.7 (2.57 to 6.03)	-0.77 (-0.84 to -0.64)
Guatemala	0.58 (0.36 to 0.88)	0.79 (0.54 to 1.15)	0.36 (0.16 to 0.64)	0.15 (0.09 to 0.23)	0.06 (0.03 to 0.08)	-0.59 (-0.77 to -0.24)	3.66 (2.24 to 5.58)	1.56 (1 to 2.1)	-0.57 (-0.76 to -0.24)
Guinea	4.13 (2.87 to 5.51)	4.6 (2.84 to 6.8)	0.11 (-0.18 to 0.4)	0.36 (0.17 to 0.67)	0.17 (0.09 to 0.29)	-0.53 (-0.75 to -0.14)	11.51 (5.46 to 21.51)	5.64 (3.23 to 9.61)	-0.51 (-0.73 to -0.12)
Guinea-Bissau	5.01 (3.03 to 7.78)	5.32 (3.2 to 8.22)	0.06 (-0.07 to 0.21)	0.66 (0.25 to 1.4)	0.23 (0.12 to 0.41)	-0.65 (-0.83 to -0.25)	21.04 (8.07 to 43.75)	7.56 (4.33 to 12.97)	-0.64 (-0.82 to -0.22)
Guyana	6.75 (4.9 to 9.34)	5.84 (4.16 to 8.15)	-0.13 (-0.26 to 0)	3 (2.36 to 3.73)	2.21 (1.39 to 3.06)	-0.26 (-0.53 to 0.09)	94.41 (73.85 to 116.74)	69.72 (44.2 to 96.71)	-0.26 (-0.52 to 0.09)
Haiti	5.22 (3.64 to 7.33)	5.6 (3.94 to 7.73)	0.07 (-0.06 to 0.22)	2.64 (1.13 to 4.64)	1.62 (0.86 to 2.82)	-0.39 (-0.63 to 0.07)	82.09 (34.94 to 137.38)	49.59 (25.33 to 85.14)	-0.4 (-0.62 to 0.02)
Honduras	1.97 (1.21 to 3.02)	2.56 (1.73 to 3.68)	0.3 (0.1 to 0.62)	0.57 (0.28 to 0.85)	0.43 (0.21 to 0.62)	-0.26 (-0.5 to 0.16)	16.43 (8.32 to 24.31)	11.44 (5.47 to 17.35)	-0.3 (-0.54 to 0.1)
Hungary	36.43 (25.41 to 49.77)	38.71 (25.01 to 54.38)	0.06 (-0.1 to 0.22)	6.52 (5.61 to 8.4)	7.62 (5.47 to 9.82)	0.17 (-0.29 to 0.55)	188.95 (157.37 to 236.89)	227.42 (159.1 to 292.09)	0.2 (-0.25 to 0.59)
Iceland	5.54 (4.04 to 7.3)	4.24 (3.29 to 5.46)	-0.24 (-0.34 to -0.09)	0.32 (0.25 to 0.41)	0.14 (0.11 to 0.18)	-0.56 (-0.68 to -0.44)	8.64 (6.77 to 10.72)	3.95 (3.15 to 4.88)	-0.54 (-0.65 to -0.42)
India	0.72 (0.49 to 1.02)	0.73 (0.5 to 1.03)	0.02 (-0.01 to 0.04)	0.22 (0.1 to 0.38)	0.14 (0.1 to 0.19)	-0.37 (-0.56 to 0.03)	5.85 (2.7 to 9.73)	3.82 (2.65 to 5.35)	-0.35 (-0.52 to 0.05)
Indonesia	1.53 (1.18 to 2)	1.72 (1.32 to 2.25)	0.12 (0.08 to 0.17)	0.16 (0.09 to 0.27)	0.14 (0.08 to 0.25)	-0.1 (-0.47 to 0.46)	4.77 (2.75 to 7.57)	4.17 (2.35 to 7.69)	-0.13 (-0.49 to 0.43)
Iran (Islamic Republic of)	2.3 (1.67 to 3.08)	2.28 (1.67 to 3.05)	-0.01 (-0.05 to 0.03)	0.21 (0.15 to 0.31)	0.12 (0.07 to 0.16)	-0.45 (-0.68 to -0.13)	5.6 (4.06 to 8.56)	3.57 (2.44 to 4.66)	-0.36 (-0.6 to -0.05)
Iraq	0.74 (0.57 to 0.98)	0.72 (0.54 to 0.94)	-0.03 (-0.12 to 0.07)	0.14 (0.07 to 0.29)	0.05 (0.03 to 0.07)	-0.68 (-0.84 to -0.32)	3.08 (1.66 to 5.69)	1.17 (0.83 to 1.66)	-0.62 (-0.8 to -0.25)

Ireland	17.81 (13.79 to 22.74)	13.36 (10.11 to 17.12)	-0.25 (-0.33 to -0.17)	1.18 (0.99 to 1.62)	0.69 (0.46 to 0.95)	-0.41 (-0.68 to -0.21)	34.95 (29.8 to 45.81)	20.23 (14.25 to 27.31)	-0.42 (-0.66 to -0.24)
Israel	1.9 (1.42 to 2.49)	1.45 (1.08 to 1.91)	-0.23 (-0.32 to -0.14)	0.2 (0.13 to 0.27)	0.05 (0.03 to 0.06)	-0.78 (-0.89 to -0.67)	6.15 (4.14 to 8.05)	1.51 (1.04 to 2.04)	-0.75 (-0.86 to -0.65)
Italy	9.63 (6.93 to 12.89)	10.62 (7.98 to 13.9)	0.1 (-0.02 to 0.26)	0.95 (0.7 to 1.79)	0.09 (0.05 to 0.16)	-0.9 (-0.97 to -0.84)	24.22 (19.85 to 37.79)	3.77 (2.44 to 5.69)	-0.84 (-0.93 to -0.78)
Jamaica	3.37 (2.44 to 4.52)	4.07 (2.97 to 5.43)	0.21 (0.07 to 0.37)	0.23 (0.15 to 0.37)	0.51 (0.35 to 0.74)	1.24 (0.36 to 2.62)	7.18 (4.9 to 11.49)	16.06 (11.2 to 23.23)	1.24 (0.38 to 2.6)
Japan	8.07 (5.83 to 11.3)	7.64 (5.8 to 10.03)	-0.05 (-0.16 to 0.08)	0.26 (0.2 to 0.32)	0.12 (0.08 to 0.16)	-0.53 (-0.68 to -0.41)	9.61 (7.54 to 11.46)	4.83 (3.43 to 6.08)	-0.5 (-0.63 to -0.39)
Jordan	0.79 (0.58 to 1.03)	0.83 (0.62 to 1.07)	0.05 (-0.04 to 0.16)	0.09 (0.05 to 0.14)	0.03 (0.02 to 0.04)	-0.65 (-0.79 to -0.34)	2.29 (1.33 to 3.37)	0.99 (0.73 to 1.33)	-0.57 (-0.73 to -0.22)
Kazakhstan	2.58 (1.84 to 3.55)	2.6 (1.8 to 3.71)	0.01 (-0.11 to 0.15)	0.22 (0.08 to 0.8)	1.08 (0.38 to 2.04)	3.9 (-0.22 to 12.43)	8.45 (3.33 to 29.12)	39.3 (14.85 to 73.51)	3.65 (-0.2 to 11.06)
Kenya	1.36 (0.88 to 1.95)	1.32 (0.85 to 1.9)	-0.03 (-0.06 to -0.01)	0.03 (0.01 to 0.06)	0.03 (0.01 to 0.05)	-0.14 (-0.34 to 0.18)	1.12 (0.52 to 1.99)	1 (0.5 to 1.65)	-0.1 (-0.3 to 0.2)
Kiribati	1.2 (0.83 to 1.7)	1.26 (0.86 to 1.8)	0.05 (-0.07 to 0.17)	0.3 (0.17 to 0.43)	0.23 (0.11 to 0.35)	-0.22 (-0.48 to 0.17)	9.11 (4.78 to 13.4)	7.09 (3.34 to 10.91)	-0.22 (-0.5 to 0.19)
Kuwait	1.07 (0.79 to 1.41)	1.08 (0.8 to 1.43)	0.01 (-0.08 to 0.11)	0.08 (0.06 to 0.12)	0.04 (0.03 to 0.06)	-0.47 (-0.69 to -0.19)	2.3 (1.82 to 3.13)	1.23 (0.87 to 1.61)	-0.47 (-0.67 to -0.22)
Kyrgyzstan	17.52 (11.83 to 25.38)	20.23 (13.95 to 28.93)	0.15 (0.03 to 0.3)	4.11 (2.91 to 4.81)	6.5 (3.29 to 8.1)	0.58 (-0.02 to 0.95)	155.8 (107.85 to 181.96)	241.44 (117.45 to 301.23)	0.55 (-0.03 to 0.92)
Lao People's Democratic Republic	1.19 (0.88 to 1.57)	1.31 (0.99 to 1.73)	0.1 (0 to 0.23)	0.28 (0.1 to 0.56)	0.16 (0.1 to 0.26)	-0.43 (-0.7 to 0.37)	8.29 (2.86 to 16.94)	4.95 (2.85 to 8.8)	-0.4 (-0.69 to 0.51)
Latvia	38.05 (26.37 to 53.08)	62.23 (42.04 to 88.32)	0.64 (0.36 to 0.94)	2.98 (2.26 to 4.54)	16.31 (10.95 to 21.08)	4.47 (1.8 to 6.92)	118.78 (90.48 to 175.31)	629.33 (428.39 to 813.26)	4.3 (1.82 to 6.58)

Lebanon	2.82 (2.02 to 3.86)	2.93 (2.13 to 3.99)	0.04 (-0.07 to 0.17)	0.27 (0.16 to 0.39)	0.12 (0.05 to 0.2)	-0.53 (-0.8 to -0.16)	7.27 (4.51 to 10.66)	3.96 (1.78 to 6.29)	-0.46 (-0.72 to -0.04)
Lesotho	0.5 (0.34 to 0.69)	0.5 (0.34 to 0.7)	0.01 (-0.08 to 0.11)	0.05 (0.03 to 0.09)	0.05 (0.03 to 0.07)	-0.09 (-0.44 to 0.62)	1.44 (0.82 to 2.47)	1.36 (0.84 to 2.25)	-0.05 (-0.43 to 0.86)
Liberia	6.01 (3.64 to 9.32)	6.6 (3.98 to 10.26)	0.1 (-0.03 to 0.23)	0.51 (0.27 to 0.85)	0.16 (0.07 to 0.32)	-0.68 (-0.84 to -0.33)	16.39 (9.06 to 27.8)	5.52 (2.68 to 10.51)	-0.66 (-0.82 to -0.32)
Libya	1.91 (1.38 to 2.69)	2.16 (1.55 to 3.01)	0.13 (0.01 to 0.28)	0.15 (0.07 to 0.26)	0.1 (0.04 to 0.15)	-0.37 (-0.65 to 0.14)	4.3 (2.32 to 7.05)	3 (1.51 to 4.56)	-0.3 (-0.59 to 0.18)
Lithuania	18.21 (12.18 to 26.57)	20.66 (13.69 to 30.27)	0.13 (0 to 0.27)	3.17 (2.54 to 4.05)	4.74 (3.48 to 6.23)	0.5 (0.06 to 1.01)	121.62 (97.74 to 153.48)	178.4 (131.77 to 232.56)	0.47 (0.05 to 0.97)
Luxembourg	20.16 (14.77 to 26.97)	19.19 (15.15 to 24.37)	-0.05 (-0.21 to 0.16)	1.27 (0.95 to 1.55)	0.5 (0.31 to 0.68)	-0.61 (-0.78 to -0.43)	34.68 (26.67 to 41.74)	14.09 (9.04 to 19.17)	-0.59 (-0.76 to -0.4)
Madagascar	1.8 (1.15 to 2.63)	1.93 (1.24 to 2.81)	0.07 (-0.04 to 0.19)	0.07 (0.04 to 0.13)	0.05 (0.02 to 0.1)	-0.27 (-0.58 to 0.22)	2.76 (1.48 to 4.84)	2.04 (0.88 to 3.62)	-0.26 (-0.56 to 0.24)
Malawi	2.73 (1.7 to 4.03)	2.93 (1.88 to 4.3)	0.07 (-0.04 to 0.21)	0.06 (0.03 to 0.12)	0.05 (0.02 to 0.09)	-0.23 (-0.58 to 0.29)	2.16 (1.17 to 3.92)	1.78 (0.8 to 3.19)	-0.18 (-0.51 to 0.32)
Malaysia	0.91 (0.68 to 1.22)	1.05 (0.78 to 1.39)	0.16 (0 to 0.32)	0.07 (0.05 to 0.1)	0.03 (0.02 to 0.04)	-0.58 (-0.74 to -0.34)	1.96 (1.43 to 2.93)	1 (0.71 to 1.38)	-0.49 (-0.65 to -0.24)
Maldives	1.06 (0.78 to 1.41)	1.11 (0.82 to 1.5)	0.05 (-0.07 to 0.17)	0.12 (0.04 to 0.21)	0.03 (0.02 to 0.05)	-0.71 (-0.87 to -0.12)	3.64 (1.43 to 6.45)	1.2 (0.64 to 1.7)	-0.67 (-0.85 to -0.07)
Mali	3.46 (2.09 to 5.36)	3.62 (2.19 to 5.69)	0.05 (-0.07 to 0.18)	0.31 (0.17 to 0.58)	0.12 (0.06 to 0.22)	-0.61 (-0.78 to -0.24)	9.62 (5.45 to 17.86)	3.98 (2.06 to 6.88)	-0.59 (-0.76 to -0.25)
Malta	5.62 (4.18 to 7.31)	5.34 (3.92 to 6.9)	-0.05 (-0.2 to 0.15)	0.43 (0.33 to 0.53)	0.19 (0.12 to 0.25)	-0.55 (-0.72 to -0.39)	11.34 (8.9 to 13.74)	5.51 (3.59 to 7.16)	-0.51 (-0.68 to -0.36)
Marshall Islands	1.45 (1.04 to 1.98)	1.46 (1.04 to 2.01)	0.01 (-0.1 to 0.13)	0.43 (0.21 to 0.79)	0.25 (0.13 to 0.4)	-0.43 (-0.63 to -0.12)	11.33 (5.39 to 19.53)	6.92 (3.34 to 11.26)	-0.39 (-0.6 to -0.08)

Mauritania	4.11 (2.49 to 6.44)	4.32 (2.58 to 6.82)	0.05 (-0.08 to 0.19)	0.4 (0.21 to 0.7)	0.1 (0.04 to 0.19)	-0.75 (-0.89 to -0.45)	12.83 (6.85 to 22.41)	3.47 (1.42 to 6.47)	-0.73 (-0.87 to -0.43)
Mauritius	3.46 (2.6 to 4.52)	3.22 (2.43 to 4.18)	-0.07 (-0.16 to 0.03)	0.37 (0.27 to 0.52)	0.21 (0.15 to 0.29)	-0.44 (-0.64 to -0.03)	14.23 (10.11 to 19.88)	8.94 (6.19 to 12.45)	-0.37 (-0.6 to 0.13)
Mexico	1.53 (1.04 to 2.19)	1.44 (1 to 2.03)	-0.06 (-0.1 to -0.01)	0.16 (0.13 to 0.21)	0.11 (0.09 to 0.15)	-0.29 (-0.53 to 0)	4.74 (3.87 to 6.09)	3.66 (2.77 to 5.06)	-0.23 (-0.47 to 0.12)
Micronesia (Federated States of)	1.32 (0.94 to 1.82)	1.32 (0.94 to 1.83)	0 (-0.11 to 0.12)	0.42 (0.19 to 0.87)	0.23 (0.11 to 0.4)	-0.45 (-0.7 to -0.09)	11.59 (4.99 to 21.38)	6.64 (2.88 to 11.97)	-0.43 (-0.67 to -0.08)
Monaco	12.39 (8.99 to 16.15)	8.69 (6.38 to 11.41)	-0.3 (-0.37 to -0.22)	0.85 (0.55 to 1.2)	0.43 (0.29 to 0.6)	-0.5 (-0.68 to -0.2)	24.52 (16.5 to 34.08)	12.44 (8.54 to 17.63)	-0.49 (-0.67 to -0.2)
Mongolia	2.9 (1.86 to 4.28)	3.07 (1.96 to 4.65)	0.06 (-0.11 to 0.24)	0.7 (0.37 to 1.38)	1.01 (0.6 to 1.89)	0.44 (-0.14 to 1.57)	27.81 (14.42 to 55.17)	41.39 (24.51 to 77.57)	0.49 (-0.13 to 1.71)
Montenegro	95.29 (69.05 to 127.66)	81.6 (59.62 to 109.61)	-0.14 (-0.24 to -0.04)	13.38 (5.28 to 23.04)	9.15 (4.61 to 14.02)	-0.32 (-0.54 to 0.1)	293.22 (145.7 to 459.95)	229.01 (122.03 to 342.65)	-0.22 (-0.46 to 0.14)
Morocco	2.17 (1.54 to 3.04)	2.26 (1.63 to 3.09)	0.04 (-0.06 to 0.16)	0.42 (0.17 to 0.89)	0.19 (0.1 to 0.27)	-0.56 (-0.79 to -0.12)	10.31 (4.72 to 19.71)	4.73 (2.63 to 7.01)	-0.54 (-0.77 to -0.14)
Mozambique	2.94 (1.86 to 4.37)	3.54 (2.32 to 5.05)	0.2 (0.07 to 0.39)	0.06 (0.02 to 0.13)	0.06 (0.02 to 0.12)	-0.09 (-0.43 to 0.65)	2.18 (0.93 to 4.46)	2.19 (1.15 to 4.33)	0 (-0.35 to 0.63)
Myanmar	1.26 (0.94 to 1.68)	1.3 (0.96 to 1.73)	0.03 (-0.08 to 0.15)	0.23 (0.1 to 0.43)	0.12 (0.08 to 0.2)	-0.47 (-0.69 to 0.07)	7.19 (3.05 to 13.84)	3.92 (2.46 to 6.67)	-0.45 (-0.68 to 0.15)
Namibia	0.54 (0.37 to 0.74)	0.52 (0.36 to 0.72)	-0.04 (-0.13 to 0.06)	0.05 (0.03 to 0.08)	0.04 (0.02 to 0.06)	-0.25 (-0.56 to 0.26)	1.37 (0.85 to 2.14)	1.08 (0.72 to 1.69)	-0.21 (-0.54 to 0.37)
Nauru	1.46 (1.05 to 1.99)	1.43 (1.01 to 1.94)	-0.02 (-0.12 to 0.09)	0.34 (0.17 to 0.64)	0.22 (0.11 to 0.42)	-0.34 (-0.57 to 0.07)	10.17 (4.81 to 20.03)	6.79 (3.26 to 13.74)	-0.33 (-0.57 to 0.06)
Nepal	0.52 (0.36 to 0.73)	0.51 (0.35 to 0.72)	-0.02 (-0.12 to 0.1)	0.21 (0.1 to 0.38)	0.16 (0.09 to 0.26)	-0.25 (-0.58 to 0.37)	5.6 (2.73 to 9.63)	3.98 (2.29 to 6.5)	-0.29 (-0.61 to 0.3)

Netherlands	21.28 (16.47 to 26.51)	15.84 (12.02 to 20.6)	-0.26 (-0.37 to -0.11)	2.07 (1.57 to 2.55)	0.5 (0.3 to 0.75)	-0.76 (-0.88 to -0.62)	50.82 (41.13 to 58.83)	14.85 (9.13 to 21.63)	-0.71 (-0.84 to -0.57)
New Zealand	33.89 (23.93 to 48.43)	26.88 (19.58 to 36.02)	-0.21 (-0.32 to -0.06)	2.13 (1.48 to 2.91)	1.2 (0.81 to 1.4)	-0.44 (-0.59 to -0.31)	68.62 (48.23 to 95.45)	37.58 (25.91 to 43.32)	-0.45 (-0.59 to -0.34)
Nicaragua	0.65 (0.44 to 0.93)	0.89 (0.65 to 1.2)	0.37 (0.19 to 0.65)	0.13 (0.09 to 0.18)	0.08 (0.05 to 0.11)	-0.38 (-0.59 to -0.09)	3.71 (2.59 to 5.01)	1.9 (1.36 to 2.53)	-0.49 (-0.65 to -0.25)
Niger	3.9 (2.37 to 6.09)	3.97 (2.39 to 6.18)	0.02 (-0.12 to 0.16)	0.42 (0.21 to 0.82)	0.15 (0.07 to 0.28)	-0.65 (-0.82 to -0.28)	13.09 (6.85 to 25.06)	4.79 (2.34 to 9.08)	-0.63 (-0.8 to -0.28)
Nigeria	5.33 (3.38 to 7.82)	5.02 (3.24 to 7.36)	-0.06 (-0.1 to -0.01)	0.63 (0.33 to 1.06)	0.16 (0.09 to 0.25)	-0.75 (-0.87 to -0.51)	21.34 (11.1 to 35.29)	5.52 (3.25 to 8.66)	-0.74 (-0.86 to -0.5)
Niue	1.52 (1.1 to 2.12)	1.6 (1.13 to 2.21)	0.06 (-0.05 to 0.18)	0.23 (0.15 to 0.35)	0.13 (0.08 to 0.2)	-0.43 (-0.63 to -0.11)	6.53 (4.15 to 9.88)	3.84 (2.33 to 5.86)	-0.41 (-0.63 to -0.05)
North Macedonia	11.21 (8.26 to 14.79)	8.61 (6.31 to 11.5)	-0.23 (-0.32 to -0.14)	4.97 (3.21 to 7.01)	0.6 (0.35 to 1.07)	-0.88 (-0.93 to -0.75)	88.59 (64.29 to 118)	16.54 (9.05 to 26.44)	-0.81 (-0.89 to -0.69)
Northern Mariana Islands	6.06 (4.27 to 8.23)	5.74 (4.07 to 7.86)	-0.05 (-0.18 to 0.09)	0.94 (0.62 to 1.32)	0.2 (0.14 to 0.34)	-0.78 (-0.86 to -0.67)	26.35 (17.3 to 37.26)	5.95 (4.15 to 9.54)	-0.77 (-0.85 to -0.65)
Norway	12.04 (8.68 to 16.28)	12.48 (9.3 to 16.61)	0.04 (-0.06 to 0.15)	0.59 (0.46 to 0.68)	0.21 (0.16 to 0.29)	-0.65 (-0.71 to -0.5)	20.84 (16.61 to 23.29)	7.67 (6.13 to 9.91)	-0.63 (-0.69 to -0.49)
Oman	0.92 (0.69 to 1.2)	0.93 (0.67 to 1.31)	0.01 (-0.15 to 0.2)	0.18 (0.08 to 0.33)	0.06 (0.03 to 0.1)	-0.65 (-0.85 to -0.02)	4.68 (2.24 to 8.62)	1.79 (0.95 to 3.01)	-0.62 (-0.84 to 0.09)
Pakistan	0.68 (0.46 to 0.96)	0.69 (0.47 to 0.98)	0.01 (-0.05 to 0.09)	0.21 (0.1 to 0.35)	0.18 (0.1 to 0.26)	-0.17 (-0.4 to 0.23)	5.53 (2.52 to 9.19)	4.78 (2.79 to 7.05)	-0.14 (-0.38 to 0.27)
Palau	0.58 (0.42 to 0.81)	0.59 (0.42 to 0.8)	0.01 (-0.08 to 0.12)	0.07 (0.04 to 0.1)	0.05 (0.03 to 0.07)	-0.26 (-0.52 to 0.21)	2.24 (1.44 to 3.41)	1.79 (1.18 to 2.54)	-0.2 (-0.48 to 0.33)
Palestine	1.13 (0.85 to 1.48)	1.19 (0.89 to 1.56)	0.05 (-0.05 to 0.16)	0.33 (0.1 to 0.64)	0.08 (0.04 to 0.11)	-0.77 (-0.89 to -0.42)	5.97 (2.14 to 11.11)	1.73 (1.1 to 2.3)	-0.71 (-0.85 to -0.35)

Panama	0.65 (0.41 to 0.98)	0.79 (0.54 to 1.16)	0.22 (0.03 to 0.47)	0.09 (0.07 to 0.13)	0.07 (0.04 to 0.11)	-0.26 (-0.56 to 0.18)	3.27 (2.48 to 4.88)	2.63 (1.63 to 4.32)	-0.2 (-0.52 to 0.28)
Papua New Guinea	1.36 (0.95 to 1.9)	1.41 (0.99 to 1.96)	0.04 (-0.08 to 0.16)	0.31 (0.13 to 0.64)	0.2 (0.1 to 0.37)	-0.35 (-0.57 to 0.04)	8.23 (3.65 to 16.17)	5.55 (2.75 to 9.81)	-0.33 (-0.56 to 0.06)
Paraguay	1.6 (1 to 2.41)	2.01 (1.34 to 2.89)	0.26 (0.06 to 0.52)	0.26 (0.16 to 0.37)	0.16 (0.1 to 0.24)	-0.38 (-0.64 to 0.04)	8.81 (5.33 to 12.27)	5.59 (3.58 to 8.27)	-0.37 (-0.62 to 0.04)
Peru	0.82 (0.54 to 1.19)	1.02 (0.71 to 1.41)	0.25 (0.09 to 0.48)	0.1 (0.06 to 0.15)	0.04 (0.03 to 0.06)	-0.61 (-0.79 to -0.24)	2.64 (1.64 to 3.89)	1.18 (0.8 to 1.84)	-0.55 (-0.74 to -0.14)
Philippines	1.28 (0.99 to 1.67)	1.31 (1.01 to 1.72)	0.03 (0 to 0.06)	0.1 (0.06 to 0.13)	0.17 (0.1 to 0.23)	0.7 (0.09 to 1.36)	2.55 (1.84 to 4.03)	5.84 (3.54 to 8.19)	1.29 (0.12 to 2.22)
Poland	31.95 (23.22 to 44.23)	38.74 (29.08 to 52.33)	0.21 (0.07 to 0.39)	2.62 (1.98 to 3.18)	2.78 (1.89 to 3.65)	0.06 (-0.25 to 0.38)	64.01 (54.22 to 78.15)	85.69 (58.19 to 112.32)	0.34 (-0.08 to 0.77)
Portugal	9.04 (6.59 to 11.95)	6.27 (4.67 to 8.22)	-0.31 (-0.42 to -0.17)	0.43 (0.35 to 0.54)	0.34 (0.23 to 0.44)	-0.2 (-0.45 to 0.01)	13.35 (11.22 to 16.82)	11.11 (7.56 to 14.01)	-0.17 (-0.44 to 0.05)
Puerto Rico	7.7 (5.58 to 10.59)	6.69 (4.77 to 9.26)	-0.13 (-0.25 to 0)	1.06 (0.76 to 1.59)	0.35 (0.23 to 0.53)	-0.67 (-0.84 to -0.47)	31.38 (22.85 to 44.96)	11.39 (7.82 to 16.67)	-0.64 (-0.81 to -0.44)
Qatar	3.58 (2.82 to 4.49)	3.28 (2.45 to 4.32)	-0.08 (-0.25 to 0.1)	0.35 (0.17 to 0.73)	0.15 (0.08 to 0.25)	-0.56 (-0.82 to 0.06)	6.95 (4.06 to 12.1)	2.91 (1.71 to 4.48)	-0.58 (-0.8 to -0.13)
Republic of Korea	1.29 (0.95 to 1.69)	1.26 (0.95 to 1.65)	-0.02 (-0.12 to 0.11)	0.09 (0.06 to 0.15)	0.03 (0.02 to 0.04)	-0.7 (-0.83 to -0.44)	2.76 (1.7 to 4.48)	0.95 (0.69 to 1.24)	-0.66 (-0.8 to -0.41)
Republic of Moldova	14.5 (10.03 to 20.57)	27.21 (18.46 to 39.02)	0.88 (0.63 to 1.13)	0.32 (0.2 to 0.98)	4.21 (2.82 to 5.21)	12.12 (2.38 to 22.2)	12.19 (7.85 to 34.66)	147.14 (101.08 to 184.32)	11.07 (2.37 to 19.72)
Romania	13.83 (9.85 to 19.13)	12.24 (8.45 to 17.29)	-0.11 (-0.25 to 0.03)	3.4 (2.4 to 4.27)	2.05 (1.38 to 3.02)	-0.4 (-0.6 to -0.12)	119.46 (84.02 to 147.82)	67.57 (45.32 to 98.66)	-0.43 (-0.62 to -0.19)
Russian Federation	54.93 (39.62 to 74.81)	50.96 (36.4 to 70.75)	-0.07 (-0.12 to -0.04)	10.03 (7.69 to 15.81)	15.65 (11.7 to 19.17)	0.56 (-0.17 to 1.22)	359.48 (265.49 to 571.29)	602.19 (452.33 to 738.45)	0.68 (-0.1 to 1.46)

Rwanda	2.68 (1.72 to 3.88)	2.78 (1.79 to 4.15)	0.04 (-0.08 to 0.16)	0.13 (0.06 to 0.32)	0.04 (0.01 to 0.09)	-0.7 (-0.89 to -0.28)	4.64 (2.16 to 10.56)	1.5 (0.65 to 2.84)	-0.68 (-0.87 to -0.24)
Saint Kitts and Nevis	7.79 (5.71 to 10.59)	8.36 (6.16 to 11.38)	0.07 (-0.04 to 0.2)	1.39 (1.03 to 1.97)	1.6 (1.05 to 2.09)	0.15 (-0.32 to 0.8)	43.69 (32.36 to 63.31)	46.61 (30.9 to 63.26)	0.07 (-0.39 to 0.68)
Saint Lucia	16.35 (11.79 to 21.98)	17.86 (12.51 to 23.98)	0.09 (-0.05 to 0.25)	2.26 (1.66 to 3.26)	2.63 (1.85 to 3.33)	0.16 (-0.29 to 0.73)	69.08 (51.59 to 97.09)	80.82 (58.24 to 101.97)	0.17 (-0.27 to 0.75)
Saint Vincent and the Grenadines	5.55 (3.87 to 7.6)	6.81 (4.7 to 9.45)	0.23 (0.05 to 0.41)	0.55 (0.44 to 0.79)	1.27 (0.91 to 1.57)	1.32 (0.49 to 2.22)	17.19 (13.9 to 24.32)	38.65 (27.75 to 48.15)	1.25 (0.44 to 2.09)
Samoa	1.64 (1.17 to 2.26)	1.67 (1.18 to 2.29)	0.02 (-0.1 to 0.13)	0.27 (0.17 to 0.44)	0.17 (0.1 to 0.27)	-0.39 (-0.59 to -0.08)	7.24 (4.56 to 11.3)	4.75 (2.9 to 7.44)	-0.34 (-0.56 to -0.02)
San Marino	30.6 (22.62 to 40.79)	22.28 (16.3 to 29.48)	-0.27 (-0.36 to -0.18)	1.27 (0.86 to 1.69)	0.89 (0.52 to 1.4)	-0.3 (-0.6 to 0.22)	33.72 (24.42 to 43.82)	23.56 (14.38 to 36.82)	-0.3 (-0.59 to 0.2)
Sao Tome and Principe	6.19 (3.61 to 9.68)	6.94 (4.17 to 10.72)	0.12 (-0.02 to 0.29)	0.36 (0.18 to 0.6)	0.2 (0.11 to 0.33)	-0.45 (-0.7 to 0.02)	11.75 (6.23 to 19.26)	6.81 (3.77 to 11.22)	-0.42 (-0.68 to 0.04)
Saudi Arabia	1.35 (1 to 1.81)	1.52 (1.13 to 2.05)	0.13 (0 to 0.29)	0.28 (0.13 to 0.61)	0.1 (0.05 to 0.16)	-0.64 (-0.87 to -0.05)	5.29 (2.77 to 10.74)	2.2 (1.31 to 3.27)	-0.59 (-0.83 to -0.06)
Senegal	5.17 (3.17 to 7.86)	5.01 (3.1 to 7.49)	-0.03 (-0.15 to 0.11)	0.37 (0.19 to 0.62)	0.13 (0.07 to 0.23)	-0.65 (-0.81 to -0.37)	11.85 (6.46 to 19.85)	4.43 (2.35 to 7.68)	-0.63 (-0.78 to -0.36)
Serbia	8.16 (5.55 to 11.7)	5.71 (4.06 to 7.6)	-0.3 (-0.47 to -0.06)	0.76 (0.45 to 1.32)	1.14 (0.6 to 1.82)	0.49 (-0.1 to 1.58)	27.08 (16.53 to 42.28)	37.98 (19.31 to 60.43)	0.4 (-0.15 to 1.39)
Seychelles	5.74 (4.02 to 8.04)	5.51 (3.88 to 7.6)	-0.04 (-0.17 to 0.1)	0.96 (0.62 to 1.36)	0.62 (0.42 to 0.83)	-0.36 (-0.58 to -0.01)	31.26 (20.46 to 44.35)	20.33 (13.69 to 26.72)	-0.35 (-0.57 to -0.01)
Sierra Leone	7.59 (4.58 to 11.69)	8.02 (4.82 to 12.39)	0.06 (-0.07 to 0.21)	0.57 (0.31 to 0.97)	0.22 (0.11 to 0.39)	-0.62 (-0.8 to -0.26)	18.13 (10.19 to 30.48)	7.41 (3.85 to 13.05)	-0.59 (-0.78 to -0.24)
Singapore	5.78 (4.37 to 7.6)	4.57 (3.42 to 6.05)	-0.21 (-0.29 to -0.13)	0.68 (0.54 to 0.91)	0.1 (0.06 to 0.17)	-0.85 (-0.93 to -0.76)	22.32 (17.39 to 32.1)	3.63 (2.35 to 5.47)	-0.84 (-0.92 to -0.75)

Slovakia	12.61 (8.61 to 17.55)	19.91 (13.31 to 27.62)	0.58 (0.18 to 1.09)	0.74 (0.55 to 1.06)	1.65 (0.81 to 2.38)	1.24 (0.06 to 2.84)	27.07 (20.4 to 38.17)	55.83 (27.65 to 81.25)	1.06 (0.02 to 2.42)
Slovenia	25.16 (17.6 to 33.82)	16.42 (12.3 to 21.16)	-0.35 (-0.46 to -0.2)	13.03 (4.92 to 23.42)	0.18 (0.11 to 0.26)	-0.99 (-0.99 to -0.97)	245.28 (117.51 to 401.86)	3.97 (2.84 to 5.65)	-0.98 (-0.99 to -0.96)
Solomon Islands	1.16 (0.81 to 1.63)	1.15 (0.8 to 1.6)	-0.01 (-0.13 to 0.11)	0.44 (0.19 to 0.76)	0.27 (0.12 to 0.48)	-0.38 (-0.6 to -0.03)	12.84 (5.04 to 24.27)	7.9 (3.26 to 15.74)	-0.38 (-0.61 to -0.04)
Somalia	2.77 (1.75 to 4.13)	3.01 (1.92 to 4.37)	0.09 (-0.03 to 0.23)	0.08 (0.03 to 0.19)	0.06 (0.02 to 0.17)	-0.22 (-0.62 to 0.52)	2.69 (1.22 to 6.42)	2.1 (0.79 to 5.31)	-0.22 (-0.55 to 0.41)
South Africa	0.57 (0.41 to 0.77)	0.52 (0.37 to 0.69)	-0.1 (-0.14 to -0.06)	0.04 (0.03 to 0.05)	0.03 (0.02 to 0.04)	-0.27 (-0.44 to -0.1)	1.29 (1.02 to 1.67)	0.88 (0.64 to 1.22)	-0.32 (-0.5 to -0.11)
South Sudan	1.59 (1.02 to 2.36)	1.62 (1.04 to 2.38)	0.02 (-0.08 to 0.15)	0.04 (0.01 to 0.08)	0.02 (0.01 to 0.05)	-0.43 (-0.69 to 0.08)	1.44 (0.56 to 2.81)	0.87 (0.4 to 1.64)	-0.39 (-0.65 to 0.06)
Spain	11.58 (8.39 to 15.26)	8.06 (5.78 to 10.68)	-0.3 (-0.41 to -0.2)	0.89 (0.58 to 1.05)	0.31 (0.22 to 0.43)	-0.65 (-0.74 to -0.51)	21.23 (16.91 to 25.4)	9.33 (7.02 to 13.28)	-0.56 (-0.65 to -0.43)
Sri Lanka	8.95 (6.43 to 12.49)	9.29 (6.6 to 12.48)	0.04 (-0.1 to 0.2)	0.59 (0.36 to 0.88)	0.8 (0.38 to 1.33)	0.36 (-0.27 to 1.31)	20.16 (12.64 to 29.58)	26.77 (13.16 to 44.02)	0.33 (-0.26 to 1.24)
Sudan	1.98 (1.39 to 2.8)	2.15 (1.5 to 2.99)	0.09 (-0.03 to 0.22)	0.6 (0.19 to 1.26)	0.21 (0.11 to 0.35)	-0.65 (-0.83 to -0.25)	15.53 (5.41 to 31.5)	5.57 (3.02 to 9.21)	-0.64 (-0.82 to -0.19)
Suriname	1.57 (1.14 to 2.17)	1.69 (1.23 to 2.3)	0.08 (-0.08 to 0.23)	0.2 (0.14 to 0.28)	0.23 (0.14 to 0.32)	0.15 (-0.28 to 0.75)	6.43 (4.55 to 9.05)	7.4 (4.54 to 10.11)	0.15 (-0.27 to 0.75)
Sweden	35.45 (25.35 to 48.67)	34.11 (23.87 to 47.68)	-0.04 (-0.15 to 0.1)	0.84 (0.67 to 1.1)	0.72 (0.54 to 0.91)	-0.14 (-0.44 to 0.11)	29.55 (24.08 to 38.99)	23.68 (18.65 to 29.01)	-0.2 (-0.43 to 0)
Switzerland	22.35 (15.93 to 29.86)	14.77 (11.49 to 18.47)	-0.34 (-0.46 to -0.18)	1.74 (1.24 to 2.1)	0.49 (0.33 to 0.66)	-0.72 (-0.83 to -0.57)	47.67 (34.23 to 57.14)	13.56 (9.58 to 17.92)	-0.72 (-0.82 to -0.57)
Syrian Arab Republic	2.02 (1.44 to 2.8)	2.27 (1.62 to 3.13)	0.13 (0 to 0.27)	0.33 (0.18 to 0.53)	0.16 (0.07 to 0.27)	-0.51 (-0.76 to -0.13)	8.11 (4.67 to 12.41)	4.26 (2.07 to 6.98)	-0.48 (-0.72 to -0.1)

Taiwan (Province of China)	8.07 (6.04 to 10.48)	7.6 (5.84 to 9.6)	-0.06 (-0.18 to 0.08)	0.43 (0.35 to 0.53)	0.19 (0.13 to 0.26)	-0.56 (-0.72 to -0.39)	12.5 (10.66 to 15.69)	7.44 (5.13 to 10.32)	-0.4 (-0.61 to -0.17)
Tajikistan	0.15 (0.11 to 0.2)	0.15 (0.11 to 0.2)	0 (-0.11 to 0.11)	0.03 (0.02 to 0.05)	0.03 (0.02 to 0.05)	0.07 (-0.28 to 0.72)	1.01 (0.61 to 1.93)	0.99 (0.69 to 1.76)	-0.02 (-0.33 to 0.58)
Thailand	1.1 (0.79 to 1.49)	1.2 (0.87 to 1.66)	0.1 (0 to 0.23)	0.05 (0.03 to 0.1)	0.03 (0.02 to 0.04)	-0.48 (-0.7 to -0.03)	1.56 (0.97 to 2.72)	0.98 (0.68 to 1.46)	-0.37 (-0.62 to 0.1)
Timor-Leste	1.16 (0.88 to 1.54)	1.38 (1.05 to 1.81)	0.19 (0.05 to 0.34)	0.13 (0.07 to 0.25)	0.13 (0.07 to 0.23)	-0.02 (-0.48 to 0.83)	3.82 (1.9 to 7.56)	3.91 (2.01 to 7.43)	0.02 (-0.48 to 0.92)
Togo	4.83 (2.93 to 7.44)	4.79 (2.94 to 7.27)	-0.01 (-0.12 to 0.13)	0.43 (0.23 to 0.7)	0.17 (0.1 to 0.3)	-0.6 (-0.78 to -0.27)	13.74 (7.7 to 22.21)	5.79 (3.43 to 9.77)	-0.58 (-0.76 to -0.26)
Tokelau	1.61 (1.16 to 2.2)	1.76 (1.26 to 2.39)	0.1 (-0.01 to 0.23)	0.3 (0.15 to 0.5)	0.13 (0.07 to 0.19)	-0.58 (-0.74 to -0.25)	8.1 (4.25 to 13.57)	3.62 (2.08 to 5.61)	-0.55 (-0.73 to -0.19)
Tonga	1.7 (1.21 to 2.36)	1.7 (1.21 to 2.35)	0 (-0.1 to 0.14)	0.13 (0.07 to 0.21)	0.09 (0.05 to 0.15)	-0.3 (-0.53 to 0.1)	3.57 (2.11 to 5.69)	2.66 (1.52 to 4.29)	-0.25 (-0.51 to 0.16)
Trinidad and Tobago	4.62 (3.25 to 6.49)	4.61 (3.17 to 6.47)	0 (-0.15 to 0.17)	0.58 (0.38 to 1.01)	0.6 (0.39 to 0.94)	0.02 (-0.38 to 0.79)	17.13 (11.3 to 29.64)	18.07 (11.98 to 28.38)	0.06 (-0.36 to 0.82)
Tunisia	1.72 (1.24 to 2.3)	1.88 (1.39 to 2.56)	0.09 (-0.02 to 0.22)	0.19 (0.11 to 0.3)	0.1 (0.05 to 0.17)	-0.46 (-0.74 to -0.07)	4.72 (2.75 to 7.26)	2.93 (1.46 to 4.84)	-0.38 (-0.65 to 0.01)
Turkey	1.11 (0.82 to 1.49)	0.85 (0.69 to 1.06)	-0.23 (-0.37 to -0.06)	0.1 (0.05 to 0.16)	0.03 (0.02 to 0.05)	-0.68 (-0.82 to -0.3)	2.7 (1.46 to 4.31)	0.95 (0.68 to 1.37)	-0.65 (-0.8 to -0.32)
Turkmenistan	3.18 (2.28 to 4.47)	3.41 (2.48 to 4.77)	0.07 (-0.05 to 0.2)	1.18 (0.6 to 2.51)	1.51 (0.77 to 3.32)	0.28 (-0.27 to 1.15)	40.17 (20.3 to 85.32)	54.44 (27.86 to 120.51)	0.36 (-0.21 to 1.24)
Tuvalu	1.43 (1.03 to 1.98)	1.55 (1.09 to 2.15)	0.08 (-0.04 to 0.21)	0.42 (0.21 to 0.76)	0.19 (0.11 to 0.3)	-0.55 (-0.72 to -0.23)	11.52 (5.49 to 19.81)	5.47 (3.21 to 8.52)	-0.53 (-0.71 to -0.18)
Uganda	1.91 (1.17 to 2.91)	1.9 (1.17 to 2.91)	0 (-0.12 to 0.11)	0.07 (0.03 to 0.15)	0.04 (0.02 to 0.08)	-0.43 (-0.65 to -0.03)	2.54 (1.15 to 4.69)	1.54 (0.73 to 2.84)	-0.39 (-0.62 to 0.03)

Ukraine	54.17 (39.75 to 71.77)	50.82 (37.18 to 66.97)	-0.06 (-0.16 to 0.03)	12.21 (9.84 to 14.88)	16.24 (12.44 to 22.27)	0.33 (-0.05 to 0.87)	432.39 (345.47 to 522.6)	636.34 (483.52 to 875.78)	0.47 (0.05 to 1.07)
United Arab Emirates	2.08 (1.52 to 2.88)	2.08 (1.48 to 2.84)	0 (-0.11 to 0.13)	0.4 (0.13 to 0.97)	0.14 (0.04 to 0.3)	-0.66 (-0.82 to -0.32)	10.59 (3.55 to 24.43)	4.1 (1.51 to 8.2)	-0.61 (-0.78 to -0.26)
United Kingdom	10.07 (7.54 to 13.26)	8.04 (6.31 to 10.18)	-0.2 (-0.29 to -0.11)	0.6 (0.52 to 0.74)	0.49 (0.4 to 0.61)	-0.18 (-0.37 to -0.06)	21.06 (18.27 to 26.2)	17.16 (13.55 to 20.6)	-0.19 (-0.35 to -0.08)
United Republic of Tanzania	2.07 (1.34 to 3)	2.31 (1.49 to 3.3)	0.11 (0 to 0.25)	0.06 (0.02 to 0.12)	0.04 (0.01 to 0.08)	-0.26 (-0.6 to 0.37)	2.06 (0.99 to 3.76)	1.62 (0.65 to 2.86)	-0.21 (-0.54 to 0.37)
United States of America	43.53 (32.74 to 59.64)	40.33 (31.26 to 52.55)	-0.07 (-0.19 to 0.07)	2.13 (1.52 to 2.57)	1.13 (0.93 to 1.4)	-0.47 (-0.6 to -0.27)	67.36 (50.42 to 79.43)	36.05 (30.76 to 44.05)	-0.46 (-0.57 to -0.29)
United States Virgin Islands	13.88 (10.03 to 18.68)	13.18 (9.31 to 17.89)	-0.05 (-0.17 to 0.09)	2.8 (2.04 to 4.16)	3.04 (2 to 4.01)	0.08 (-0.3 to 0.63)	86.56 (63.92 to 127.44)	90.49 (59.46 to 122)	0.05 (-0.33 to 0.63)
Uruguay	23.92 (16.18 to 33.52)	19.37 (13.32 to 26.75)	-0.19 (-0.3 to -0.06)	2.3 (1.72 to 3.25)	1 (0.72 to 1.37)	-0.57 (-0.77 to -0.41)	62.42 (46.62 to 86.94)	28.07 (20.54 to 37.49)	-0.55 (-0.76 to -0.4)
Uzbekistan	0.08 (0.06 to 0.12)	0.09 (0.06 to 0.12)	0.07 (-0.05 to 0.19)	0.04 (0.01 to 0.07)	0.05 (0.04 to 0.07)	0.33 (-0.34 to 2.45)	1.09 (0.46 to 2.02)	1.39 (1.05 to 2.11)	0.27 (-0.3 to 1.82)
Vanuatu	1.71 (1.21 to 2.38)	1.64 (1.17 to 2.27)	-0.04 (-0.15 to 0.09)	0.38 (0.18 to 0.64)	0.25 (0.13 to 0.42)	-0.33 (-0.55 to 0.06)	10.27 (4.77 to 17.1)	7.22 (3.7 to 11.86)	-0.3 (-0.54 to 0.1)
Venezuela (Bolivarian Republic of)	1.63 (0.95 to 2.71)	1.69 (0.97 to 2.77)	0.04 (-0.18 to 0.33)	0.99 (0.63 to 1.53)	0.27 (0.18 to 0.39)	-0.72 (-0.83 to -0.56)	26.89 (17.22 to 37.56)	8.68 (5.76 to 12.44)	-0.68 (-0.8 to -0.5)
Viet Nam	1.65 (1.25 to 2.2)	2.1 (1.63 to 2.74)	0.27 (0.1 to 0.49)	0.14 (0.07 to 0.26)	0.16 (0.09 to 0.28)	0.09 (-0.37 to 0.96)	4.2 (2.22 to 7.96)	5.1 (2.89 to 9.49)	0.22 (-0.3 to 1.14)
Yemen	1.52 (1.08 to 2.07)	1.6 (1.13 to 2.22)	0.05 (-0.05 to 0.16)	0.57 (0.17 to 1.27)	0.23 (0.11 to 0.37)	-0.6 (-0.82 to -0.12)	13.46 (4.3 to 29.15)	5.62 (3.09 to 9.01)	-0.58 (-0.8 to -0.06)
Zambia	1.58 (0.99 to 2.32)	1.68 (1.07 to 2.47)	0.07 (-0.05 to 0.2)	0.04 (0.02 to 0.08)	0.04 (0.02 to 0.08)	0.06 (-0.4 to 0.97)	1.29 (0.75 to 2.76)	1.41 (0.79 to 2.63)	0.09 (-0.36 to 0.95)

DALY, Disability-Adjusted Life Year; UI, uncertainty interval.

Table S3. Age-standardized prevalence, death, DALYs rates of the other cardiomyopathy, and their percentage changes with 95% UIs from 1990 to 2019 at national level, both sexs.

Countries	Prevalence			Deaths			DALYs		
	1990 age-standardized rate per 100,000 people	2019 age-standardized rate per 100,000 people	Percentage change in age-standardized rates, 1990–2019	1990 age-standardized rate per 100,000 people	2019 age-standardized rate per 100,000 people	Percentage change in age-standardized rates, 1990–2019	1990 age-standardized rate per 100,000 people	2019 age-standardized rate per 100,000 people	Percentage change in age-standardized rates, 1990–2019
Afghanistan	7.47 (5.65 to 9.64)	7.85 (6.04 to 9.92)	0.05 (-0.05 to 0.17)	2.39 (1.16 to 4.71)	2.12 (1.09 to 3.8)	-0.11 (-0.34 to 0.27)	60 (27.86 to 141.65)	49.73 (25.81 to 98.85)	-0.17 (-0.4 to 0.23)
Albania	65.13 (46.97 to 89)	54.64 (39.57 to 75.85)	-0.16 (-0.25 to -0.04)	10.71 (6.81 to 12.78)	5.8 (3.99 to 9.28)	-0.46 (-0.64 to 0)	178.98 (127.34 to 208.11)	109.95 (76.6 to 179.05)	-0.39 (-0.58 to 0.02)
Algeria	19.54 (14.35 to 25.96)	20.21 (15.01 to 26.64)	0.03 (-0.06 to 0.14)	3.03 (2.15 to 4.76)	2.13 (1.54 to 3.2)	-0.3 (-0.52 to 0)	79.55 (54.07 to 144.1)	50.58 (36.06 to 77.96)	-0.36 (-0.58 to -0.03)
American Samoa	85.6 (63.67 to 113.1)	86.33 (63.7 to 113.72)	0.01 (-0.09 to 0.14)	7.62 (6.13 to 10.62)	7.04 (5.45 to 9.28)	-0.08 (-0.29 to 0.21)	217.19 (175.86 to 297.53)	207.72 (158.07 to 276.75)	-0.04 (-0.28 to 0.27)
Andorra	80.41 (60.92 to 103.2)	58.47 (44.4 to 75.59)	-0.27 (-0.35 to -0.19)	4.8 (2.78 to 6.91)	4.47 (2.95 to 5.93)	-0.07 (-0.36 to 0.37)	78.86 (53.85 to 110.06)	71 (51.34 to 92.51)	-0.1 (-0.38 to 0.3)
Angola	199.11 (129.1 to 286.16)	219.27 (142.57 to 309.53)	0.1 (-0.02 to 0.23)	9.57 (4.86 to 14.56)	8.47 (4.96 to 12.19)	-0.11 (-0.39 to 0.3)	274.47 (139.35 to 396.87)	233.26 (146.1 to 319.43)	-0.15 (-0.43 to 0.31)
Antigua and Barbuda	29.49 (23.07 to 37.35)	30.88 (23.24 to 40.49)	0.05 (-0.13 to 0.23)	3.94 (3.45 to 4.86)	4.62 (3.26 to 5.6)	0.17 (-0.25 to 0.5)	101.24 (89.36 to 126.03)	107.02 (77.26 to 129.63)	0.06 (-0.31 to 0.35)
Argentina	61.27 (41.51 to 85.45)	78.84 (55.67 to 108.79)	0.29 (0.06 to 0.56)	13.76 (9.55 to 16.29)	10.83 (8.55 to 12.08)	-0.21 (-0.35 to 0)	292.93 (232.11 to 335.07)	212.2 (186.43 to 237.82)	-0.28 (-0.38 to -0.09)

Armenia	14.97 (11.38 to 19.29)	7.48 (5.65 to 9.7)	-0.5 (-0.56 to -0.43)	1.46 (0.56 to 2.88)	2.54 (1.32 to 3.41)	0.73 (-0.29 to 2.34)	29.63 (20.16 to 48.96)	47.52 (30.89 to 61.61)	0.6 (-0.3 to 1.69)
Australia	111.87 (85.56 to 146.7)	76.29 (57.99 to 98.66)	-0.32 (-0.39 to -0.24)	5.4 (3.27 to 6.11)	2.17 (1.84 to 2.82)	-0.6 (-0.67 to -0.27)	118.87 (86.78 to 131.37)	49.91 (42.96 to 67.27)	-0.58 (-0.65 to -0.34)
Austria	173.02 (129.87 to 220.58)	117.87 (89.62 to 149.26)	-0.32 (-0.4 to -0.22)	35.64 (7.61 to 42.68)	7.08 (4.85 to 8.29)	-0.8 (-0.84 to -0.31)	448.09 (150.38 to 521.19)	92.78 (80.25 to 116.3)	-0.79 (-0.83 to -0.38)
Azerbaijan	55.22 (41.69 to 71.35)	51.08 (38.26 to 66.03)	-0.08 (-0.17 to 0.02)	14.07 (11.03 to 19.51)	15.59 (11.92 to 22.12)	0.11 (-0.13 to 0.42)	399.33 (298.18 to 596.85)	393.65 (286.13 to 638.08)	-0.01 (-0.21 to 0.23)
Bahamas	34.22 (26.15 to 44.55)	33.49 (25.62 to 43.52)	-0.02 (-0.12 to 0.09)	7.36 (6.33 to 8.68)	7.37 (5.92 to 9.01)	0 (-0.23 to 0.27)	208.99 (179.73 to 247.38)	203.05 (158.9 to 255.01)	-0.03 (-0.25 to 0.25)
Bahrain	74.76 (56.17 to 98.11)	77.09 (58.45 to 101.79)	0.03 (-0.08 to 0.15)	5.24 (4.06 to 7.43)	4.14 (3 to 5.51)	-0.21 (-0.45 to 0.07)	138.46 (112.56 to 190.31)	92.94 (69.27 to 125.51)	-0.33 (-0.53 to -0.09)
Bangladesh	1.29 (0.91 to 1.79)	1.37 (0.97 to 1.87)	0.06 (-0.05 to 0.19)	0.28 (0.16 to 0.52)	0.33 (0.19 to 0.51)	0.17 (-0.32 to 0.98)	6.36 (3.58 to 11.06)	7.27 (4.11 to 11.36)	0.14 (-0.39 to 0.97)
Barbados	51.97 (40.08 to 66.59)	54.77 (41.7 to 71.34)	0.05 (-0.05 to 0.18)	5 (4.35 to 5.92)	5.07 (4.09 to 6.18)	0.01 (-0.21 to 0.26)	129.84 (114.07 to 150.91)	119.82 (95.83 to 147.99)	-0.08 (-0.3 to 0.16)
Belarus	11.71 (8.97 to 15.1)	14.76 (11.45 to 18.95)	0.26 (0.13 to 0.4)	3.38 (1.95 to 4.62)	1.88 (1.07 to 3.4)	-0.44 (-0.67 to -0.04)	119.47 (62.04 to 163.98)	71.2 (38.8 to 116.32)	-0.4 (-0.63 to -0.02)
Belgium	82.87 (61.54 to 107.08)	69.63 (55.66 to 84.94)	-0.16 (-0.31 to 0.03)	6.61 (3.66 to 7.75)	3.47 (2.67 to 4.06)	-0.47 (-0.57 to -0.21)	107.16 (72.74 to 120.44)	51.94 (45.33 to 63.9)	-0.52 (-0.59 to -0.28)
Belize	45.12 (34.55 to 60.05)	48.97 (37.04 to 63.72)	0.09 (-0.02 to 0.21)	5.08 (3.72 to 6.58)	5.57 (4.38 to 6.5)	0.1 (-0.2 to 0.59)	146 (111.19 to 180.89)	149.9 (118.78 to 175.49)	0.03 (-0.23 to 0.45)
Benin	206.76 (134.09 to 295.47)	214.24 (140 to 304.97)	0.04 (-0.07 to 0.15)	8.12 (4.32 to 11.73)	5.38 (3.83 to 7.12)	-0.34 (-0.54 to 0.05)	213.43 (120.69 to 295.89)	149.73 (109.04 to 201.38)	-0.3 (-0.51 to 0.1)
Bermuda	62 (47.62 to 80.16)	58.47 (44.88 to 76.43)	-0.06 (-0.14 to 0.05)	5.79 (4.59 to 7.76)	4.79 (3.4 to 5.95)	-0.17 (-0.48 to 0.17)	137.95 (110.08 to 186.54)	105.67 (78.53 to 130.74)	-0.23 (-0.53 to 0.11)

Bhutan	0.97 (0.66 to 1.34)	1.11 (0.76 to 1.56)	0.15 (0.02 to 0.29)	0.24 (0.1 to 0.49)	0.34 (0.21 to 0.55)	0.41 (-0.11 to 1.6)	5.15 (2.3 to 9.87)	6.83 (4.17 to 11.44)	0.33 (-0.22 to 1.49)
Bolivia (Plurinational State of)	5.36 (3.17 to 8.84)	10 (6.71 to 14.54)	0.87 (0.5 to 1.47)	2.46 (1.56 to 3.61)	2.33 (1.55 to 3.11)	-0.05 (-0.33 to 0.37)	77.42 (43.7 to 120.33)	60.33 (39.49 to 81.01)	-0.22 (-0.49 to 0.2)
Bosnia and Herzegovina	113.36 (81.97 to 155.61)	88.38 (62.8 to 120.75)	-0.22 (-0.31 to -0.13)	11.62 (6.64 to 17.13)	13.66 (7.19 to 19.3)	0.18 (-0.2 to 0.66)	198.66 (133.56 to 274.94)	222.32 (122.15 to 316.6)	0.12 (-0.21 to 0.53)
Botswana	236.44 (151.59 to 335.73)	239.35 (155.69 to 338.92)	0.01 (-0.09 to 0.11)	15.69 (12.06 to 21.18)	11.78 (8.43 to 17.46)	-0.25 (-0.48 to 0.03)	348.19 (270.17 to 471.86)	273.81 (196.98 to 400.76)	-0.21 (-0.45 to 0.06)
Brazil	79.81 (57.19 to 108.93)	76.83 (55.33 to 103.49)	-0.04 (-0.08 to 0.01)	14.06 (10.82 to 15.21)	8.63 (7.58 to 10.1)	-0.39 (-0.45 to -0.21)	330.84 (260.38 to 355.15)	209.49 (187.69 to 235.89)	-0.37 (-0.43 to -0.23)
Brunei Darussalam	58.74 (43.68 to 77.36)	47.34 (34.42 to 63.36)	-0.19 (-0.28 to -0.11)	7.56 (5.8 to 11.51)	8.15 (6.37 to 9.78)	0.08 (-0.27 to 0.46)	202.4 (159.73 to 307.02)	196.53 (160.33 to 238.17)	-0.03 (-0.34 to 0.28)
Bulgaria	17.88 (12.67 to 24.72)	15.21 (10.69 to 21.1)	-0.15 (-0.26 to -0.01)	2.13 (1.05 to 7.29)	5.09 (3.94 to 6.69)	1.39 (-0.18 to 3.85)	50.66 (31.73 to 136.07)	111.35 (85.14 to 141.57)	1.2 (-0.21 to 2.92)
Burkina Faso	199.53 (131.85 to 282.06)	196.61 (126.89 to 278.8)	-0.01 (-0.12 to 0.1)	8.7 (4.48 to 13.94)	7.17 (5.1 to 9.77)	-0.18 (-0.42 to 0.3)	218.9 (115.43 to 337.47)	185.25 (133.66 to 249.49)	-0.15 (-0.41 to 0.32)
Burundi	262.98 (175.01 to 367.41)	269.52 (174.69 to 384.06)	0.02 (-0.08 to 0.14)	5.18 (3.26 to 7.66)	3.71 (2.52 to 5.05)	-0.28 (-0.53 to 0.13)	196 (119.81 to 286.23)	138.64 (98.44 to 188.36)	-0.29 (-0.54 to 0.11)
Cabo Verde	243.96 (156.46 to 345.8)	263.09 (173.74 to 371.11)	0.08 (-0.03 to 0.19)	2.22 (1.67 to 2.84)	2.99 (2.29 to 3.88)	0.35 (0.01 to 0.77)	79.08 (61.56 to 100.04)	89.51 (71.42 to 113.43)	0.13 (-0.12 to 0.42)
Cambodia	25.02 (18.31 to 33.5)	26.66 (19.47 to 35.22)	0.07 (-0.05 to 0.21)	3.07 (2.04 to 4.56)	3.24 (2.45 to 4.36)	0.06 (-0.24 to 0.49)	75.54 (47.87 to 138.25)	70.38 (53.31 to 103.71)	-0.07 (-0.4 to 0.37)
Cameroon	170.37 (119.39 to 236.72)	201.24 (127.27 to 291.84)	0.18 (-0.05 to 0.43)	8.07 (4.19 to 11.31)	5.66 (3.95 to 7.83)	-0.3 (-0.52 to 0.16)	198.48 (106.3 to 273.91)	153.35 (108.12 to 212.77)	-0.23 (-0.48 to 0.29)
Canada	203.86 (155.42 to 263.5)	102.35 (83.49 to 125.31)	-0.5 (-0.57 to -0.43)	2.63 (2.29 to 3.12)	1.67 (1.37 to 2.37)	-0.36 (-0.47 to -0.14)	83.61 (71.3 to 94.44)	47.27 (40.9 to 62.06)	-0.43 (-0.51 to -0.26)

Central African Republic	189.89 (122.83 to 273.03)	174.36 (113.58 to 248.42)	-0.08 (-0.19 to 0.02)	11.51 (5.62 to 19.05)	9.83 (5.03 to 15.75)	-0.15 (-0.37 to 0.17)	316.23 (152.68 to 495.06)	268.91 (137.11 to 404)	-0.15 (-0.37 to 0.16)
Chad	189.83 (123.42 to 272.13)	201.09 (127.74 to 286.08)	0.06 (-0.05 to 0.18)	7.76 (3.97 to 12.46)	5.83 (4.15 to 7.83)	-0.25 (-0.49 to 0.24)	198.55 (106.91 to 296.4)	157 (112.69 to 213.44)	-0.21 (-0.46 to 0.27)
Chile	87.41 (61.7 to 121.7)	70.77 (51.04 to 95.18)	-0.19 (-0.3 to -0.08)	4.15 (3.61 to 6.04)	3.4 (2.94 to 4.47)	-0.18 (-0.3 to -0.01)	105.65 (93.93 to 138.53)	85.07 (75.88 to 102.4)	-0.19 (-0.34 to -0.04)
China	9.06 (6.89 to 11.67)	9.7 (7.33 to 12.42)	0.07 (0.03 to 0.11)	0.75 (0.47 to 1.49)	0.7 (0.5 to 0.93)	-0.06 (-0.56 to 0.65)	27 (17.24 to 52.45)	19.4 (14.79 to 24.41)	-0.28 (-0.64 to 0.24)
Colombia	28.07 (20.94 to 37.93)	24.91 (18.69 to 33.3)	-0.11 (-0.21 to -0.01)	2.6 (2.29 to 2.94)	1.89 (1.42 to 2.48)	-0.27 (-0.44 to -0.04)	69.74 (62.65 to 79.71)	48.52 (36.53 to 66.1)	-0.3 (-0.48 to -0.05)
Comoros	277.16 (184.77 to 393.35)	286.66 (191.11 to 400.68)	0.03 (-0.08 to 0.16)	4.38 (2.48 to 6.25)	3.35 (2.12 to 4.65)	-0.23 (-0.48 to 0.35)	169.16 (95.23 to 243.89)	126.78 (81.89 to 170.32)	-0.25 (-0.49 to 0.37)
Congo	234.67 (153.59 to 333.51)	235.52 (151.86 to 333.47)	0 (-0.1 to 0.12)	10.89 (5.84 to 15.35)	8.26 (4.78 to 11.85)	-0.24 (-0.48 to 0.09)	295.2 (154.66 to 401.72)	221.61 (135.77 to 309.67)	-0.25 (-0.49 to 0.12)
Cook Islands	8.76 (6.5 to 11.57)	8.72 (6.48 to 11.59)	0 (-0.11 to 0.1)	1.21 (0.87 to 1.61)	0.74 (0.56 to 0.97)	-0.39 (-0.54 to -0.16)	36.11 (25.8 to 49.8)	20.38 (14.78 to 26.74)	-0.44 (-0.6 to -0.18)
Costa Rica	48.14 (35.82 to 63.57)	46.88 (35.59 to 61.31)	-0.03 (-0.13 to 0.1)	4.94 (4.21 to 5.69)	3.71 (2.74 to 5.27)	-0.25 (-0.44 to 0)	124.31 (110.36 to 140.15)	92.78 (70.07 to 124.14)	-0.25 (-0.43 to -0.03)
Croatia	30.97 (22.01 to 42.45)	19.6 (14.37 to 26.18)	-0.37 (-0.51 to -0.18)	5.62 (4.39 to 8.07)	4.2 (3.01 to 5.86)	-0.25 (-0.43 to -0.04)	89.94 (74.04 to 139.74)	53.76 (39.2 to 83.25)	-0.4 (-0.54 to -0.23)
Cuba	22.61 (17.33 to 29.63)	26.74 (20.45 to 35.25)	0.18 (0.05 to 0.35)	1.81 (1.43 to 3.17)	2.25 (1.73 to 2.8)	0.25 (-0.32 to 0.71)	56.29 (45.53 to 85.09)	54.45 (43.29 to 67.69)	-0.03 (-0.37 to 0.3)
Cyprus	57.76 (43.85 to 74.96)	48.22 (37.96 to 60.28)	-0.17 (-0.28 to -0.01)	6.36 (3.95 to 9.73)	4.13 (3.24 to 5.84)	-0.35 (-0.52 to -0.05)	126.87 (83.67 to 161.44)	76.17 (59.34 to 93.22)	-0.4 (-0.54 to -0.16)
Czechia	42.41 (31.38 to 56.75)	46.67 (33.93 to 64.93)	0.1 (-0.08 to 0.36)	3.65 (3.16 to 6.54)	2.69 (2.12 to 3.76)	-0.26 (-0.48 to -0.06)	110.55 (92.07 to 133.28)	68.12 (50.87 to 84.94)	-0.38 (-0.57 to -0.22)

Cote d'Ivoire	231.05 (151.72 to 328.54)	232.18 (149.45 to 331.58)	0 (-0.09 to 0.11)	7.65 (3.95 to 10.7)	4.98 (3.5 to 6.67)	-0.35 (-0.55 to 0.02)	195.73 (104.97 to 265.31)	137.38 (97.41 to 179.47)	-0.3 (-0.5 to 0.1)
Democratic People's Republic of Korea	11.6 (8.8 to 15.27)	11.18 (8.41 to 14.56)	-0.04 (-0.13 to 0.06)	1.54 (1.01 to 2.44)	1.35 (0.92 to 1.92)	-0.12 (-0.37 to 0.26)	47.88 (30.42 to 81.61)	37.02 (25.77 to 51.86)	-0.23 (-0.48 to 0.14)
Democratic Republic of the Congo	213.09 (138 to 307.97)	202.91 (128.95 to 290.97)	-0.05 (-0.14 to 0.06)	9.47 (5.21 to 14.73)	8.54 (4.87 to 14.88)	-0.1 (-0.35 to 0.3)	262.24 (145.92 to 383.34)	228.57 (138.35 to 375.1)	-0.13 (-0.37 to 0.25)
Denmark	24.62 (19.4 to 30.78)	17.21 (13.6 to 21.48)	-0.3 (-0.36 to -0.24)	1.63 (1.27 to 3.37)	1.45 (1.13 to 2.74)	-0.11 (-0.23 to 0.05)	43.83 (37.72 to 63.3)	32.75 (28.16 to 44.57)	-0.25 (-0.36 to -0.11)
Djibouti	273.75 (177.8 to 387.12)	305.28 (200.4 to 429.37)	0.12 (0.01 to 0.24)	4.67 (2.76 to 6.62)	3.99 (2.18 to 6.02)	-0.15 (-0.41 to 0.27)	176.18 (108.98 to 252.04)	150.63 (90.46 to 232.68)	-0.15 (-0.4 to 0.25)
Dominica	80.82 (61.99 to 104.76)	82.46 (62.36 to 107.13)	0.02 (-0.09 to 0.14)	18.9 (15.34 to 25.85)	21.04 (15.61 to 26.61)	0.11 (-0.21 to 0.52)	472.59 (383.04 to 640.76)	514.14 (377.01 to 662.23)	0.09 (-0.23 to 0.51)
Dominican Republic	14.39 (11.06 to 18.83)	16.36 (12.5 to 21.73)	0.14 (0.03 to 0.26)	1.74 (1.39 to 2.43)	2.19 (1.38 to 2.9)	0.26 (-0.22 to 0.83)	54.87 (44.35 to 75.32)	58.94 (37.63 to 79.47)	0.07 (-0.33 to 0.55)
Ecuador	12.57 (8.84 to 17.4)	14.94 (11.03 to 19.93)	0.19 (0.05 to 0.37)	1.48 (1.16 to 2.24)	1.7 (1.27 to 2.2)	0.15 (-0.23 to 0.64)	41.02 (32.48 to 61.75)	44.33 (33.44 to 57.54)	0.08 (-0.26 to 0.55)
Egypt	22.13 (15.21 to 32.1)	26.27 (17.79 to 37.55)	0.19 (-0.02 to 0.42)	3.56 (2.18 to 5.9)	2.53 (1.71 to 3.94)	-0.29 (-0.51 to 0.08)	159.06 (85.35 to 303.6)	88.64 (58 to 139.38)	-0.44 (-0.69 to 0.06)
El Salvador	6.93 (4.89 to 9.54)	8.08 (5.81 to 10.86)	0.17 (0.04 to 0.31)	0.83 (0.61 to 1)	0.68 (0.48 to 0.92)	-0.18 (-0.42 to 0.16)	25.31 (18.08 to 31.38)	18.65 (12.82 to 25.22)	-0.26 (-0.49 to 0.06)
Equatorial Guinea	197.44 (126.11 to 278.97)	229.08 (150.03 to 325.18)	0.16 (0.02 to 0.32)	10.2 (4.81 to 16.93)	6.97 (3.34 to 11.16)	-0.32 (-0.65 to 0.34)	283.83 (130.35 to 445.99)	182.37 (92.56 to 292.26)	-0.36 (-0.66 to 0.27)
Eritrea	200.06 (130.66 to 287.43)	222.32 (146.03 to 320.9)	0.11 (-0.01 to 0.24)	4.41 (2.63 to 6.45)	3.84 (2.23 to 5.38)	-0.13 (-0.4 to 0.26)	162.31 (94.68 to 237.35)	137.15 (86.6 to 193.47)	-0.16 (-0.41 to 0.23)
Estonia	39.93 (29.4 to 53.86)	45.67 (33.77 to 61.46)	0.14 (0.03 to 0.27)	8.42 (4.73 to 10.46)	4.9 (3.31 to 6.36)	-0.42 (-0.58 to -0.08)	300.24 (168.69 to 376.24)	156.42 (108.09 to 202.91)	-0.48 (-0.63 to -0.16)

Eswatini	222.71 (142.81 to 319.13)	214.23 (135.84 to 309.05)	-0.04 (-0.14 to 0.07)	14.82 (11.16 to 19.99)	11.87 (8.18 to 18.19)	-0.2 (-0.44 to 0.13)	331.45 (253.31 to 435.99)	270.21 (189.97 to 399.87)	-0.18 (-0.41 to 0.15)
Ethiopia	261.67 (191.71 to 338.2)	304.53 (225.19 to 391.35)	0.16 (0.1 to 0.23)	4.27 (2.53 to 6.56)	3.08 (2.06 to 3.98)	-0.28 (-0.57 to 0.26)	166.93 (100.6 to 251.35)	115.86 (83.78 to 146.55)	-0.31 (-0.59 to 0.19)
Fiji	23.7 (17.47 to 31.35)	23.54 (17.58 to 31.32)	-0.01 (-0.11 to 0.12)	3.37 (2.66 to 4.57)	3.32 (2.38 to 4.28)	-0.01 (-0.3 to 0.37)	100.3 (78.74 to 134.68)	104.65 (74.8 to 137.35)	0.04 (-0.27 to 0.5)
Finland	42.5 (34.03 to 53.2)	41.24 (32.56 to 51.57)	-0.03 (-0.15 to 0.1)	2.52 (1.64 to 6.5)	2.92 (2.39 to 4.63)	0.16 (-0.33 to 0.74)	63.06 (46.31 to 112.61)	64.38 (54.57 to 73.67)	0.02 (-0.39 to 0.45)
France	144.93 (113.03 to 182.81)	77.02 (59.99 to 96.79)	-0.47 (-0.52 to -0.41)	6.18 (4.5 to 6.95)	3.36 (2.73 to 4.09)	-0.46 (-0.54 to -0.27)	115.08 (94.5 to 125.76)	58.47 (50.47 to 76.83)	-0.49 (-0.56 to -0.3)
Gabon	234.4 (151.46 to 331.98)	233.59 (153.46 to 334.82)	0 (-0.1 to 0.1)	9.88 (4.94 to 14.5)	7.83 (4.18 to 10.96)	-0.21 (-0.49 to 0.12)	272.03 (139.62 to 386.3)	211.25 (117.67 to 295.33)	-0.22 (-0.48 to 0.11)
Gambia	243.95 (158.47 to 347.91)	256.22 (167.83 to 357.81)	0.05 (-0.05 to 0.16)	7.13 (3.89 to 10.82)	5.53 (3.96 to 7.46)	-0.23 (-0.48 to 0.25)	183.19 (105.47 to 266.22)	149.92 (111.12 to 198.91)	-0.18 (-0.45 to 0.3)
Georgia	16.49 (12.38 to 21.6)	15.17 (10.6 to 20.85)	-0.08 (-0.21 to 0.03)	3.18 (2.37 to 3.91)	3.74 (2.39 to 5.21)	0.18 (-0.14 to 0.53)	82.58 (65.03 to 103.46)	103.69 (71.76 to 146.82)	0.26 (-0.15 to 0.7)
Germany	55.62 (43.32 to 70.47)	44.8 (35.44 to 56.58)	-0.19 (-0.28 to -0.08)	5.43 (4.17 to 6.62)	3.38 (2.86 to 4.12)	-0.38 (-0.5 to -0.18)	94.8 (76.75 to 115.37)	55.3 (49.25 to 66.06)	-0.42 (-0.53 to -0.24)
Ghana	174.58 (121.39 to 247.02)	195.91 (125.16 to 285.23)	0.12 (-0.11 to 0.37)	7.87 (6.05 to 11.19)	9.48 (6.2 to 12.27)	0.2 (-0.25 to 0.76)	190.51 (146.35 to 261.27)	227.42 (156.63 to 298.34)	0.19 (-0.23 to 0.75)
Greece	56.65 (43.68 to 71.89)	42.71 (32.5 to 54.74)	-0.25 (-0.31 to -0.18)	5.95 (3.34 to 6.85)	3.42 (2.62 to 3.88)	-0.43 (-0.51 to -0.19)	96.41 (63.9 to 106.9)	69.56 (47.91 to 78.86)	-0.28 (-0.41 to -0.19)
Greenland	250.17 (194.96 to 315.81)	189.08 (147.36 to 241.96)	-0.24 (-0.32 to -0.17)	12.16 (9.11 to 14.89)	9.8 (7.01 to 12.5)	-0.19 (-0.4 to 0.08)	369.98 (285.37 to 448.35)	248.8 (180.01 to 324.82)	-0.33 (-0.5 to -0.08)
Grenada	46.59 (35.11 to 60.95)	47.31 (35.92 to 61.19)	0.02 (-0.08 to 0.11)	8.8 (7.69 to 10.19)	8.59 (6.73 to 9.9)	-0.02 (-0.28 to 0.17)	229.29 (199.91 to 265.18)	203.88 (159.6 to 236.03)	-0.11 (-0.32 to 0.07)

Guam	34.22 (25.38 to 45.44)	35.84 (26.7 to 47.67)	0.05 (-0.07 to 0.18)	3.8 (2.98 to 5.1)	2.36 (1.79 to 3.59)	-0.38 (-0.54 to -0.08)	104.85 (84.4 to 134.99)	81.86 (62.53 to 119.26)	-0.22 (-0.42 to 0.09)
Guatemala	8.35 (5.52 to 11.85)	10.78 (7.79 to 14.9)	0.29 (0.13 to 0.51)	1.06 (0.82 to 1.38)	1.15 (0.78 to 1.46)	0.09 (-0.39 to 0.59)	29.88 (21.77 to 42.82)	30.27 (21.07 to 39.32)	0.01 (-0.46 to 0.62)
Guinea	203.46 (146.65 to 274.08)	224.9 (150.45 to 313.63)	0.11 (-0.09 to 0.3)	8.22 (4.2 to 11.86)	6.02 (4.2 to 7.96)	-0.27 (-0.5 to 0.18)	214.5 (114.2 to 300.82)	167.39 (119.04 to 224.98)	-0.22 (-0.46 to 0.25)
Guinea-Bissau	198.11 (130.07 to 284.86)	212.88 (138.98 to 304.82)	0.07 (-0.04 to 0.2)	8.7 (3.95 to 14.25)	6.07 (4.14 to 8.5)	-0.3 (-0.54 to 0.23)	229.78 (105.35 to 373.45)	168.75 (116.19 to 237.41)	-0.27 (-0.51 to 0.33)
Guyana	26.1 (19.69 to 35.34)	22.89 (17.28 to 30.3)	-0.12 (-0.23 to -0.02)	7.43 (6.19 to 9.22)	7.89 (6.03 to 10.07)	0.06 (-0.25 to 0.48)	210.3 (173.29 to 253.99)	211.87 (157.06 to 276.13)	0.01 (-0.28 to 0.44)
Haiti	21.74 (16.13 to 29.04)	24.16 (18.36 to 32.05)	0.11 (-0.02 to 0.25)	8.97 (5.17 to 14.11)	7.86 (4.56 to 12)	-0.12 (-0.37 to 0.23)	258.77 (141.77 to 421.67)	229.99 (127.09 to 380.7)	-0.11 (-0.4 to 0.29)
Honduras	25.24 (17.46 to 35.85)	30.04 (21.69 to 41.66)	0.19 (0.04 to 0.4)	4.52 (3.1 to 6.21)	5.45 (3.57 to 7.47)	0.21 (-0.08 to 0.59)	110.81 (78.93 to 147.58)	118.57 (74.82 to 163.82)	0.07 (-0.2 to 0.46)
Hungary	35.18 (24.25 to 49.87)	32.43 (21.06 to 47.01)	-0.08 (-0.24 to 0.11)	14.61 (8.43 to 16.5)	3.72 (2.57 to 7.85)	-0.74 (-0.83 to -0.19)	247.5 (149.85 to 279.26)	62.75 (44.42 to 124.43)	-0.75 (-0.83 to -0.25)
Iceland	30.98 (23.62 to 39.94)	25.33 (20.05 to 31.98)	-0.18 (-0.3 to -0.03)	2.05 (1.5 to 2.39)	1.16 (0.94 to 1.41)	-0.44 (-0.53 to -0.24)	40.61 (30.72 to 46.49)	21.36 (18.15 to 25.87)	-0.47 (-0.56 to -0.31)
India	1.14 (0.79 to 1.57)	1.19 (0.83 to 1.63)	0.05 (0.02 to 0.07)	0.26 (0.16 to 0.44)	0.24 (0.18 to 0.33)	-0.09 (-0.36 to 0.3)	5.73 (3.49 to 9.19)	5.33 (3.9 to 7.17)	-0.07 (-0.35 to 0.34)
Indonesia	36.39 (27.76 to 47.29)	40.44 (30.9 to 52.65)	0.11 (0.06 to 0.16)	3.29 (2.59 to 4.5)	4.01 (2.76 to 5.28)	0.22 (-0.13 to 0.58)	76.25 (58.92 to 120.8)	79.4 (56.3 to 110.74)	0.04 (-0.29 to 0.37)
Iran (Islamic Republic of)	19.89 (15.08 to 25.78)	20.08 (15.25 to 25.86)	0.01 (-0.03 to 0.04)	2.14 (1.56 to 2.86)	1.54 (1.07 to 1.93)	-0.28 (-0.5 to -0.04)	58.35 (45.11 to 77.15)	37.95 (27.55 to 46.74)	-0.35 (-0.56 to -0.14)
Iraq	47.27 (36.76 to 61.26)	46.57 (35.94 to 59.53)	-0.01 (-0.11 to 0.09)	5.08 (3.66 to 7.6)	4.47 (3.26 to 6.68)	-0.12 (-0.4 to 0.24)	157.63 (116.7 to 219.9)	117.51 (88.74 to 163.93)	-0.25 (-0.49 to 0.06)

Ireland	69.6 (53.36 to 90.17)	50.57 (38.76 to 64.7)	-0.27 (-0.34 to -0.19)	11.87 (5.93 to 13.67)	3.33 (2.67 to 4.56)	-0.72 (-0.79 to -0.39)	178.5 (107.39 to 200.16)	53.74 (44.53 to 75.45)	-0.7 (-0.76 to -0.44)
Israel	40.33 (31.24 to 51.4)	31 (24.08 to 39.32)	-0.23 (-0.31 to -0.14)	2.9 (2.55 to 3.74)	1.43 (1.1 to 2.56)	-0.51 (-0.61 to -0.28)	73.83 (60.11 to 81.74)	31.9 (26.98 to 46.7)	-0.57 (-0.65 to -0.34)
Italy	105.46 (73.94 to 144.58)	132.54 (96.49 to 173.69)	0.26 (0.11 to 0.45)	25.52 (6.26 to 30.28)	3.87 (3.16 to 5.05)	-0.85 (-0.88 to -0.31)	322.34 (121.02 to 370.94)	67.72 (57.08 to 95.78)	-0.79 (-0.84 to -0.33)
Jamaica	28.8 (22.66 to 36.46)	33.14 (25.65 to 41.58)	0.15 (0.04 to 0.26)	3.59 (2.87 to 4.25)	3.25 (2.57 to 4.16)	-0.1 (-0.32 to 0.21)	104 (82.13 to 121.34)	92.99 (71.87 to 118.57)	-0.11 (-0.33 to 0.21)
Japan	80.25 (60.48 to 105.19)	72.47 (57.89 to 89.3)	-0.1 (-0.21 to 0.02)	4.74 (2.42 to 5.31)	2 (1.43 to 2.25)	-0.58 (-0.63 to -0.32)	102.78 (73.9 to 111.25)	49.66 (42.58 to 57.08)	-0.52 (-0.56 to -0.35)
Jordan	10.3 (7.88 to 13.02)	11.04 (8.42 to 13.85)	0.07 (-0.02 to 0.17)	1.11 (0.72 to 1.45)	0.56 (0.43 to 0.8)	-0.5 (-0.67 to -0.16)	29.04 (18.09 to 38.38)	14.06 (11.1 to 19.11)	-0.52 (-0.68 to -0.18)
Kazakhstan	68.45 (50.83 to 91.71)	64.67 (47.37 to 86.38)	-0.06 (-0.15 to 0.05)	3 (1.74 to 8.46)	24.02 (7.73 to 30.99)	7.01 (0.08 to 14.52)	104.54 (61.29 to 289.89)	750.78 (265.98 to 984.3)	6.18 (0.06 to 12.6)
Kenya	223.22 (147.82 to 316.89)	215.84 (143.54 to 305.28)	-0.03 (-0.06 to -0.01)	3.15 (2.17 to 4.17)	3.26 (2.12 to 4.51)	0.04 (-0.13 to 0.24)	114.49 (82.58 to 143.85)	109.58 (73.15 to 149.36)	-0.04 (-0.19 to 0.15)
Kiribati	12.04 (8.52 to 16.66)	12.6 (8.94 to 17.2)	0.05 (-0.07 to 0.17)	2.63 (1.23 to 3.53)	2.37 (0.99 to 3.41)	-0.1 (-0.33 to 0.2)	85.42 (37.2 to 122.66)	77.9 (29.4 to 120.44)	-0.09 (-0.37 to 0.28)
Kuwait	20.65 (15.43 to 26.79)	21.12 (15.95 to 27.62)	0.02 (-0.06 to 0.12)	1.54 (1.29 to 1.82)	1.02 (0.81 to 1.29)	-0.34 (-0.5 to -0.16)	46.53 (39.51 to 54.4)	26.2 (20.81 to 32.98)	-0.44 (-0.57 to -0.26)
Kyrgyzstan	2.74 (2.03 to 3.6)	2.96 (2.2 to 3.86)	0.08 (-0.05 to 0.25)	0.44 (0.34 to 0.69)	0.49 (0.38 to 0.61)	0.11 (-0.34 to 0.55)	14.23 (11.18 to 20.98)	16.24 (9.89 to 20.21)	0.14 (-0.43 to 0.65)
Lao People's Democratic Republic	22.65 (16.79 to 30.14)	24.99 (18.45 to 33.29)	0.1 (-0.01 to 0.25)	3.24 (2.04 to 5.29)	3.71 (2.75 to 4.85)	0.15 (-0.24 to 0.68)	71.52 (43.47 to 149.62)	80.23 (57.87 to 116.43)	0.12 (-0.31 to 0.7)
Latvia	53.2 (39.69 to 71.24)	86.6 (62.95 to 117.33)	0.63 (0.36 to 0.9)	13.15 (10.21 to 15.17)	7.68 (5.56 to 9.66)	-0.42 (-0.57 to -0.22)	500.19 (357.7 to 581.57)	228.9 (171.44 to 293.87)	-0.54 (-0.66 to -0.33)

Lebanon	28.58 (21.59 to 37.69)	30.43 (23.14 to 38.81)	0.06 (-0.03 to 0.17)	2.87 (2.07 to 4.26)	1.85 (1.32 to 2.75)	-0.35 (-0.53 to -0.11)	77.75 (55.28 to 125.31)	48.62 (34.72 to 77.68)	-0.37 (-0.55 to -0.13)
Lesotho	193.61 (122.83 to 276.82)	194.08 (125.08 to 281.28)	0 (-0.1 to 0.12)	14.96 (11.05 to 21.27)	14.98 (10.71 to 22.67)	0 (-0.31 to 0.41)	325.69 (246.16 to 448.1)	327.38 (234.74 to 489.36)	0.01 (-0.3 to 0.41)
Liberia	226.04 (146.77 to 322.19)	251.65 (166.14 to 356.45)	0.11 (0.01 to 0.23)	7.72 (4.09 to 11.26)	5.1 (3.46 to 7.43)	-0.34 (-0.56 to 0.14)	201.04 (112.91 to 284.65)	136.96 (96.99 to 196.11)	-0.32 (-0.54 to 0.19)
Libya	21.52 (16.53 to 28.48)	24.65 (18.8 to 32.93)	0.15 (0.03 to 0.26)	2.93 (2 to 4.23)	1.73 (1.25 to 2.48)	-0.41 (-0.62 to -0.05)	153.09 (92.97 to 252.28)	70.68 (48.65 to 101.62)	-0.54 (-0.74 to -0.13)
Lithuania	22.09 (15.77 to 30.38)	24.25 (17.17 to 33.36)	0.1 (-0.03 to 0.22)	4.44 (3.7 to 5.23)	4.7 (2.78 to 6.13)	0.06 (-0.32 to 0.39)	156.9 (132.08 to 184.88)	153.79 (94.48 to 199.82)	-0.02 (-0.36 to 0.29)
Luxembourg	83.66 (63.11 to 110.37)	80.35 (62.51 to 99.89)	-0.04 (-0.2 to 0.17)	6.3 (4.44 to 7.18)	3.09 (2.46 to 3.96)	-0.51 (-0.62 to -0.24)	112.3 (84.96 to 124.24)	51.44 (42.38 to 68.03)	-0.54 (-0.64 to -0.32)
Madagascar	255.11 (166.26 to 359.55)	271.7 (178.35 to 380.83)	0.07 (-0.04 to 0.19)	6.7 (4.48 to 8.47)	5.63 (3.68 to 7.9)	-0.16 (-0.4 to 0.17)	267.38 (178.36 to 339.65)	213.15 (142.79 to 316.08)	-0.2 (-0.43 to 0.12)
Malawi	269.8 (174.73 to 387.62)	284.18 (187.79 to 400.84)	0.05 (-0.07 to 0.19)	4.1 (2.74 to 5.25)	3.72 (2.49 to 4.93)	-0.09 (-0.35 to 0.28)	164.27 (109.7 to 219.75)	138.37 (95.3 to 183.26)	-0.16 (-0.41 to 0.22)
Malaysia	70.77 (53.49 to 93.41)	81.04 (61.35 to 107.46)	0.15 (0 to 0.3)	3.29 (2.65 to 4.46)	2.9 (2.16 to 3.98)	-0.12 (-0.31 to 0.14)	96.64 (78.56 to 123.99)	84.31 (61.97 to 108.16)	-0.13 (-0.32 to 0.12)
Maldives	23.62 (17.69 to 31.19)	24.85 (18.55 to 33.12)	0.05 (-0.06 to 0.17)	1.44 (0.83 to 2.65)	1.34 (0.96 to 1.71)	-0.07 (-0.53 to 0.75)	44.54 (23.86 to 78.29)	32.93 (23.92 to 42.28)	-0.26 (-0.64 to 0.45)
Mali	190.7 (119.69 to 270.57)	202.56 (129.26 to 289.55)	0.06 (-0.05 to 0.18)	8.42 (4.36 to 13.44)	6.2 (4.49 to 8.34)	-0.26 (-0.52 to 0.26)	212.25 (111.05 to 316.37)	158.93 (117.33 to 215.25)	-0.25 (-0.5 to 0.27)
Malta	76.14 (57.77 to 98.13)	66.64 (50.84 to 84.4)	-0.12 (-0.24 to 0.02)	10.31 (6.55 to 11.94)	2.9 (2.27 to 4.52)	-0.72 (-0.78 to -0.39)	168.12 (120.98 to 187.66)	57 (46.57 to 80.51)	-0.66 (-0.73 to -0.4)
Marshall Islands	19.51 (14.36 to 26.61)	20.46 (14.9 to 27.97)	0.05 (-0.07 to 0.18)	4.24 (2.69 to 6.35)	4.03 (2.36 to 5.96)	-0.05 (-0.3 to 0.26)	113.96 (70.9 to 173.79)	113.77 (66.16 to 174.42)	0 (-0.28 to 0.35)

Mauritania	238.69 (156.55 to 336.6)	254.93 (166.7 to 358.15)	0.07 (-0.05 to 0.2)	7.97 (4.21 to 10.64)	4.71 (3.17 to 6.38)	-0.41 (-0.62 to 0.08)	206.71 (115.34 to 270.79)	125.71 (84.68 to 172.58)	-0.39 (-0.61 to 0.07)
Mauritius	35.48 (26.94 to 45.91)	33.26 (25.45 to 42.9)	-0.06 (-0.16 to 0.04)	1.55 (1.23 to 2.88)	3.07 (1.96 to 3.89)	0.98 (-0.24 to 1.99)	46.09 (36.7 to 85.17)	90.17 (56.74 to 115.96)	0.96 (-0.23 to 1.96)
Mexico	14.02 (10.34 to 18.37)	13.18 (9.89 to 17.1)	-0.06 (-0.09 to -0.03)	1.01 (0.87 to 1.59)	1.12 (0.93 to 1.34)	0.1 (-0.25 to 0.4)	31.7 (28.8 to 42.95)	37.27 (28.21 to 44.11)	0.18 (-0.27 to 0.46)
Micronesia (Federated States of)	19.5 (14.08 to 26.47)	19.34 (14.22 to 25.98)	-0.01 (-0.11 to 0.1)	4.1 (2.48 to 6.02)	4 (2.1 to 5.93)	-0.02 (-0.32 to 0.35)	113.51 (66.63 to 176.47)	109.87 (51.89 to 175.1)	-0.03 (-0.37 to 0.34)
Monaco	152.68 (116.82 to 197.01)	107.71 (81.93 to 139.78)	-0.29 (-0.37 to -0.22)	11.28 (7.32 to 14.45)	8.53 (6.22 to 10.6)	-0.24 (-0.45 to 0.03)	217.36 (148.97 to 273.68)	151.5 (112.33 to 192.17)	-0.3 (-0.48 to -0.06)
Mongolia	26.8 (19.67 to 35.77)	28 (20.24 to 37.16)	0.04 (-0.07 to 0.17)	8.64 (6.42 to 11.53)	7.49 (5.47 to 10.03)	-0.13 (-0.39 to 0.28)	229.67 (160.24 to 342.84)	193.3 (141.17 to 291.6)	-0.16 (-0.41 to 0.27)
Montenegro	111.03 (80.73 to 147.94)	92.7 (66.75 to 125.84)	-0.17 (-0.26 to -0.06)	12.64 (6.99 to 25.47)	20.02 (10.5 to 30)	0.58 (0 to 1.49)	201.36 (124.28 to 387.74)	274.33 (162.59 to 418.57)	0.36 (-0.08 to 0.99)
Morocco	17.19 (12.93 to 22.54)	18.04 (13.54 to 23.72)	0.05 (-0.05 to 0.16)	2.46 (1.54 to 3.94)	2.29 (1.63 to 3.45)	-0.07 (-0.33 to 0.28)	68.14 (41.23 to 123.99)	54.02 (37.01 to 87.84)	-0.21 (-0.46 to 0.16)
Mozambique	255.33 (167.15 to 361.89)	300.06 (202.2 to 418.79)	0.18 (0.05 to 0.34)	3.48 (2.66 to 4.54)	3.88 (2.84 to 5.21)	0.11 (-0.17 to 0.51)	135.45 (102.85 to 175.41)	145.07 (109.64 to 186.89)	0.07 (-0.21 to 0.45)
Myanmar	28.4 (21.03 to 37.56)	29.25 (21.32 to 38.73)	0.03 (-0.08 to 0.16)	3.5 (2.21 to 5.74)	3.42 (2.57 to 4.48)	-0.03 (-0.33 to 0.45)	89.43 (52.71 to 185.69)	80.4 (59.02 to 122.2)	-0.1 (-0.45 to 0.45)
Namibia	228.81 (145.44 to 321.98)	222.16 (141.14 to 314.22)	-0.03 (-0.13 to 0.08)	15.02 (11.29 to 19.7)	12.19 (9.19 to 17.29)	-0.19 (-0.42 to 0.11)	328.96 (242.96 to 423.91)	262.99 (196.44 to 375.6)	-0.2 (-0.42 to 0.14)
Nauru	21.93 (16.15 to 29.9)	22.03 (16.02 to 30.12)	0 (-0.1 to 0.12)	4.04 (2.43 to 5.79)	4 (2.37 to 5.95)	-0.01 (-0.23 to 0.31)	120.5 (71.23 to 181.77)	121.69 (72.92 to 182.38)	0.01 (-0.22 to 0.34)
Nepal	0.81 (0.55 to 1.14)	0.81 (0.55 to 1.12)	0 (-0.11 to 0.12)	0.23 (0.11 to 0.42)	0.3 (0.18 to 0.47)	0.3 (-0.13 to 1.12)	4.87 (2.44 to 8.71)	5.92 (3.48 to 9.54)	0.22 (-0.18 to 0.89)

Netherlands	58.03 (46.26 to 71.72)	42.34 (33.02 to 53.75)	-0.27 (-0.38 to -0.16)	7.16 (4.2 to 8.47)	2.88 (2.28 to 4.34)	-0.6 (-0.69 to -0.14)	111.04 (77.37 to 126.27)	45.54 (37.03 to 70.86)	-0.59 (-0.68 to -0.23)
New Zealand	61.47 (46.57 to 81.3)	46.9 (36.63 to 59.32)	-0.24 (-0.35 to -0.12)	4.22 (2.91 to 4.77)	2.25 (1.97 to 2.65)	-0.47 (-0.55 to -0.24)	121.42 (75.15 to 138.38)	62 (49.68 to 68.84)	-0.49 (-0.56 to -0.3)
Nicaragua	14.89 (10.59 to 20.49)	18.32 (13.57 to 24.4)	0.23 (0.11 to 0.4)	1.89 (1.49 to 2.29)	2.25 (1.63 to 2.8)	0.19 (-0.06 to 0.5)	53.45 (40.9 to 67.24)	51.27 (37.54 to 63.95)	-0.04 (-0.27 to 0.23)
Niger	184.91 (119.96 to 266.76)	204.9 (134.12 to 292.42)	0.11 (-0.01 to 0.24)	8.15 (4.06 to 14.1)	5.9 (4.07 to 8.35)	-0.28 (-0.49 to 0.22)	211.73 (108.41 to 339.62)	154.95 (107.16 to 220.35)	-0.27 (-0.48 to 0.25)
Nigeria	204.13 (142.71 to 274.15)	226.83 (157.97 to 304.7)	0.11 (0.08 to 0.14)	8.13 (4.54 to 11.85)	4.68 (3.3 to 6.08)	-0.42 (-0.61 to -0.03)	203.5 (116.63 to 293.26)	124.89 (91.99 to 161.38)	-0.39 (-0.58 to 0)
Niue	27.57 (20.42 to 37.31)	28.8 (21.03 to 38.37)	0.04 (-0.05 to 0.17)	3.22 (2.16 to 4.25)	2.88 (1.74 to 3.81)	-0.11 (-0.33 to 0.17)	96.3 (64.72 to 128.21)	87.68 (54.24 to 120.84)	-0.09 (-0.35 to 0.27)
North Macedonia	98.18 (71.14 to 131.44)	73.65 (53.33 to 99.88)	-0.25 (-0.34 to -0.15)	20.88 (11.64 to 28.02)	21.69 (11.36 to 31.57)	0.04 (-0.29 to 0.64)	317.32 (214.52 to 394.95)	335.05 (191.52 to 450.6)	0.06 (-0.23 to 0.43)
Northern Mariana Islands	67.41 (49.49 to 89.85)	67.56 (49.94 to 89.57)	0 (-0.12 to 0.13)	4.26 (3.23 to 6.77)	4 (3.02 to 6.07)	-0.06 (-0.3 to 0.29)	122.98 (91.78 to 188.78)	116.45 (87.08 to 172.62)	-0.05 (-0.3 to 0.32)
Norway	53.05 (40.78 to 68.6)	52.47 (41.46 to 65.12)	-0.01 (-0.1 to 0.09)	1.6 (1.2 to 3.78)	1.73 (1.44 to 2.64)	0.08 (-0.34 to 0.32)	45.86 (39.86 to 74.24)	39.04 (34.24 to 45.81)	-0.15 (-0.44 to -0.02)
Oman	14.95 (11.67 to 19.25)	15.75 (11.75 to 21.24)	0.05 (-0.09 to 0.21)	1.72 (1.05 to 2.98)	2.22 (1.14 to 3.06)	0.29 (-0.49 to 1.58)	44.12 (27.05 to 74.8)	54.7 (27.68 to 77.4)	0.24 (-0.56 to 1.6)
Pakistan	1.07 (0.74 to 1.51)	1.09 (0.75 to 1.53)	0.01 (-0.06 to 0.09)	0.29 (0.16 to 0.5)	0.3 (0.2 to 0.42)	0.02 (-0.26 to 0.4)	6.36 (3.48 to 10.35)	6.82 (4.55 to 9.56)	0.07 (-0.22 to 0.48)
Palau	11.6 (8.19 to 15.97)	11.61 (8.34 to 15.85)	0 (-0.1 to 0.12)	0.98 (0.71 to 1.34)	0.81 (0.58 to 1.06)	-0.17 (-0.41 to 0.16)	28.82 (19.88 to 40.65)	23.9 (16.97 to 31.66)	-0.17 (-0.43 to 0.19)
Palestine	38.39 (29.59 to 49.14)	40.31 (31.05 to 51.49)	0.05 (-0.05 to 0.14)	5.33 (2.85 to 7.88)	3 (2.12 to 3.7)	-0.44 (-0.64 to -0.1)	133.58 (75.63 to 193.73)	71.94 (55.19 to 89.69)	-0.46 (-0.64 to -0.11)

Panama	49.95 (36.47 to 68.25)	52.49 (38.44 to 70.01)	0.05 (-0.06 to 0.19)	3 (2.64 to 3.76)	4.34 (2.7 to 5.8)	0.45 (-0.2 to 1.02)	83.95 (75.46 to 104.89)	115.44 (71.53 to 156.29)	0.38 (-0.22 to 0.94)
Papua New Guinea	17.55 (12.82 to 23.7)	18.44 (13.39 to 25.06)	0.05 (-0.06 to 0.19)	2.72 (1.54 to 4.5)	3.14 (1.88 to 4.77)	0.16 (-0.13 to 0.62)	82.75 (42.63 to 144.43)	94.95 (52.41 to 155.89)	0.15 (-0.16 to 0.64)
Paraguay	33.33 (23.83 to 45.29)	36.52 (26.79 to 50.13)	0.1 (-0.02 to 0.23)	4.15 (2.44 to 5.34)	2.94 (2.18 to 4.09)	-0.29 (-0.52 to 0.25)	89.14 (59.27 to 109.48)	63.62 (47.34 to 91.22)	-0.29 (-0.52 to 0.21)
Peru	17.82 (12.75 to 24.53)	21.35 (15.89 to 28.04)	0.2 (0.06 to 0.37)	1.72 (1.22 to 2.14)	1.03 (0.72 to 1.55)	-0.4 (-0.62 to -0.01)	55.81 (36.27 to 71.17)	30.18 (21.46 to 42.98)	-0.46 (-0.66 to -0.04)
Philippines	34.84 (26.32 to 44.91)	35.04 (26.48 to 44.76)	0.01 (-0.02 to 0.03)	4.38 (1.44 to 5.95)	4.83 (1.83 to 6.63)	0.1 (-0.1 to 0.53)	61.24 (32.88 to 74.97)	88.01 (49.78 to 111.87)	0.44 (0.06 to 0.86)
Poland	131.28 (94.62 to 175.74)	141.93 (105.61 to 185.25)	0.08 (-0.04 to 0.22)	13.08 (10.46 to 13.98)	11.72 (7.09 to 14.18)	-0.1 (-0.37 to 0.05)	246.4 (211.46 to 265.77)	182.74 (127.62 to 218.55)	-0.26 (-0.44 to -0.12)
Portugal	43.74 (32.99 to 57.39)	29.64 (23.27 to 38.4)	-0.32 (-0.42 to -0.21)	3.73 (2.98 to 4.11)	2.2 (1.72 to 2.54)	-0.41 (-0.49 to -0.3)	73.03 (60.68 to 79.02)	41.48 (29.71 to 47.09)	-0.43 (-0.55 to -0.35)
Puerto Rico	43.17 (32.81 to 56.38)	38.62 (29.43 to 50.77)	-0.11 (-0.2 to 0)	4.42 (2.6 to 5.16)	1.88 (1.41 to 2.62)	-0.58 (-0.71 to -0.14)	108.3 (75.11 to 122.48)	47.45 (35.28 to 71.87)	-0.56 (-0.69 to -0.17)
Qatar	64.36 (50.54 to 81.22)	62.59 (46.62 to 81.83)	-0.03 (-0.21 to 0.17)	4.19 (2.53 to 5.98)	3.46 (2.2 to 4.87)	-0.17 (-0.49 to 0.33)	111.46 (78.31 to 149.7)	65.85 (46.45 to 89.07)	-0.41 (-0.62 to -0.1)
Republic of Korea	34.14 (25.76 to 44.64)	32.87 (25.31 to 42.36)	-0.04 (-0.14 to 0.09)	1.9 (1.36 to 2.64)	1.28 (0.87 to 1.52)	-0.33 (-0.58 to -0.04)	54.13 (40.32 to 80.49)	32 (23.81 to 37.55)	-0.41 (-0.65 to -0.18)
Republic of Moldova	14.62 (11.08 to 19.22)	26.72 (20.18 to 35.55)	0.83 (0.63 to 1.04)	2.83 (2.44 to 4.11)	1.96 (1.54 to 2.76)	-0.31 (-0.49 to -0.12)	105.36 (92.08 to 145.87)	66.32 (51.86 to 92.74)	-0.37 (-0.53 to -0.2)
Romania	91.18 (65.77 to 122.89)	75.84 (51.56 to 105.52)	-0.17 (-0.3 to -0.02)	25.67 (15.3 to 31.37)	17.57 (11.7 to 21.79)	-0.32 (-0.47 to -0.14)	381.17 (268.6 to 447.81)	323.51 (202.79 to 414.69)	-0.15 (-0.35 to 0.06)
Russian Federation	25.43 (19.75 to 32.62)	23.2 (17.84 to 30.18)	-0.09 (-0.13 to -0.05)	3.18 (2.35 to 6.64)	9.6 (3.55 to 12.34)	2.02 (-0.34 to 3.9)	89.12 (59.89 to 225.42)	303.74 (125.29 to 394.5)	2.41 (-0.34 to 5.21)

Rwanda	228.59 (150.63 to 325.65)	234 (154.24 to 328.21)	0.02 (-0.08 to 0.15)	5.62 (3.57 to 7.91)	3.96 (2.67 to 5.33)	-0.3 (-0.55 to 0.08)	200.21 (126.5 to 276.73)	137.78 (95.66 to 185.52)	-0.31 (-0.57 to 0.07)
Saint Kitts and Nevis	61.38 (46.59 to 81.35)	63.55 (48.33 to 83.71)	0.04 (-0.07 to 0.17)	12.84 (11.13 to 14.55)	10.54 (8.3 to 12.76)	-0.18 (-0.35 to 0.02)	333.73 (290.93 to 378.86)	246.06 (187.67 to 312.25)	-0.26 (-0.43 to -0.04)
Saint Lucia	51.75 (38.91 to 67.56)	54.08 (40.77 to 69.59)	0.05 (-0.08 to 0.17)	8.89 (7.52 to 10.81)	7.58 (6.06 to 9.18)	-0.15 (-0.36 to 0.08)	212.01 (182.06 to 259.19)	178.22 (142.41 to 216.58)	-0.16 (-0.39 to 0.06)
Saint Vincent and the Grenadines	15.69 (11.74 to 20.89)	17.25 (12.9 to 22.68)	0.1 (-0.01 to 0.22)	2.74 (2.35 to 3.67)	3.01 (2.37 to 3.57)	0.1 (-0.29 to 0.42)	72.62 (63.45 to 93.47)	72.06 (56.84 to 86.86)	-0.01 (-0.32 to 0.25)
Samoa	26.53 (19.63 to 35.42)	26.85 (19.76 to 35.77)	0.01 (-0.1 to 0.13)	3.41 (2.3 to 4.47)	3.16 (1.91 to 4.28)	-0.07 (-0.31 to 0.26)	95.25 (64.23 to 131.06)	87.56 (53.05 to 122.19)	-0.08 (-0.36 to 0.32)
San Marino	103.2 (78.48 to 133.83)	75.24 (56.62 to 96.9)	-0.27 (-0.35 to -0.19)	5.88 (3.72 to 7.62)	4.77 (3.09 to 6.63)	-0.19 (-0.48 to 0.3)	100.25 (69.82 to 123.86)	76.88 (51.33 to 111.11)	-0.23 (-0.5 to 0.23)
Sao Tome and Principe	230.15 (150.15 to 324.13)	261.03 (169.46 to 366.92)	0.13 (0.01 to 0.26)	6.09 (3.33 to 8.59)	5.07 (3.6 to 6.52)	-0.17 (-0.44 to 0.32)	163.44 (97.29 to 225.39)	140.82 (103.71 to 181.26)	-0.14 (-0.4 to 0.37)
Saudi Arabia	63.25 (47 to 83.9)	76.37 (56.9 to 100.8)	0.21 (0.07 to 0.37)	7.48 (5.04 to 11.22)	5.01 (3.18 to 6.31)	-0.33 (-0.6 to -0.01)	182.87 (131.09 to 265.92)	118.77 (78.67 to 151.06)	-0.35 (-0.59 to -0.05)
Senegal	265.02 (174.81 to 363.09)	266.59 (176.77 to 370.58)	0.01 (-0.1 to 0.12)	6.88 (3.64 to 9.9)	5.11 (3.5 to 6.73)	-0.26 (-0.48 to 0.22)	183.81 (105.11 to 252.36)	142.39 (101.91 to 183.48)	-0.23 (-0.47 to 0.25)
Serbia	75.85 (53.37 to 102.93)	46.78 (32.29 to 63.12)	-0.38 (-0.52 to -0.23)	19.64 (12.24 to 25.37)	19 (10.09 to 26.24)	-0.03 (-0.32 to 0.3)	306.66 (219.77 to 372.66)	260.29 (149.29 to 354.52)	-0.15 (-0.4 to 0.12)
Seychelles	68.84 (51.17 to 92.24)	64.36 (48.63 to 85.57)	-0.07 (-0.18 to 0.05)	8.82 (7.02 to 11.08)	7.6 (6.07 to 9.65)	-0.14 (-0.31 to 0.05)	228.37 (182.8 to 289.13)	197.43 (158.11 to 250.65)	-0.14 (-0.3 to 0.05)
Sierra Leone	238.02 (155.22 to 338.67)	248.56 (162.78 to 351.92)	0.04 (-0.08 to 0.17)	7.19 (3.79 to 10.51)	5.34 (3.63 to 7.64)	-0.26 (-0.5 to 0.23)	192.14 (108.09 to 273.62)	154.24 (108.22 to 218.36)	-0.2 (-0.46 to 0.32)
Singapore	32.95 (25.32 to 42.63)	25.42 (19.64 to 32.97)	-0.23 (-0.31 to -0.15)	2.91 (2.47 to 4.1)	1.37 (1.03 to 1.96)	-0.53 (-0.68 to -0.4)	96.5 (79.96 to 130.69)	38.72 (29.9 to 49.02)	-0.6 (-0.75 to -0.46)

Slovakia	42.47 (31.51 to 56.13)	53.47 (40.06 to 69.65)	0.26 (0.01 to 0.61)	4.11 (2.94 to 7.96)	3.64 (2.55 to 6.11)	-0.11 (-0.36 to 0.25)	111.6 (94.25 to 162.66)	86.33 (64.16 to 114.61)	-0.23 (-0.45 to 0.05)
Slovenia	221.47 (154.14 to 304.69)	154.71 (114.94 to 200.38)	-0.3 (-0.42 to -0.13)	55.89 (9.95 to 77.19)	14.38 (8.32 to 18.5)	-0.74 (-0.82 to -0.03)	764.79 (188.88 to 1066.75)	196.21 (141.85 to 249.35)	-0.74 (-0.83 to -0.11)
Solomon Islands	15.43 (11 to 21.22)	15.09 (10.79 to 20.51)	-0.02 (-0.14 to 0.1)	3.63 (2.05 to 5.91)	3.67 (2.06 to 5.75)	0.01 (-0.25 to 0.37)	109.53 (56.84 to 190.85)	108.38 (57.26 to 184.06)	-0.01 (-0.28 to 0.35)
Somalia	222.13 (144.91 to 322.75)	231.23 (153.1 to 326.33)	0.04 (-0.07 to 0.17)	5.14 (3.2 to 7.42)	4.14 (2.71 to 6.16)	-0.19 (-0.44 to 0.21)	190.78 (116.46 to 277.71)	153.56 (103.57 to 217.62)	-0.2 (-0.44 to 0.18)
South Africa	273.59 (182.9 to 383.62)	243.82 (162.25 to 336.95)	-0.11 (-0.14 to -0.07)	11.69 (8.97 to 14.24)	10.53 (8.74 to 13.28)	-0.1 (-0.23 to 0.05)	283.51 (229.07 to 330.37)	230.12 (192.18 to 287.08)	-0.19 (-0.28 to -0.03)
South Sudan	246.93 (163.29 to 353.28)	256.85 (172.61 to 361.95)	0.04 (-0.07 to 0.16)	4.56 (2.42 to 6.58)	3.14 (1.91 to 4.71)	-0.31 (-0.5 to 0.02)	181.23 (100.34 to 259.13)	126.14 (79.72 to 185.63)	-0.3 (-0.51 to 0.03)
Spain	118.68 (88.66 to 154.22)	79.47 (59.71 to 102.71)	-0.33 (-0.42 to -0.25)	9.8 (6 to 11.19)	5.01 (4.09 to 5.74)	-0.49 (-0.56 to -0.25)	158.82 (126.46 to 173.75)	91.79 (74.78 to 103.91)	-0.42 (-0.48 to -0.35)
Sri Lanka	114.07 (84.63 to 151.44)	122.31 (90.11 to 161.35)	0.07 (-0.04 to 0.2)	14.48 (9.15 to 17.54)	7.02 (4.83 to 10.85)	-0.52 (-0.7 to -0.11)	343.22 (229.72 to 406.35)	179.86 (128.23 to 260.13)	-0.48 (-0.66 to -0.13)
Sudan	12.82 (9.46 to 17.28)	14.26 (10.54 to 18.74)	0.11 (0 to 0.24)	2.77 (1.46 to 4.99)	2.19 (1.44 to 3.54)	-0.21 (-0.45 to 0.17)	85.45 (39.43 to 193.33)	62.52 (38.19 to 107.67)	-0.27 (-0.55 to 0.24)
Suriname	37.01 (28.15 to 49.51)	40.16 (30.6 to 52.64)	0.08 (-0.05 to 0.21)	5.04 (4.07 to 7.08)	4.98 (3.78 to 6.3)	-0.01 (-0.26 to 0.3)	149.8 (120.63 to 199.31)	139.92 (105.37 to 177.65)	-0.07 (-0.3 to 0.24)
Sweden	95.75 (76.16 to 118.57)	92.41 (72.34 to 118.71)	-0.03 (-0.14 to 0.09)	3.78 (2.99 to 5.9)	2.19 (1.67 to 4.41)	-0.42 (-0.54 to -0.15)	106.02 (82.02 to 118.21)	50.9 (42.91 to 73.63)	-0.52 (-0.61 to -0.28)
Switzerland	46.25 (35.33 to 59.03)	31.05 (25.77 to 37.56)	-0.33 (-0.43 to -0.19)	3.07 (2.55 to 3.94)	2.07 (1.66 to 3.17)	-0.32 (-0.46 to -0.06)	59.89 (50.72 to 71.75)	36.2 (31.03 to 50.44)	-0.4 (-0.51 to -0.2)
Syrian Arab Republic	20.83 (15.41 to 27.75)	23.66 (17.52 to 31.61)	0.14 (0.01 to 0.26)	3.18 (2.2 to 4.83)	2.38 (1.7 to 3.72)	-0.25 (-0.48 to 0.06)	98.96 (66.51 to 156.26)	62.3 (43.17 to 98.07)	-0.37 (-0.59 to -0.05)

Taiwan (Province of China)	43.24 (32.18 to 56)	37.96 (29.31 to 47.33)	-0.12 (-0.23 to 0)	2.35 (1.78 to 2.59)	1.19 (0.91 to 1.6)	-0.5 (-0.62 to -0.25)	55.32 (45.1 to 60.11)	32.86 (25.14 to 42.96)	-0.41 (-0.54 to -0.21)
Tajikistan	4.65 (3.44 to 6.14)	4.5 (3.37 to 5.96)	-0.03 (-0.12 to 0.06)	0.92 (0.62 to 1.16)	1.09 (0.68 to 1.42)	0.18 (-0.1 to 0.55)	29.23 (21.23 to 35.5)	31.46 (20.82 to 40.69)	0.08 (-0.19 to 0.43)
Thailand	17.66 (12.92 to 23.52)	19.27 (14.11 to 25.58)	0.09 (-0.01 to 0.2)	0.6 (0.36 to 1.09)	0.6 (0.42 to 0.9)	0 (-0.38 to 0.7)	16.26 (10.58 to 29.5)	18.71 (13.34 to 24.98)	0.15 (-0.35 to 0.91)
Timor-Leste	27.58 (20.69 to 36.59)	32.92 (24.42 to 43.61)	0.19 (0.06 to 0.35)	2.76 (1.76 to 4.03)	3.76 (2.59 to 5.17)	0.36 (-0.07 to 0.94)	58.87 (36.56 to 106.17)	76.72 (53.69 to 111.88)	0.3 (-0.14 to 0.89)
Togo	229.95 (150.52 to 324.56)	231.97 (151.87 to 327.21)	0.01 (-0.1 to 0.13)	7.74 (4.22 to 10.85)	5.2 (3.69 to 6.97)	-0.33 (-0.53 to 0.06)	201.53 (116.42 to 271.5)	146.28 (107.38 to 195.18)	-0.27 (-0.49 to 0.12)
Tokelau	26.4 (19.5 to 35.12)	28.58 (21.17 to 37.94)	0.08 (-0.02 to 0.21)	3.18 (2.09 to 4.32)	2.77 (1.66 to 3.81)	-0.13 (-0.38 to 0.23)	89.3 (57.88 to 125)	77.69 (45.77 to 109.79)	-0.13 (-0.4 to 0.31)
Tonga	26.54 (19.44 to 35.7)	26.35 (19.45 to 35.66)	-0.01 (-0.11 to 0.13)	1.69 (1.26 to 2.15)	1.61 (1.06 to 2.1)	-0.05 (-0.29 to 0.29)	49.31 (37.78 to 63.09)	47.67 (32.81 to 63.67)	-0.03 (-0.29 to 0.34)
Trinidad and Tobago	30.36 (23.24 to 39.26)	30.51 (23.25 to 40.57)	0 (-0.1 to 0.12)	4.81 (3.93 to 5.85)	3.75 (2.79 to 4.93)	-0.22 (-0.44 to 0.08)	132.55 (112.72 to 155.72)	106.39 (77.1 to 141.99)	-0.2 (-0.43 to 0.11)
Tunisia	21.37 (15.84 to 28.16)	23.27 (17.6 to 30.54)	0.09 (-0.01 to 0.2)	2.49 (1.86 to 3.66)	1.78 (1.23 to 2.76)	-0.28 (-0.5 to 0)	70.25 (51.27 to 105.8)	44.05 (30.23 to 69.43)	-0.37 (-0.57 to -0.09)
Turkey	33.82 (25.93 to 43.94)	27.86 (22.65 to 34.21)	-0.18 (-0.3 to -0.03)	2.6 (1.86 to 3.95)	1.33 (0.95 to 2.22)	-0.49 (-0.65 to -0.25)	87.65 (63.08 to 123.9)	41.59 (33.07 to 56.27)	-0.53 (-0.67 to -0.29)
Turkmenistan	36.27 (26.88 to 48.65)	38.07 (28.03 to 51.45)	0.05 (-0.06 to 0.17)	17.5 (12.77 to 20.98)	13.22 (9.87 to 19.45)	-0.24 (-0.47 to 0.27)	611.77 (441.08 to 727.68)	487.39 (370.57 to 674.68)	-0.2 (-0.42 to 0.29)
Tuvalu	20.96 (15.5 to 28.6)	22.88 (16.68 to 31.12)	0.09 (-0.02 to 0.22)	3.94 (2.38 to 5.6)	3.47 (2.08 to 4.85)	-0.12 (-0.38 to 0.29)	117.09 (67.26 to 180.52)	99.4 (60.2 to 142.04)	-0.15 (-0.44 to 0.33)
Uganda	176.5 (107.68 to 259.67)	172.7 (105.71 to 253.33)	-0.02 (-0.13 to 0.09)	4.07 (2.72 to 5.3)	3.54 (2.16 to 4.68)	-0.13 (-0.36 to 0.17)	143.77 (96.63 to 186.98)	125.04 (76.79 to 172.8)	-0.13 (-0.35 to 0.18)

Ukraine	23.7 (18.49 to 30.5)	21.7 (16.67 to 27.88)	-0.08 (-0.19 to 0)	4.79 (3.45 to 6.61)	7.64 (5.73 to 9.48)	0.59 (0.1 to 1.27)	122.74 (91.14 to 185.2)	211.95 (162.8 to 291.69)	0.73 (0.21 to 1.43)
United Arab Emirates	18.9 (14.49 to 24.82)	18.94 (14.2 to 24.74)	0 (-0.09 to 0.11)	2.45 (1.28 to 4.77)	1.58 (0.77 to 3.24)	-0.35 (-0.54 to -0.07)	68.82 (39.5 to 128.21)	42.17 (23.05 to 82.4)	-0.39 (-0.57 to -0.09)
United Kingdom	40.35 (31.62 to 50.66)	33.48 (27.19 to 40.76)	-0.17 (-0.24 to -0.09)	2.61 (2.14 to 4.84)	2.45 (2.09 to 3.42)	-0.06 (-0.37 to 0.11)	72.55 (62.68 to 95.33)	51.83 (47.63 to 61.31)	-0.29 (-0.44 to -0.17)
United Republic of Tanzania	257.74 (168.95 to 355.4)	286.03 (186.1 to 397.37)	0.11 (-0.01 to 0.25)	5.13 (3.51 to 6.35)	4.17 (2.62 to 5.83)	-0.19 (-0.42 to 0.15)	191.28 (132.2 to 244.8)	159.66 (103.17 to 236.25)	-0.17 (-0.41 to 0.22)
United States of America	153.61 (120.2 to 196.76)	157.1 (126.15 to 193.65)	0.02 (-0.09 to 0.15)	6.19 (4.79 to 6.65)	4.92 (4.35 to 5.44)	-0.21 (-0.28 to -0.04)	170.88 (132.99 to 184.92)	125.27 (114.44 to 140.71)	-0.27 (-0.34 to -0.07)
United States Virgin Islands	39.65 (30.53 to 51.55)	37.37 (28.39 to 49.12)	-0.06 (-0.15 to 0.04)	7.1 (5.61 to 9.3)	6.99 (5.07 to 8.69)	-0.02 (-0.26 to 0.29)	181.46 (143.35 to 234.95)	159.2 (113.65 to 206.08)	-0.12 (-0.36 to 0.18)
Uruguay	115.86 (82.18 to 158.35)	91.18 (65.97 to 123.07)	-0.21 (-0.29 to -0.13)	9.51 (6.19 to 10.99)	4.66 (3.92 to 6.57)	-0.51 (-0.62 to -0.06)	214.91 (146.15 to 245.32)	101.88 (88.11 to 142.04)	-0.53 (-0.62 to -0.14)
Uzbekistan	1.97 (1.45 to 2.59)	2.1 (1.55 to 2.78)	0.07 (-0.04 to 0.18)	0.61 (0.28 to 1.25)	1.3 (1.03 to 1.69)	1.13 (0.19 to 3.51)	19.17 (10.75 to 39.37)	42.95 (33.05 to 53.54)	1.24 (0.14 to 2.9)
Vanuatu	21.72 (15.91 to 29.6)	20.51 (15.06 to 27.6)	-0.06 (-0.17 to 0.06)	3.19 (1.96 to 4.63)	3.5 (1.97 to 5.1)	0.1 (-0.19 to 0.49)	88.6 (53.01 to 136.5)	102.87 (56.79 to 155.24)	0.16 (-0.16 to 0.64)
Venezuela (Bolivarian Republic of)	17.57 (12.22 to 24.64)	16.32 (11.38 to 22.85)	-0.07 (-0.17 to 0.06)	4.17 (1.8 to 5.17)	2.66 (1.8 to 3.47)	-0.36 (-0.54 to 0.18)	86.86 (47.85 to 103.92)	53.78 (40.44 to 69.25)	-0.38 (-0.55 to 0.1)
Viet Nam	42.36 (31.59 to 54.68)	49.24 (38.37 to 63.27)	0.16 (0.02 to 0.35)	4.21 (3.04 to 6.18)	3.93 (2.91 to 5.2)	-0.07 (-0.33 to 0.32)	93.64 (68.96 to 150.26)	82.57 (60.54 to 122.5)	-0.12 (-0.35 to 0.21)
Yemen	11.81 (8.76 to 15.72)	12.98 (9.65 to 17.22)	0.1 (0 to 0.22)	2.77 (1.48 to 5.18)	2.31 (1.43 to 4.03)	-0.17 (-0.43 to 0.24)	76.09 (38.7 to 169.97)	60.37 (36.18 to 112.02)	-0.21 (-0.48 to 0.27)
Zambia	242.02 (153.24 to 343.92)	257.45 (168.15 to 367.05)	0.06 (-0.05 to 0.19)	3.94 (2.33 to 5.26)	4.69 (2.79 to 6.45)	0.19 (-0.14 to 0.67)	152.24 (92.93 to 207.44)	160.1 (100.87 to 222.45)	0.05 (-0.26 to 0.49)

Zimbabwe	227.25 (158.74 to 310.57)	226.73 (147.99 to 327.72)	0 (-0.2 to 0.22)	14.81 (10.95 to 17.82)	15.12 (10.92 to 19.33)	0.02 (-0.22 to 0.3)	327.9 (239.71 to 392.24)	347.53 (248.2 to 443.37)	0.06 (-0.19 to 0.35)

DALY, Disability-Adjusted Life Year; UI, uncertainty interval.
