# Supplementary figures and images for: National, regional, and global cardiomyopathy burden from 1990 to 2019
Source: Front Cardiovasc Med. 2022 Nov 30;9:1042448. doi: 10.3389/fcvm.2022.1042448 (PMC9748073; doi:10.3389/fcvm.2022.1042448)

A

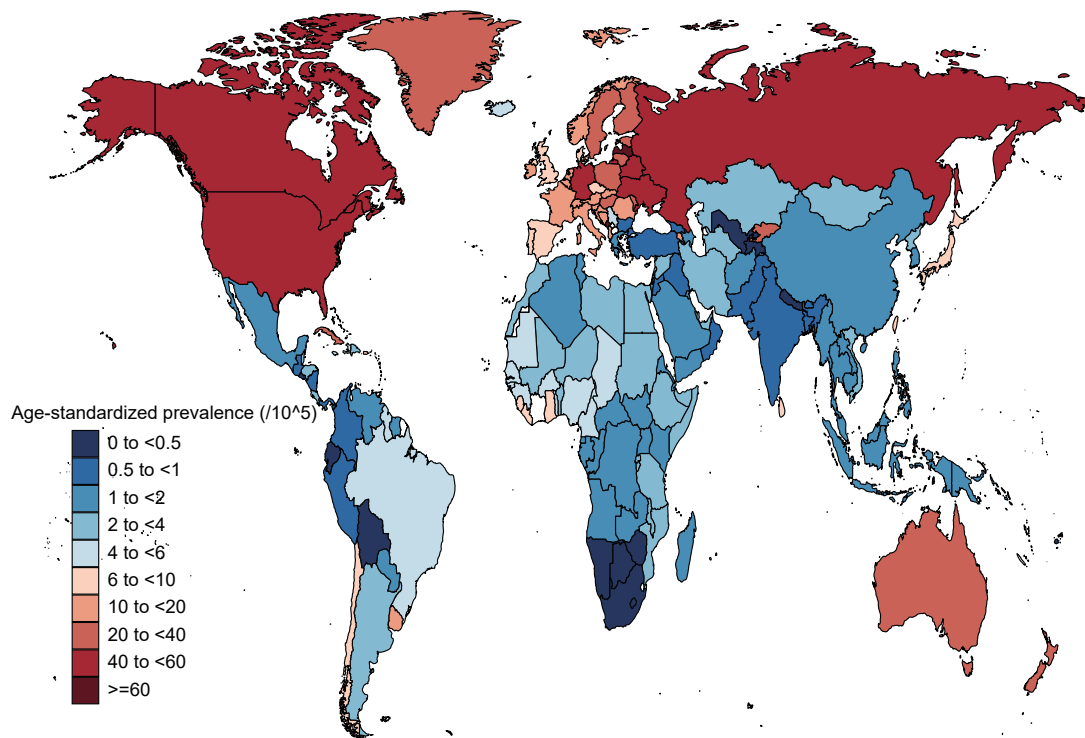

B

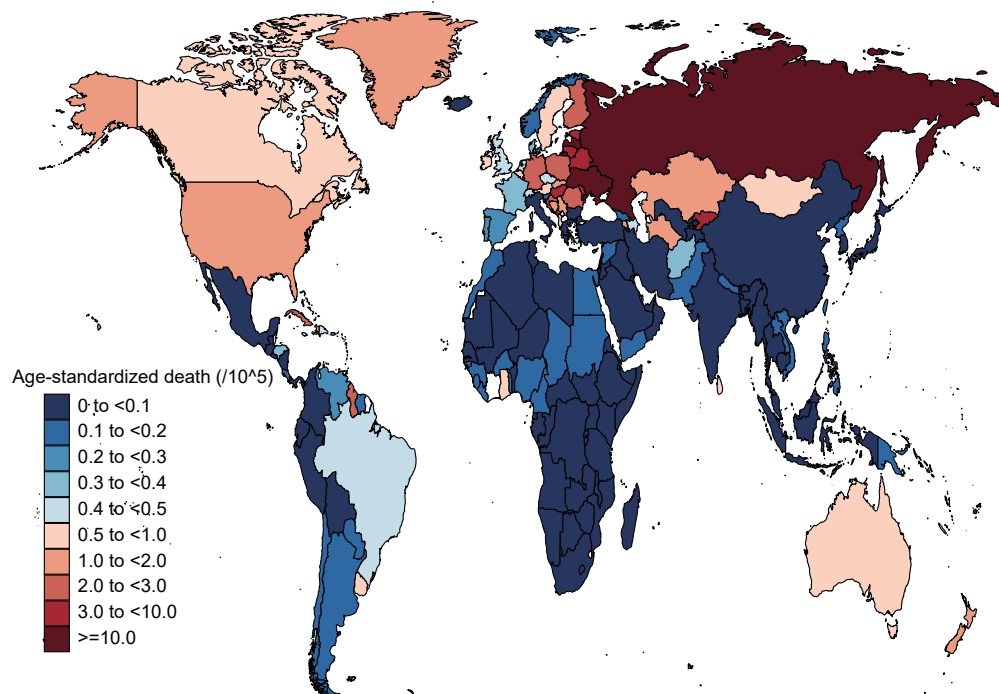

C

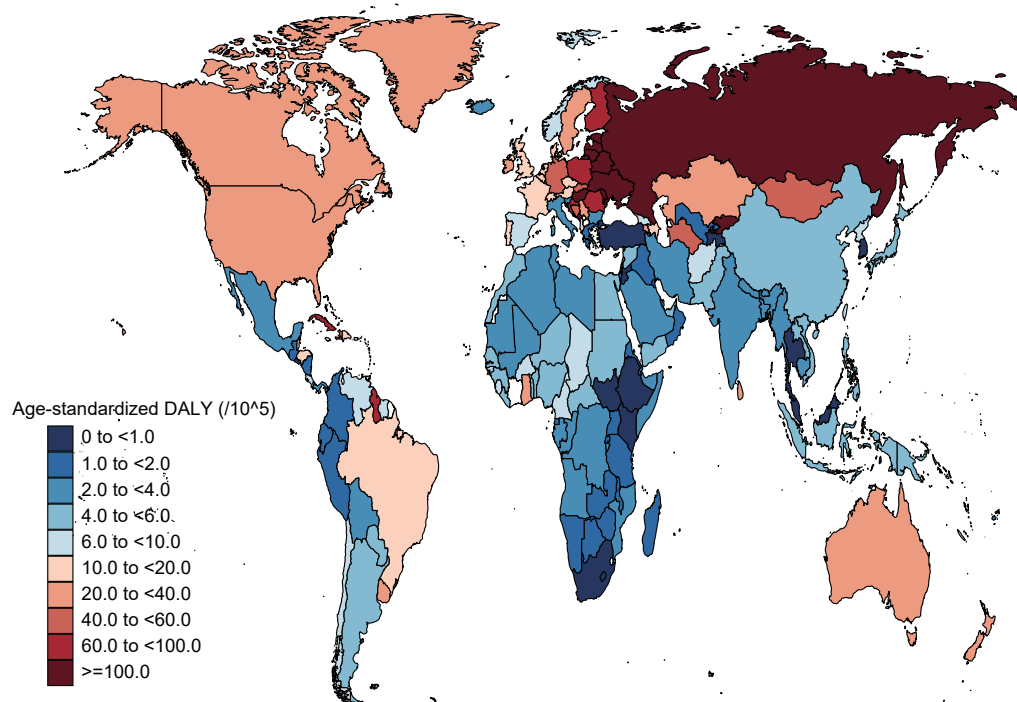

Supplement: Supplementary file 2 [file Data_Sheet_2.PDF]

A

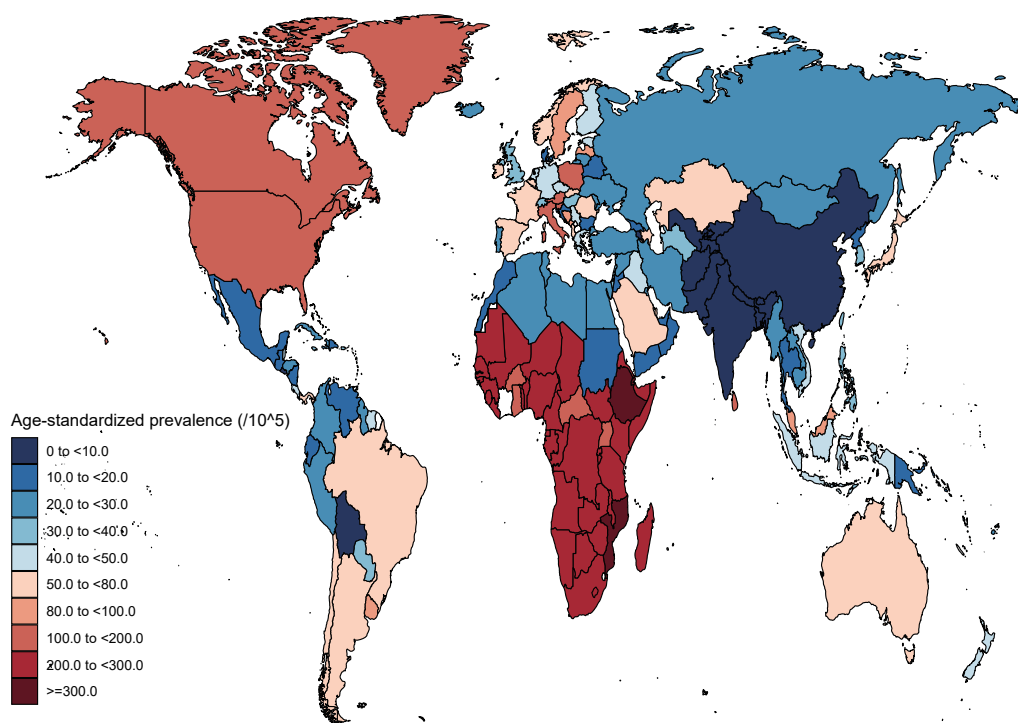

B

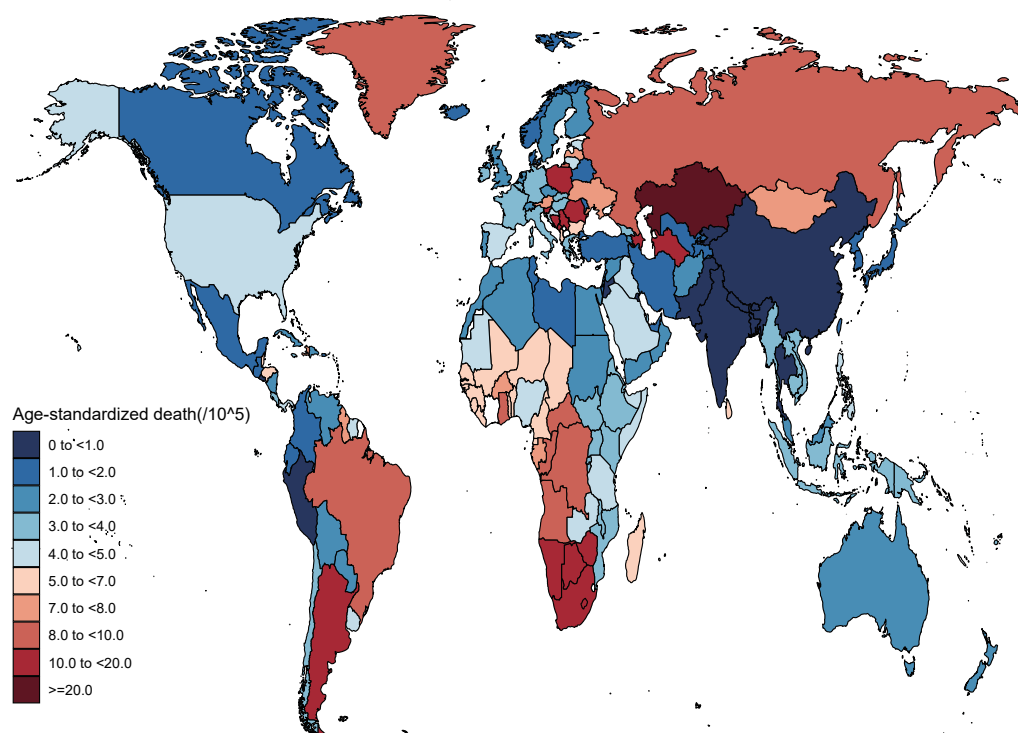

C

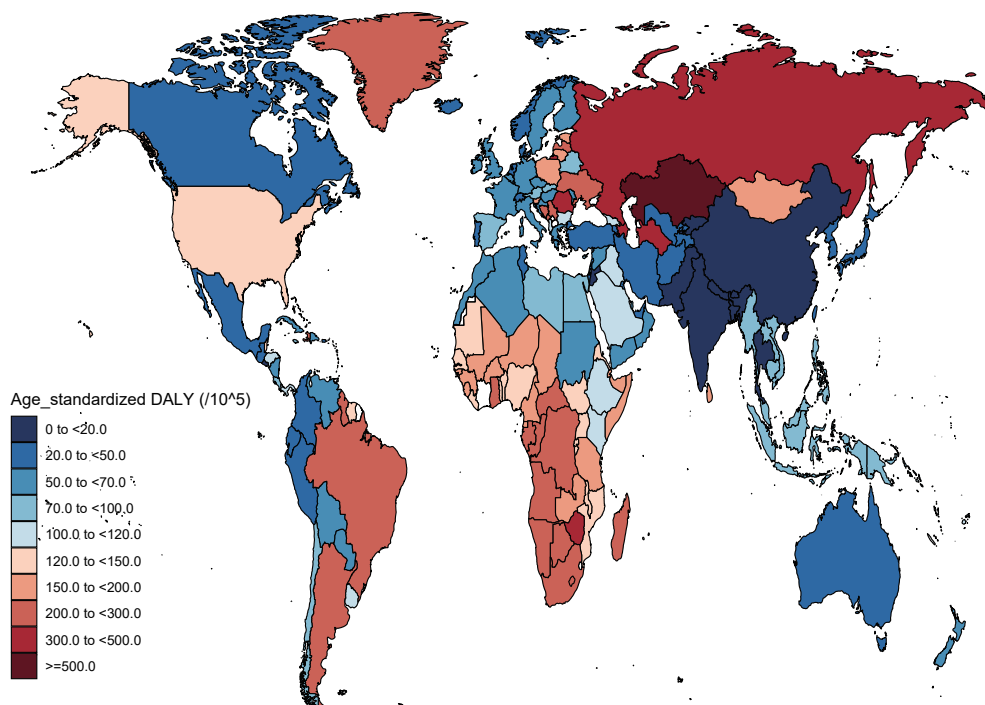

Supplement: Supplementary file 3 [file Data_Sheet_3.PDF]

Alcoholic cardiomyopathy    Other cardiomyopathy

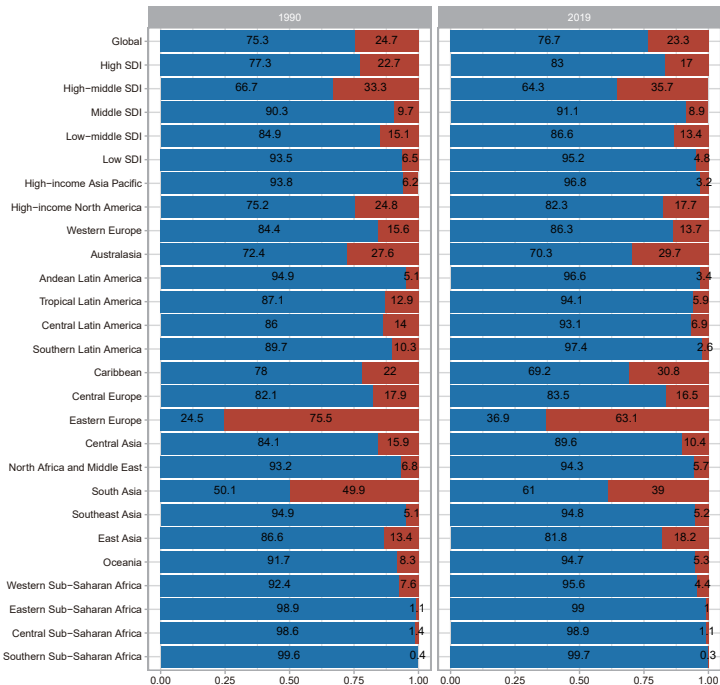

Supplement: Supplementary file 4 [file Data_Sheet_4.PDF]

Alcoholic cardiomyopathy Other cardiomyopathy

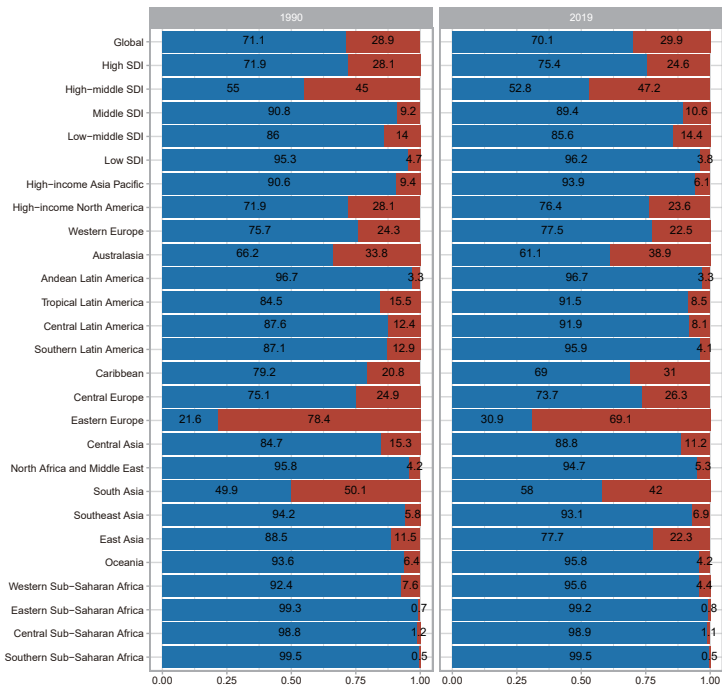

Supplement: Supplementary file 5 [file Data_Sheet_5.pdf]

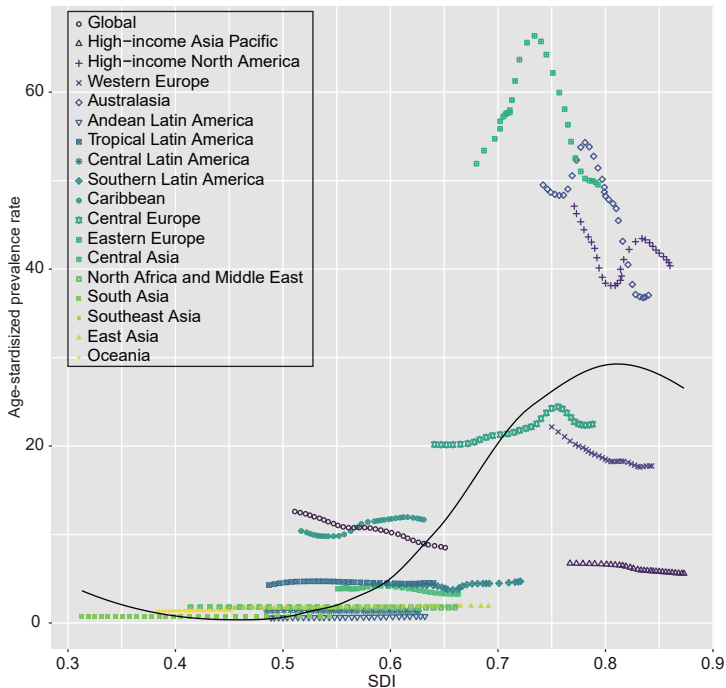

Supplement: Supplementary file 6 [file Data_Sheet_6.pdf]

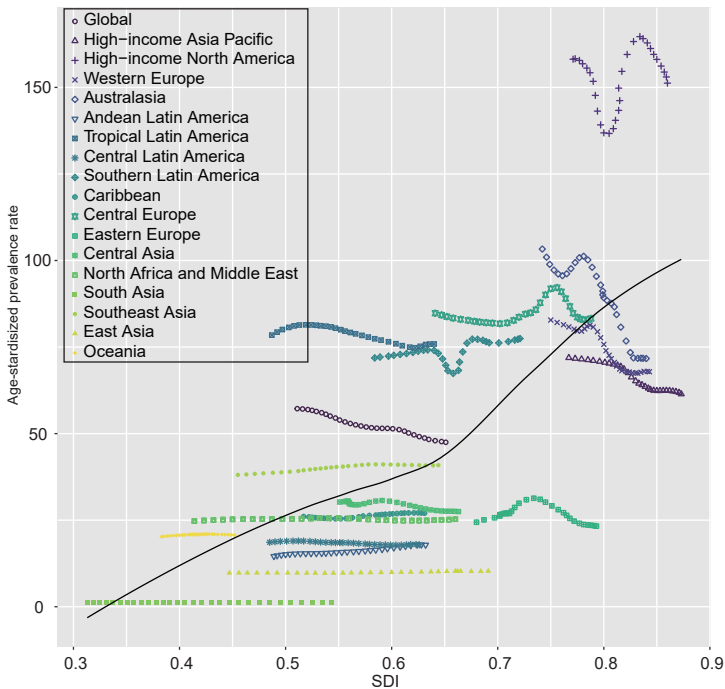

Supplement: Supplementary file 7 [file Data_Sheet_7.pdf]
